# Supplementary material for: Selective molecular inhibition of the HDAC6 ZnF-UBP binding domain impairs multiple myeloma cell function
Source: Cell Death Discov. 2025 Apr 15;11:176. doi: 10.1038/s41420-025-02465-1 (PMC12000391; doi:10.1038/s41420-025-02465-1)
Supplement: Supplementary file 1 — Supplementary information [file 41420_2025_2465_MOESM1_ESM.docx]

**Supplementary information**

**Selective molecular inhibition of the HDAC6 ZnF-UBP binding domain impairs multiple myeloma cell proliferation**

Rafael Rincón ^1,2,3^, Isabel F. Coira ^1,2,3^, Antoine Richieu ^4,5^, Fedaa Attana ^6^, Muriel Urwyler ^1,2,3^, Shozeb Haider ^6,7^, Carole Bourquin ^1,2,3,8,9^, Philippe Bertrand ^4,5^, Muriel Cuendet ^1,2,3,*^

^1^School of Pharmaceutical Sciences, University of Geneva, Switzerland

^2^Institute of Pharmaceutical Sciences of Western Switzerland, University of Geneva, Switzerland

^3^Translational Research Center in Oncohaematology, University of Geneva, Switzerland

^4^Institut de Chimie des Milieux et Matériaux de Poitiers, UMR CNRS 7285, France

^5^Réseau Epigénétique du Cancéropôle Grand Ouest, France

^6^UCL School of Pharmacy, University College London, UK

^7^UCL Centre for Advanced Research Computing, University College London, UK

^8^Department of Anesthesiology, Pharmacology, Intensive Care and Emergency Medicine, University of Geneva, Switzerland

^9^Institute of Pharmacology, University of Bern, Switzerland

**Correspondence to:** Prof. Muriel Cuendet, School of Pharmaceutical Sciences, University of Geneva, Rue Michel Servet 1, CH-1211 Geneva 4, Switzerland. Email: [Muriel.Cuendet@unige.ch](mailto:Muriel.Cuendet@unige.ch) Phone: +41 22 379 33 86

**Table S1**. sgRNA, ssDNA and primer sequences (5’ – 3’) for CRISPR-Cas9 edition **4**

**Fig. S1**. ^1^H NMR (400 MHz, CDCl_3_) spectrum of compound **5b** **5**

**Fig. S2**. ^13^C NMR (100 MHz, CDCl_3_) spectrum of compound **5b** **5**

**Fig. S3**. HRMS spectrum of compound **5b** **6**

**Fig. S4**. ^1^H NMR (400 MHz, CDCl_3_) spectrum of compound **6b** **6**

**Fig. S5**. ^13^C NMR (100 MHz, CDCl_3_) spectrum of compound **6b** **7**

**Fig. S6**. HRMS spectrum of compound **6b** **7**

**Fig. S7**. IR spectrum of compound **1b** **8**

**Fig. S8**. ^1^H NMR (400 MHz, DMSO-*d_6_*) spectrum of compound **1b** **8**

**Fig. S9**. ^13^C NMR (100 MHz, DMSO-*d_6_*) spectrum of compound **1b** **9**

**Fig. S10**. HRMS spectrum of compound **1b** **9**

**Fig. S11**. HPLC-UV spectrum of compound **1b** **10**

**Fig. S12**. ^1^H NMR (400 MHz, DMSO-*d_6_*) spectrum of compound **5c** **10**

**Fig. S13**. ^13^C NMR (100 MHz, DMSO-*d_6_*) spectrum of compound **5c** **11**

**Fig. S14**. HRMS spectrum of compound **5c** **11**

**Fig. S15**. ^1^H NMR (400 MHz, CDCl_3_) spectrum of compound **6c** **12**

**Fig. S16**. ^13^C NMR (100 MHz, CDCl_3_) spectrum of compound **6c** **12**

**Fig. S17**. HRMS spectrum of compound **6c** **13**

**Fig. S18**. IR spectrum of compound **1c** **13**

**Fig. S19**. ^1^H NMR (400 MHz, DMSO-*d_6_*) spectrum of compound **1c** **14**

**Fig. S20**. ^13^C NMR (100 MHz, DMSO-*d_6_*) spectrum of compound **1c** **14**

**Fig. S21**. HRMS spectrum of compound **1c** **15**

**Fig. S22**. HPLC-UV spectrum of compound **1c** **15**

**Fig. S23**. ^1^H NMR (400 MHz, DMSO-*d_6_*) spectrum of compound **5d** **16**

**Fig. S24**. ^13^C NMR (100 MHz, DMSO-*d_6_*) spectrum of compound **5d** **16**

**Fig. S25**. HRMS spectrum of compound **5d** **17**

**Fig. S26**. ^1^H NMR (400 MHz, CDCl_3_) spectrum of compound **6d** **17**

**Fig. S27**. ^13^C NMR (100 MHz, CDCl_3_) spectrum of compound **6d** **18**

**Fig. S28**. HRMS spectrum of compound **6d** **18**

**Fig. S29**. IR spectrum of compound **1d** **19**

**Fig. S30**. ^1^H NMR (400 MHz, DMSO-*d_6_*) spectrum of compound **1d** **19**

**Fig. S31**. ^13^C NMR (125 MHz, DMSO-*d_6_*) spectrum of compound **1d** **20**

**Fig. S32**. HRMS spectrum of compound **1d** **20**

**Fig. S33**. HPLC-UV spectrum of compound **1d** **21**

**Fig. S34**. ^1^H NMR (400 MHz, DMSO-*d_6_*) spectrum of compound **5e** **21**

**Fig. S35**. ^13^C NMR (100 MHz, DMSO-*d_6_*) spectrum of compound **5e** **22**

**Fig. S36**. HRMS spectrum of compound **5e** **22**

**Fig. S37**. ^1^H NMR (400 MHz, CDCl_3_) spectrum of compound **6e** **23**

**Fig. S38**. ^13^C NMR (100 MHz, CDCl_3_) spectrum of compound **6e** **23**

**Fig. S39**. HRMS spectrum of compound **6e** **24**

**Fig. S40**. IR spectrum of compound **1e** **24**

**Fig. S41**. ^1^H NMR (500 MHz, DMSO-*d_6_*) spectrum of compound **1e** **25**

**Fig. S42**. ^13^C NMR (100 MHz, DMSO-*d_6_*) spectrum of compound **1e** **25**

**Fig. S43**. HRMS spectrum of compound **1e** **26**

**Fig. S44**. HPLC-UV spectrum of compound **1e** **26**

**Fig. S45**. ^1^H NMR (400 MHz, DMSO-*d_6_*) spectrum of compound **5f** **27**

**Fig. S46**. ^13^C NMR (100 MHz, DMSO-*d_6_*) spectrum of compound **5f** **27**

**Fig. S47**. HRMS spectrum of compound **5f** **28**

**Fig. S48**. ^1^H NMR (400 MHz, CDCl_3_) spectrum of compound **6f** **28**

**Fig. S49**. ^13^C NMR (100 MHz, CDCl_3_) spectrum of compound **6f** **29**

**Fig. S50**. HRMS spectrum of compound **6f** **29**

**Fig. S51**. IR spectrum of compound **1f** **30**

**Fig. S52**. ^1^H NMR (400 MHz, DMSO-*d_6_*) spectrum of compound **1f** **30**

**Fig. S53**. ^13^C NMR (100 MHz, DMSO-*d_6_*) spectrum of compound **1f** **31**

**Fig. S54**. HRMS spectrum of compound **1f** **31**

**Fig. S55**. HPLC-UV spectrum of compound **1f** **32**

**Fig. S56**. ^1^H NMR (400 MHz, CDCl_3_) spectrum of compound **5g** **32**

**Fig. S57**. ^13^C NMR (100 MHz, CDCl_3_) spectrum of compound **5g** **33**

**Fig. S58**. HRMS spectrum of compound **5g** **33**

**Fig. S59**. ^1^H NMR (400 MHz, CDCl_3_) spectrum of compound **6g** **34**

**Fig. S60**. ^13^C NMR (100 MHz, CDCl_3_) spectrum of compound **6g** **34**

**Fig. S61**. HRMS spectrum of compound **6g** **35**

**Fig. S62**. IR spectrum of compound **1g** **35**

**Fig. S63**. ^1^H NMR (400 MHz, DMSO-*d_6_*) spectrum of compound **1g** **36**

**Fig. S64**. ^13^C NMR (125 MHz, DMSO-*d_6_*) spectrum of compound **1g** **36**

**Fig. S65**. HRMS spectrum of compound **1g** **37**

**Fig. S66**. HPLC-UV spectrum of compound **1g** **37**

**Table S1**. sgRNA, ssDNA and primer sequences (5’ – 3’) for CRISPR-Cas9 edition.

| **sgRNA names** | **sgRNA sequence** | **Targeted exon** |
| --- | --- | --- |
| sgRNA_KO | AAAGGCAAAATGAAGAAGCT | Exon 3 |
| sgRNA_RY | TACTGTGGTCGTTACATCAA | Exon 28 |
| **ssDNA name** | **ssDNA sequence** | **Induced mutation** |
| ssDNA_RY | CCTGACACTCACACCCCCAACCTCAGGTCTACTGTGGTGCTGCCATCAATGGCCACATGCTCCAACACCATGGAAATTCTGGAC | R1155A, Y1156A |
| **Primer names for PCR and sequencing** | **Primer sequence** | **Amplified exon** |
| KO_Fw | ATTCCACCACAACCAGGCAG | Exon 3 (433 bp) |
| KO_Rv | GCCTCAAGGTTCAGATCCTGC |  |
| RY_Fw | AGGCCTAGACGTGACCCAAC | Exon 28 (412 bp) |
| RY_Rv | CATGAGGCAGCCATGCAGAG |  |


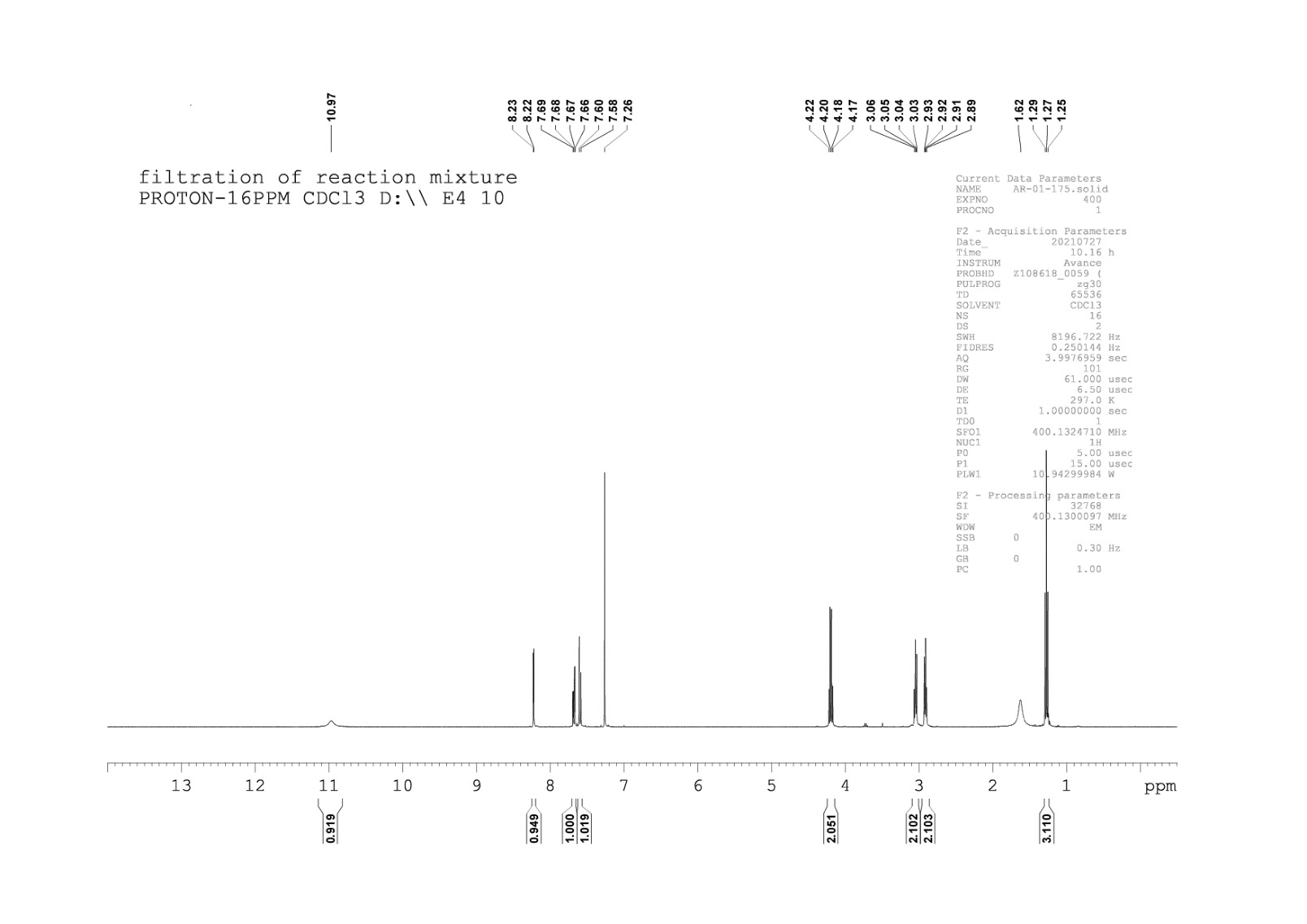


**Fig. S1**. ^1^H NMR (400 MHz, CDCl_3_) spectrum of compound **5b**


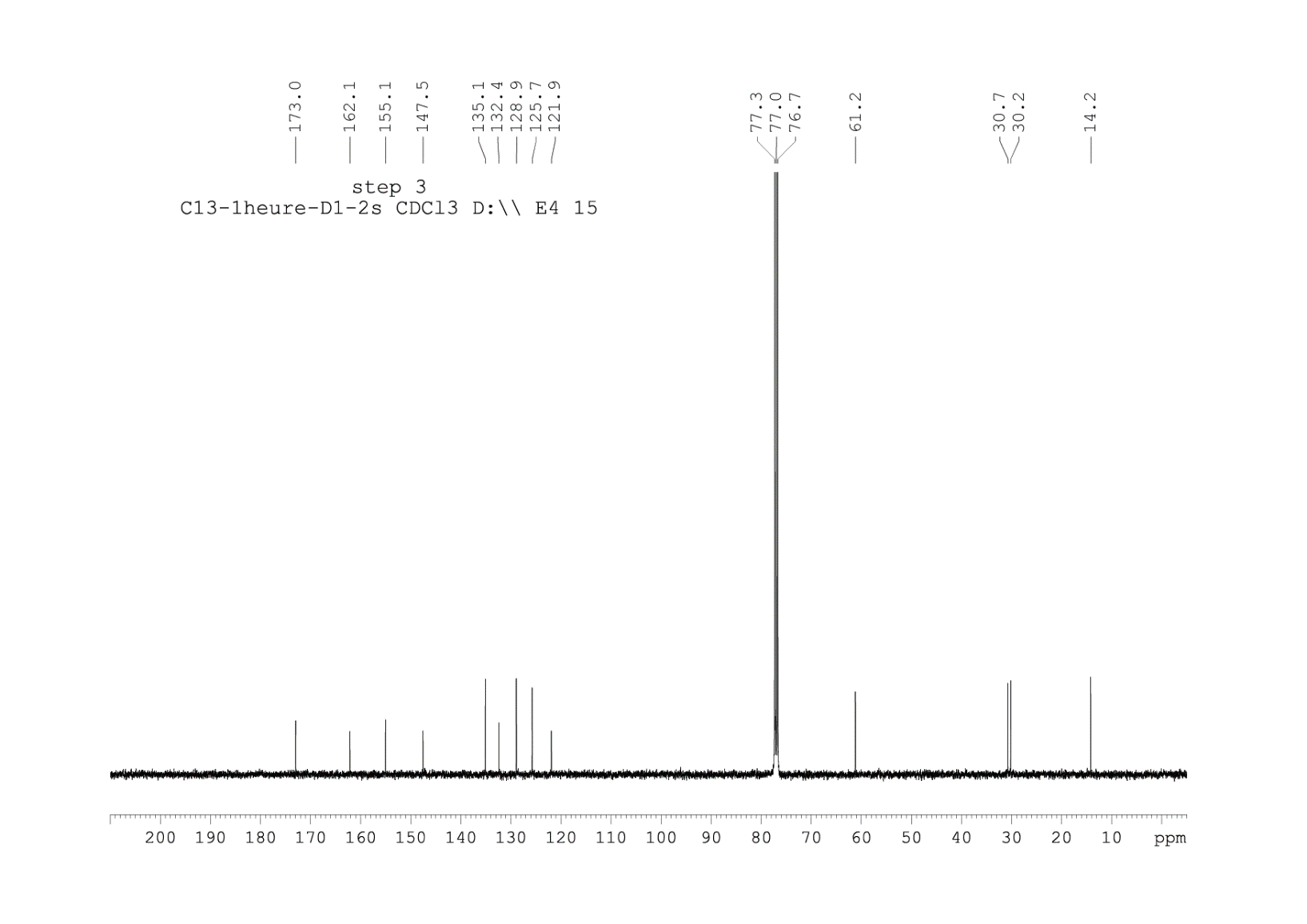


**Fig. S2**. ^13^C NMR (100 MHz, CDCl_3_) spectrum of compound **5b**


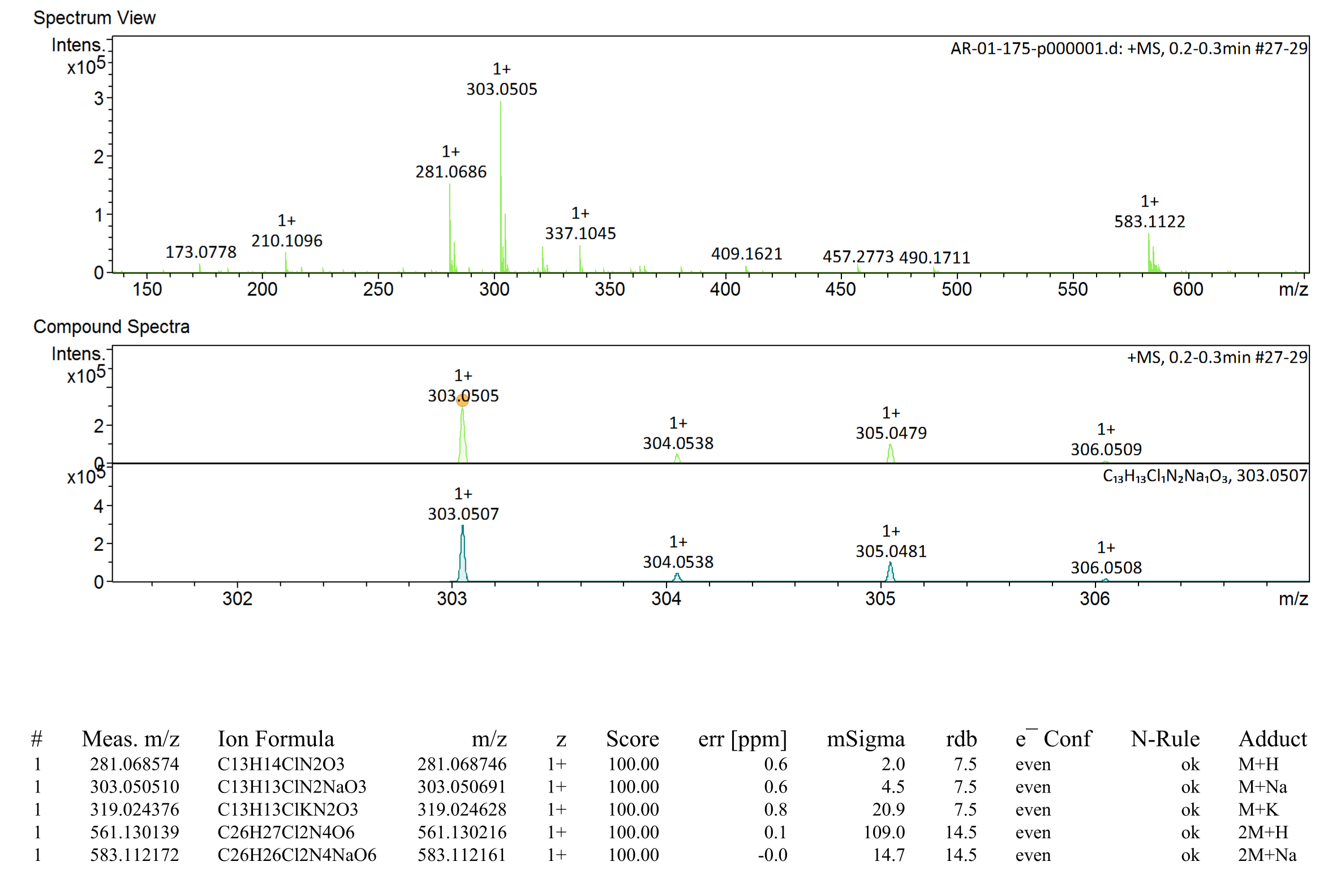


**Fig. S3**. HRMS spectrum of compound **5b**


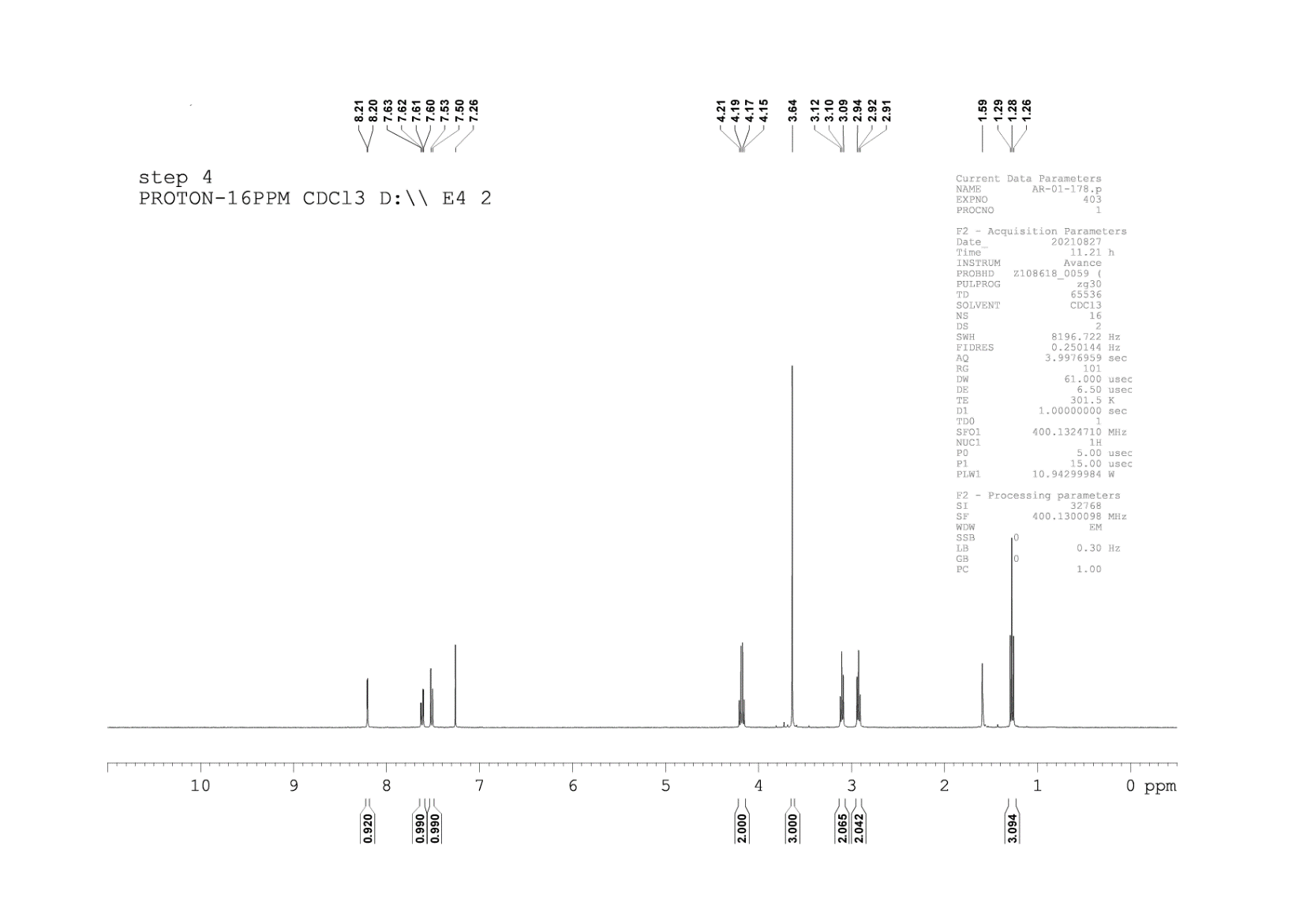


**Fig. S4**. ^1^H NMR (400 MHz, CDCl_3_) spectrum of compound **6b**


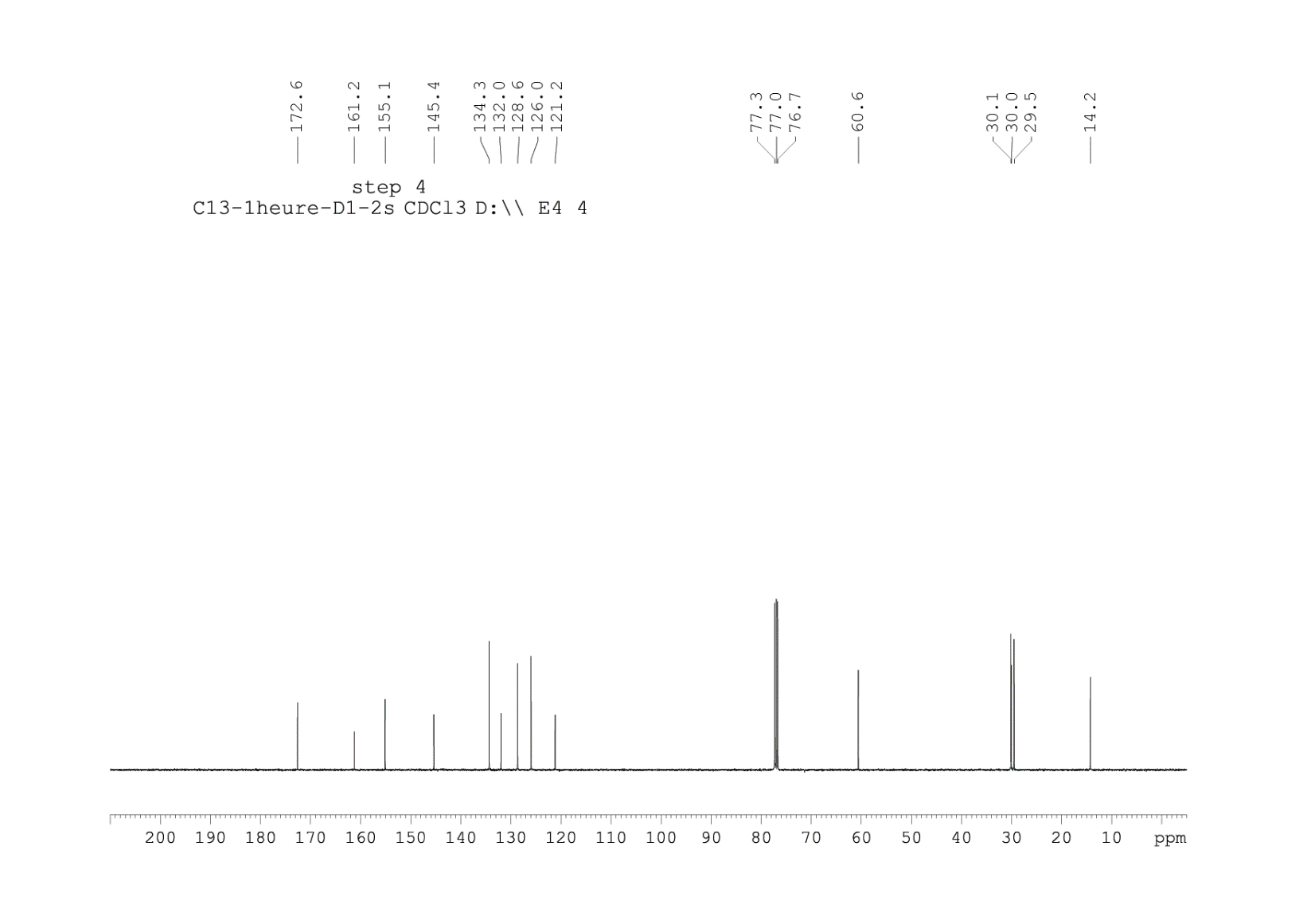


**Fig. S5**. ^13^C NMR (100 MHz, CDCl_3_) spectrum of compound **6b**


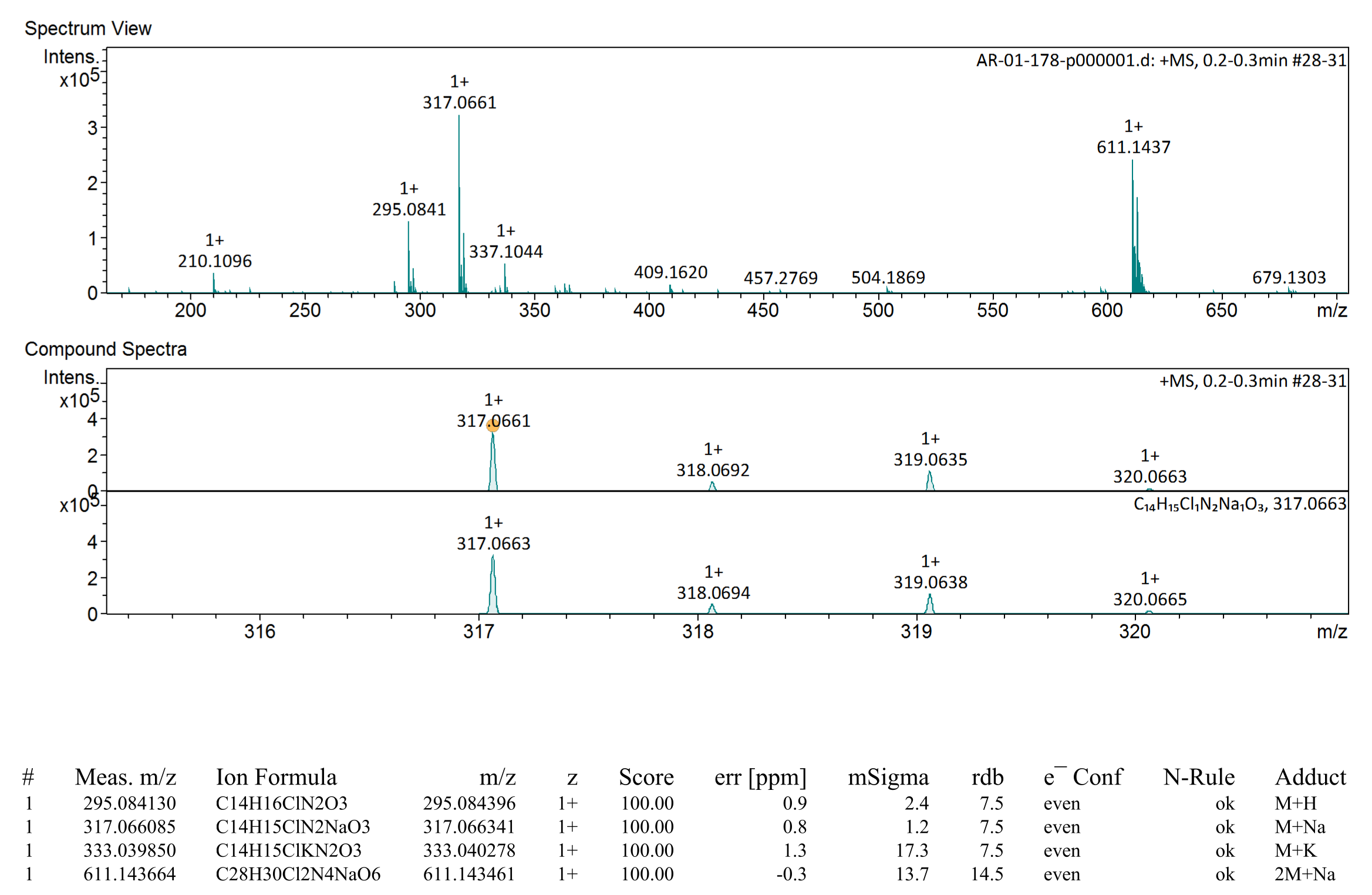


**Fig. S6**. HRMS spectrum of compound **6b**


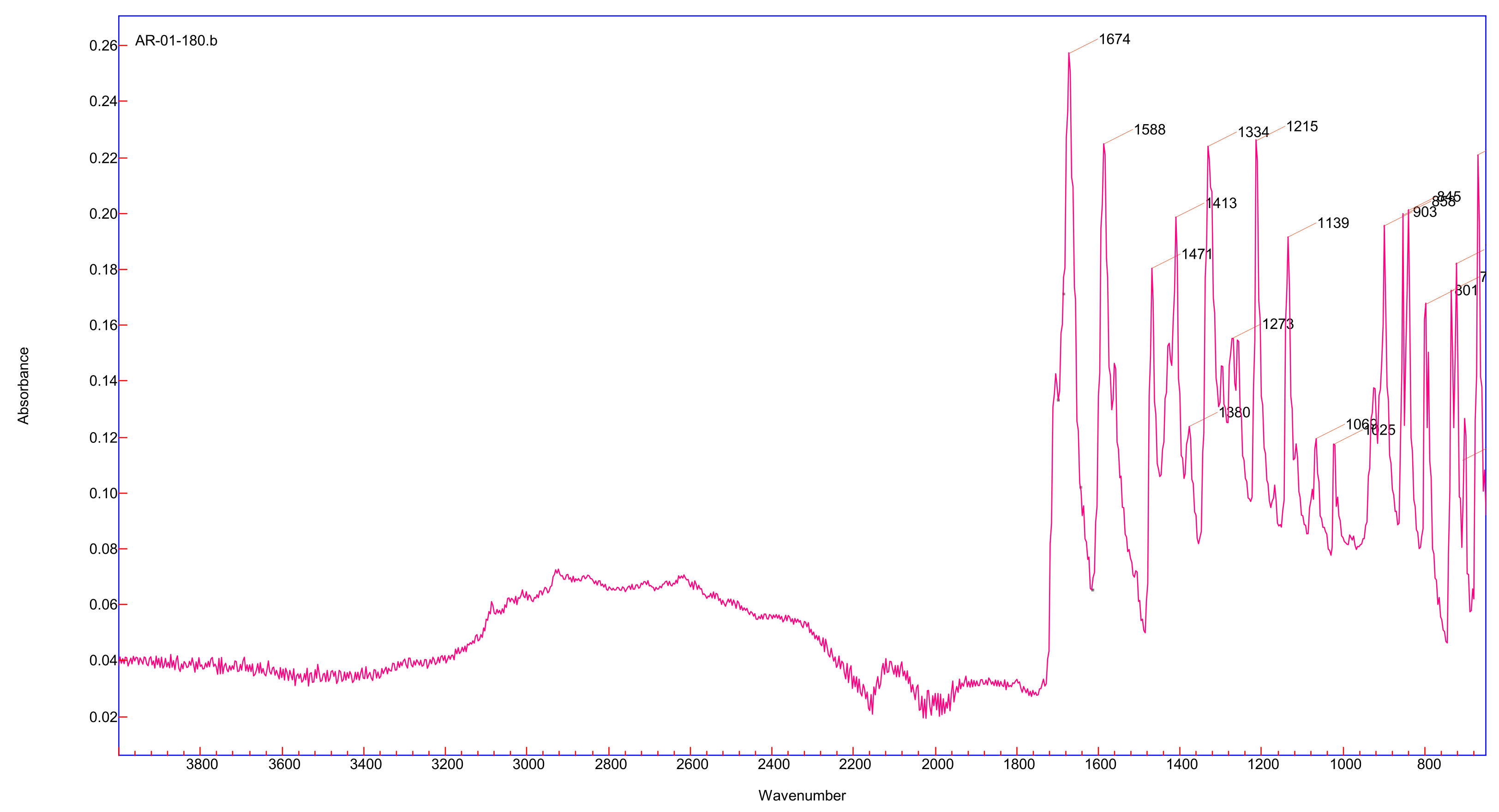


**Fig. S7**. IR spectrum of compound **1b**


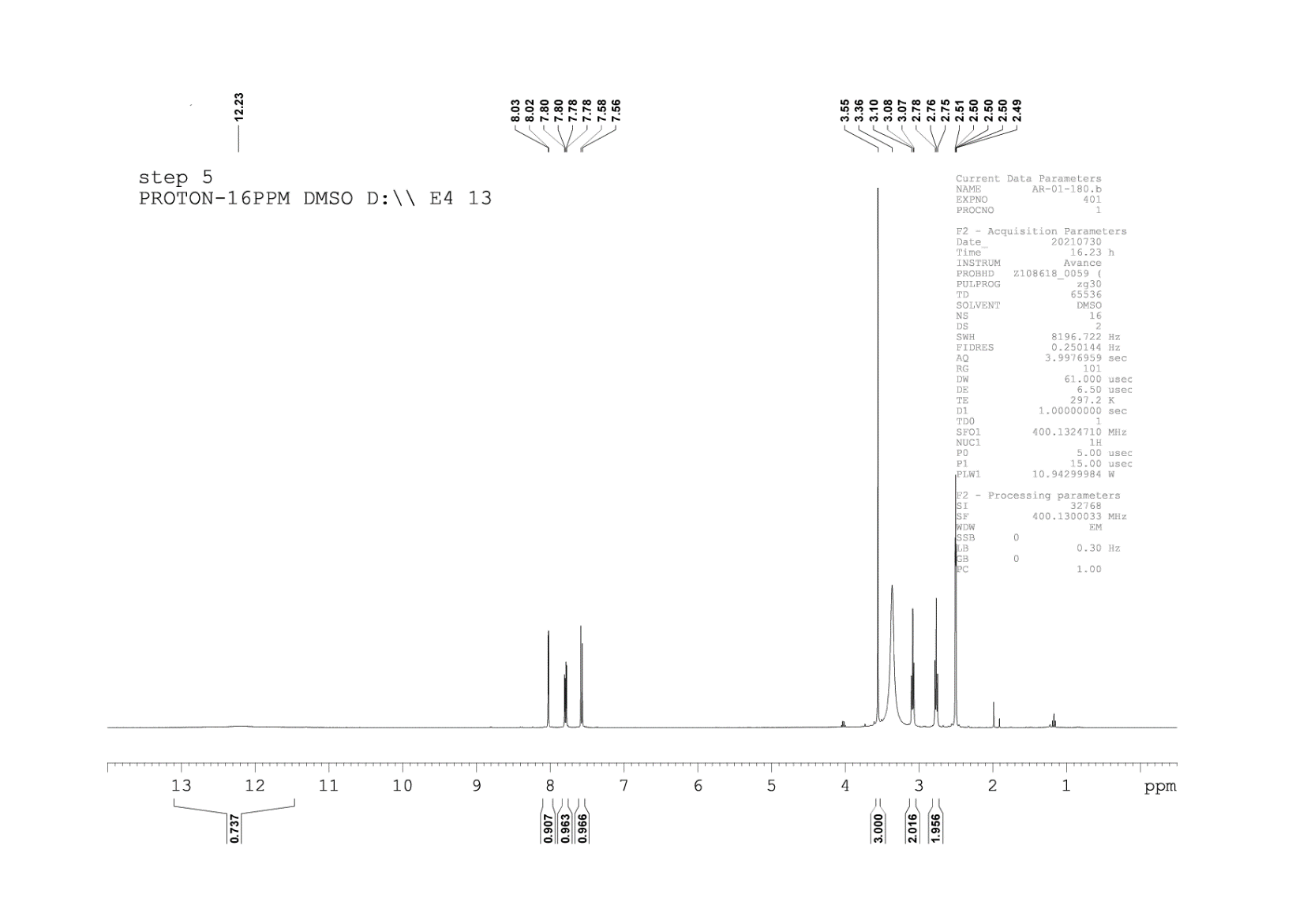


**Fig. S8**. ^1^H NMR (400 MHz, DMSO-*d_6_*) spectrum of compound **1b**


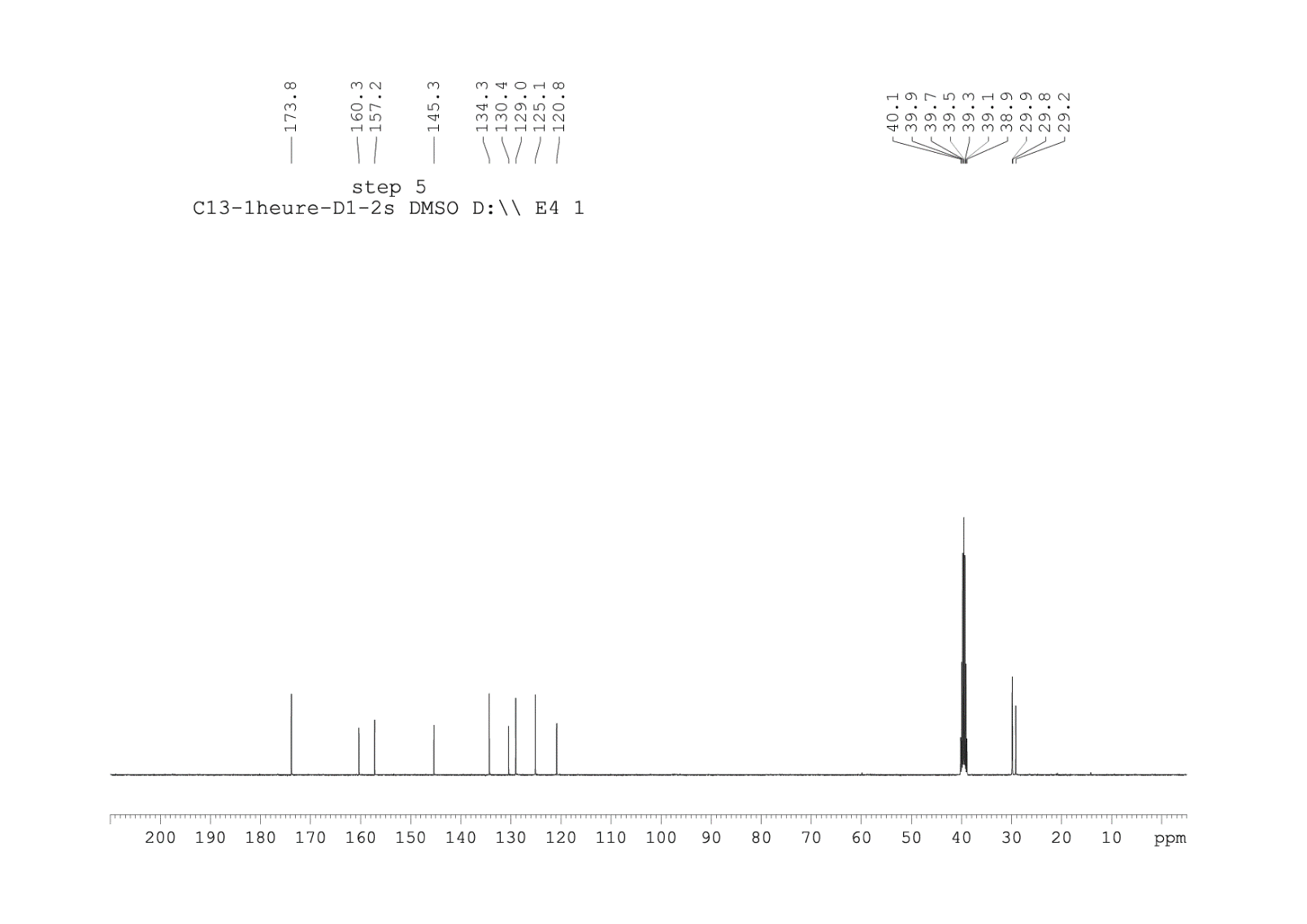


**Fig. S9**. ^13^C NMR (100 MHz, DMSO-*d_6_*) spectrum of compound **1b**


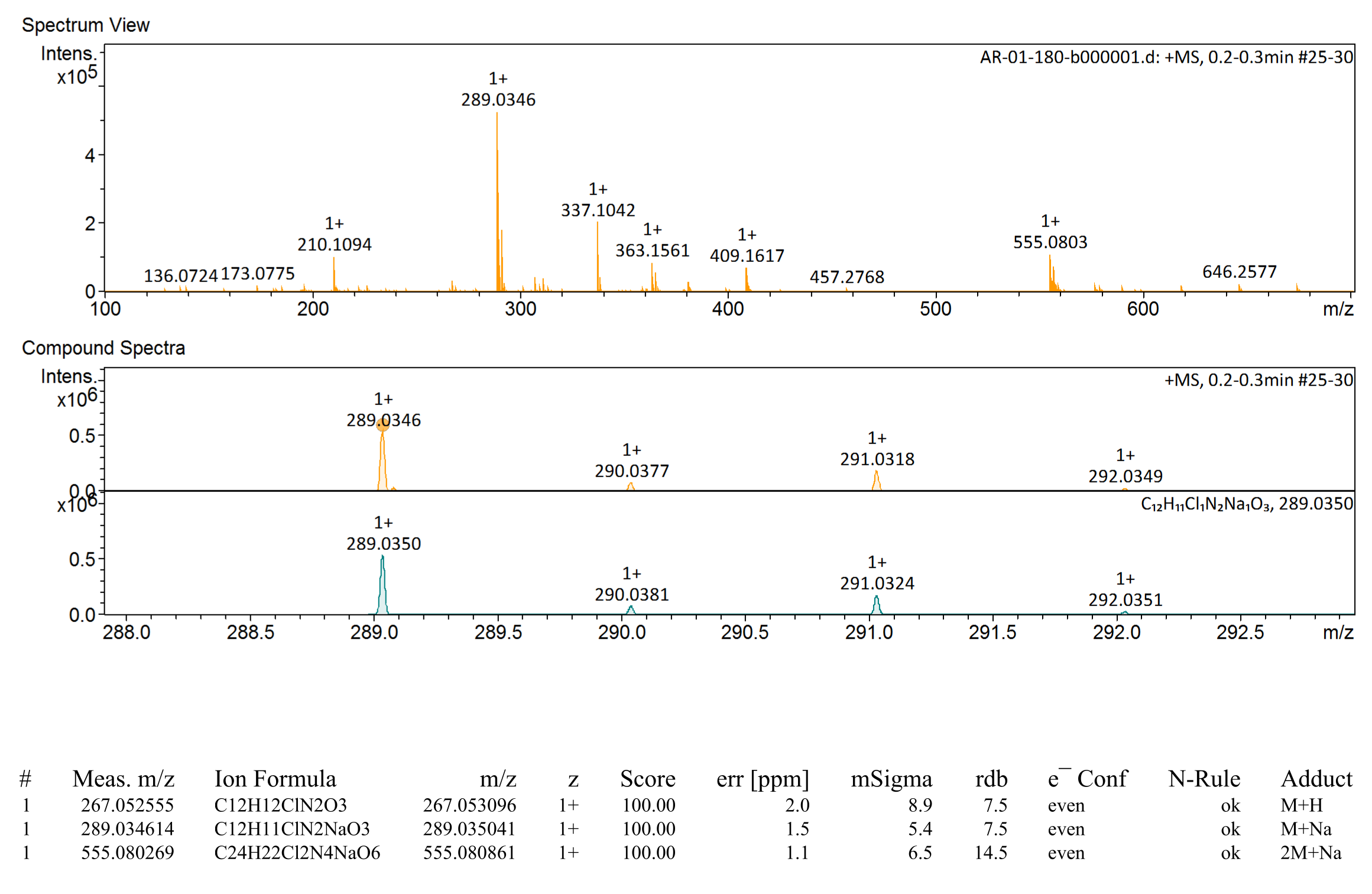


**Fig. S10**. HRMS spectrum of compound **1b**


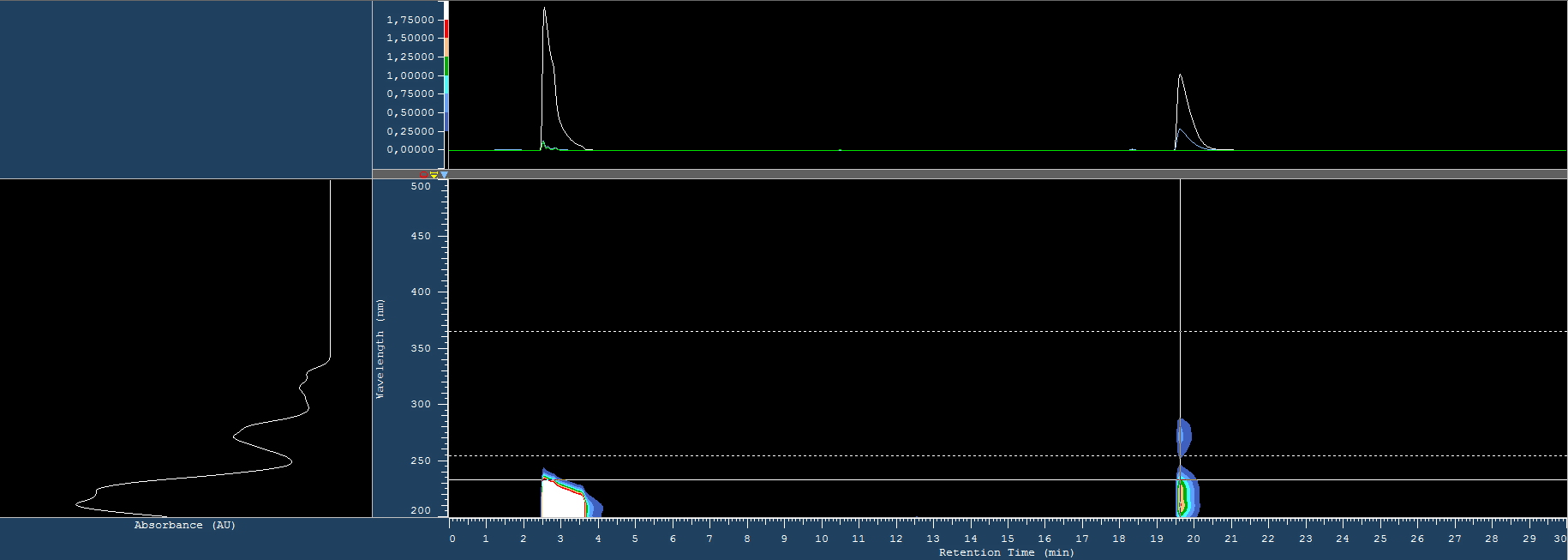


**Fig. S11**. HPLC-UV spectrum of compound **1b**


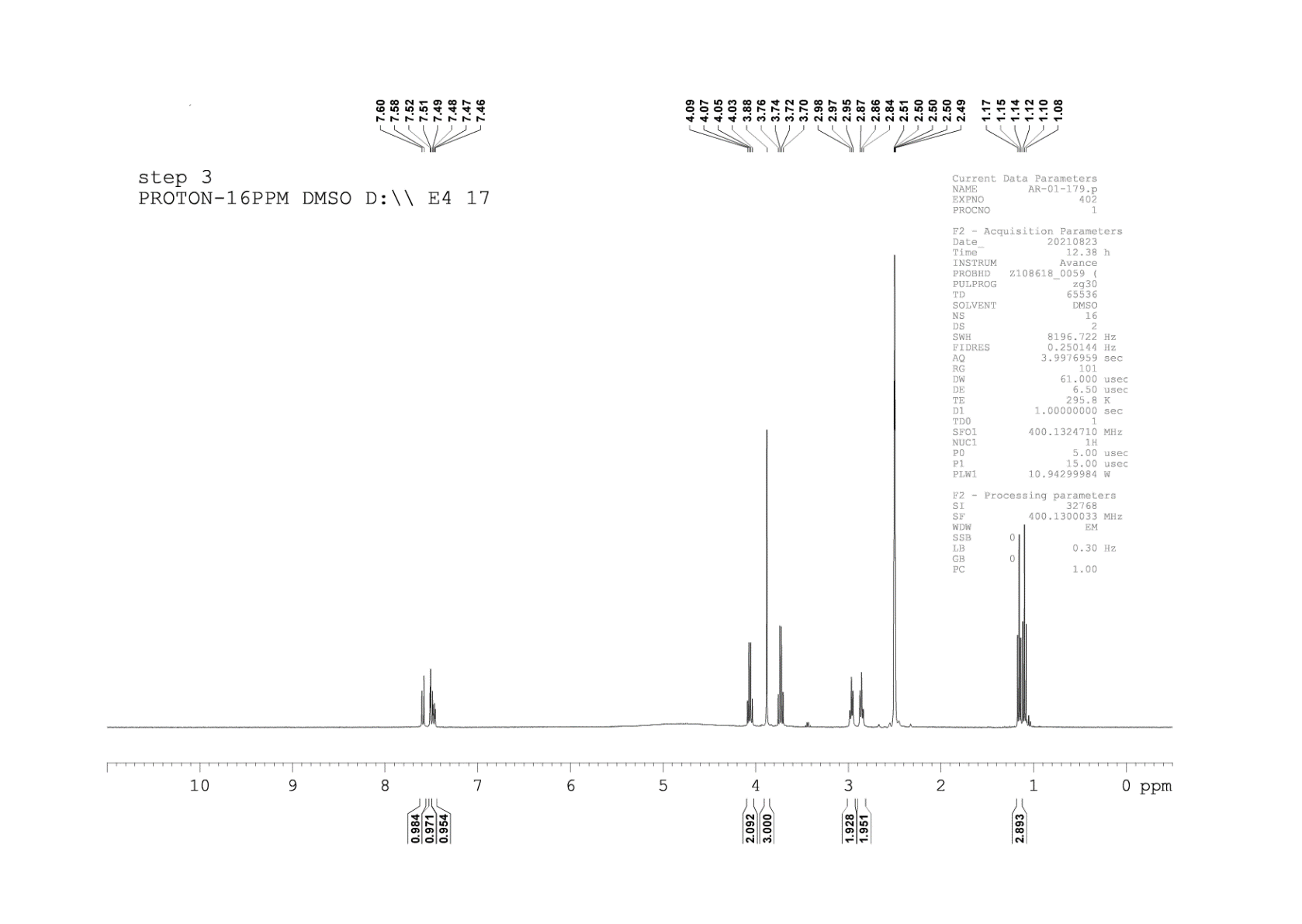


**Fig. S12**. ^1^H NMR (400 MHz, DMSO-*d_6_*) spectrum of compound **5c**


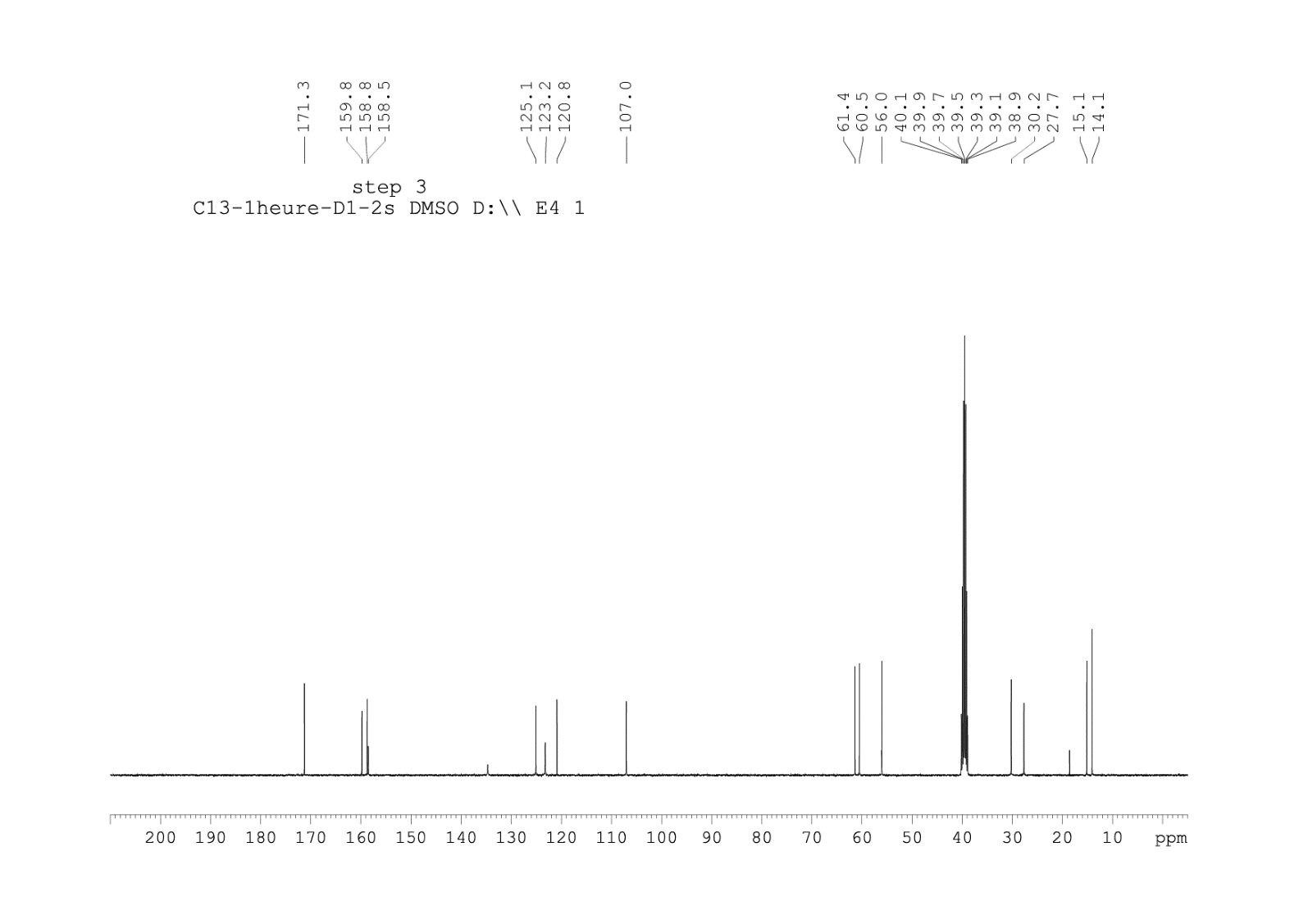


**Fig. S13**. ^13^C NMR (100 MHz, DMSO-*d_6_*) spectrum of compound **5c**


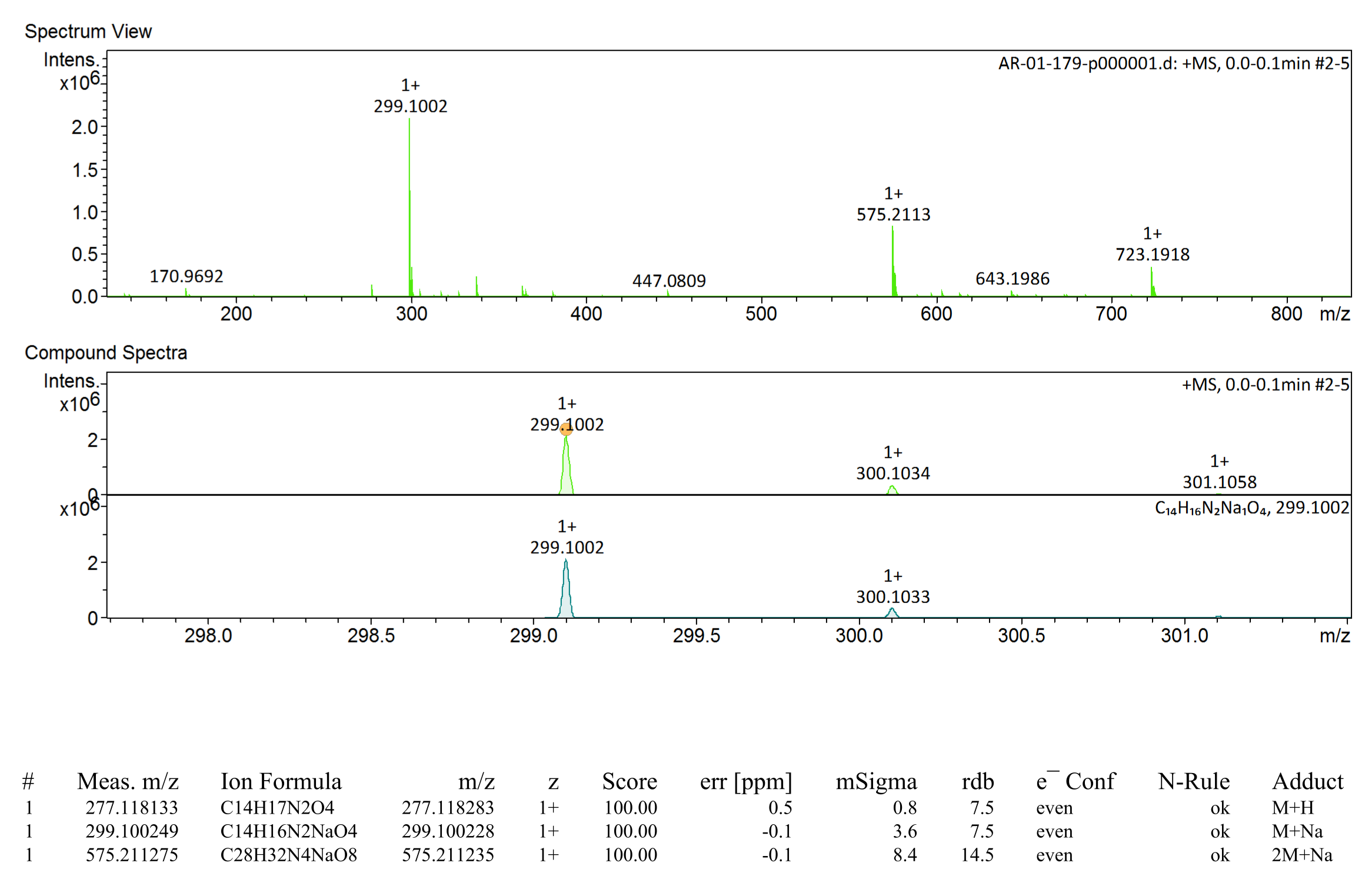


**Fig. S14**. HRMS spectrum of compound **5c**

**
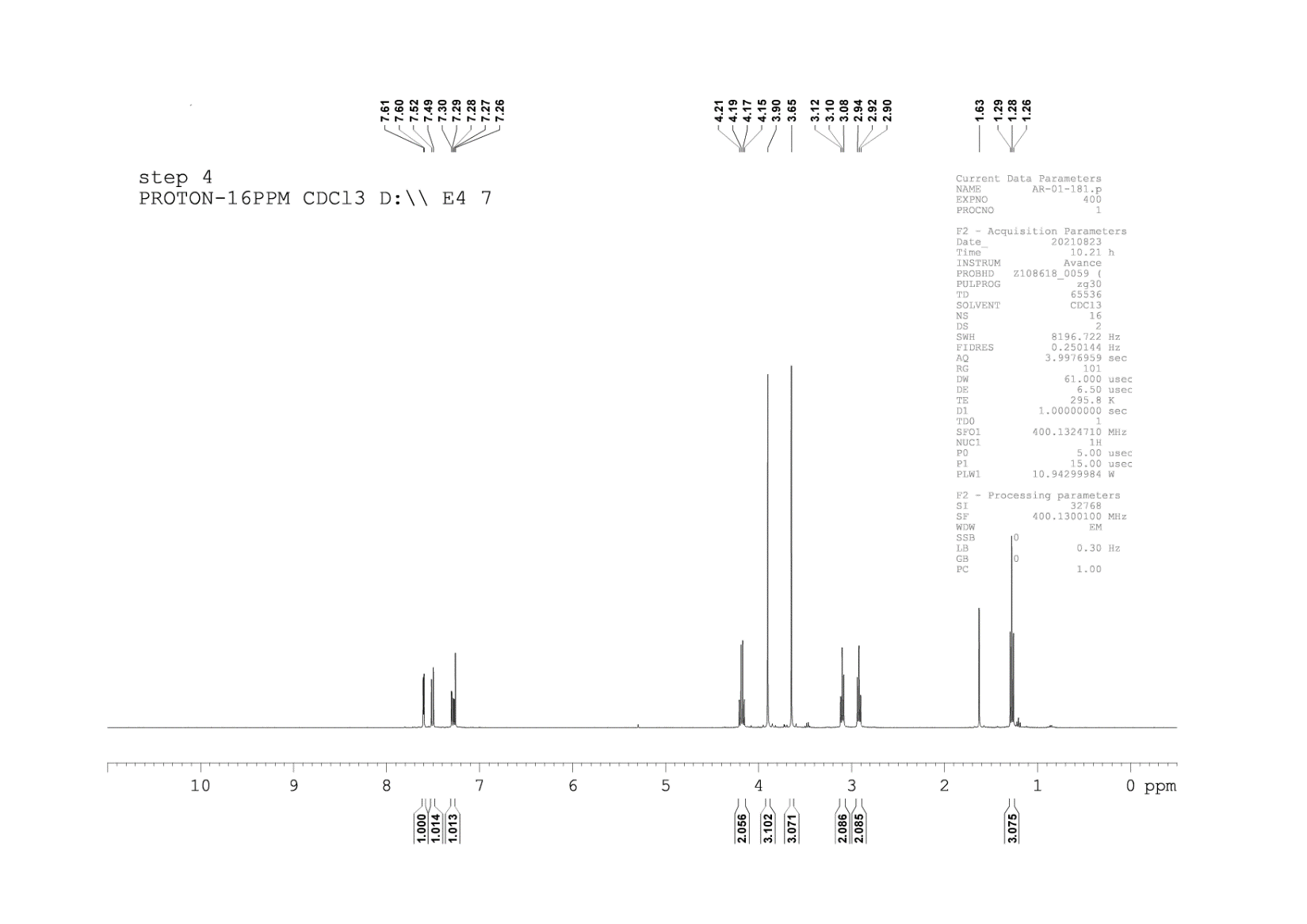
**

**Fig. S15**. ^1^H NMR (400 MHz, CDCl_3_) spectrum of compound **6c**


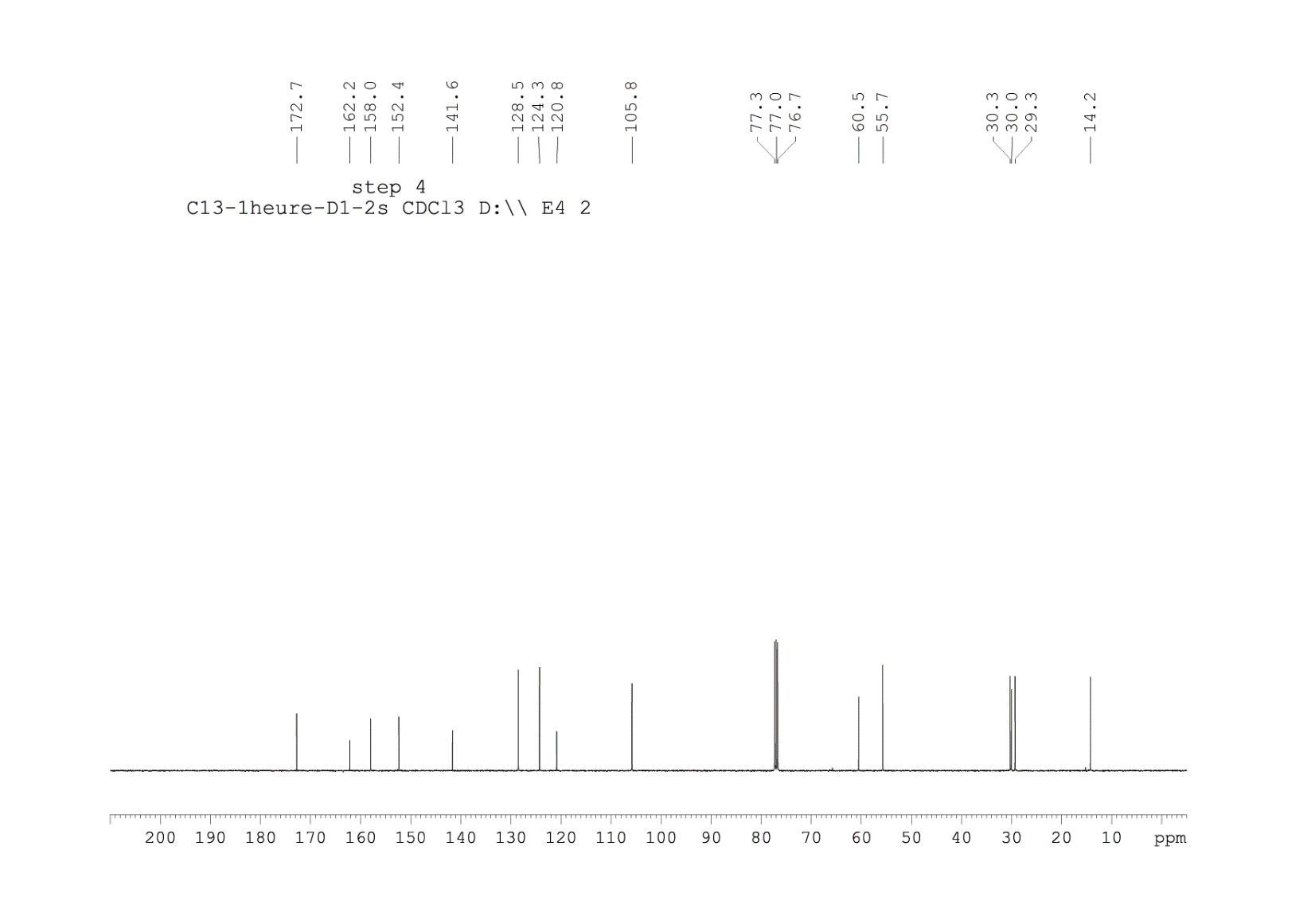


**Fig. S16**. ^13^C NMR (100 MHz, CDCl_3_) spectrum of compound **6c**


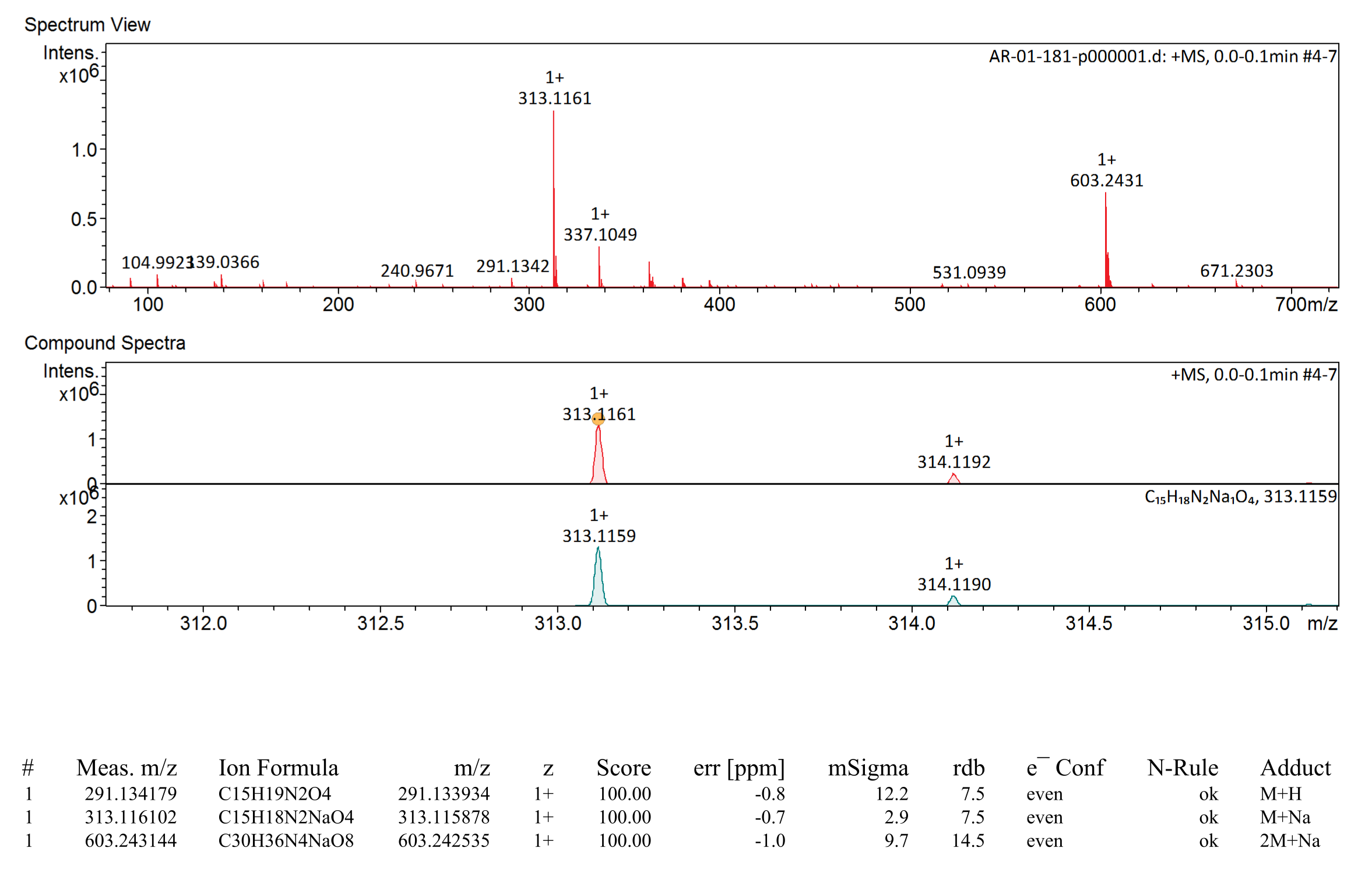


**Fig. S17**. HRMS spectrum of compound **6c**


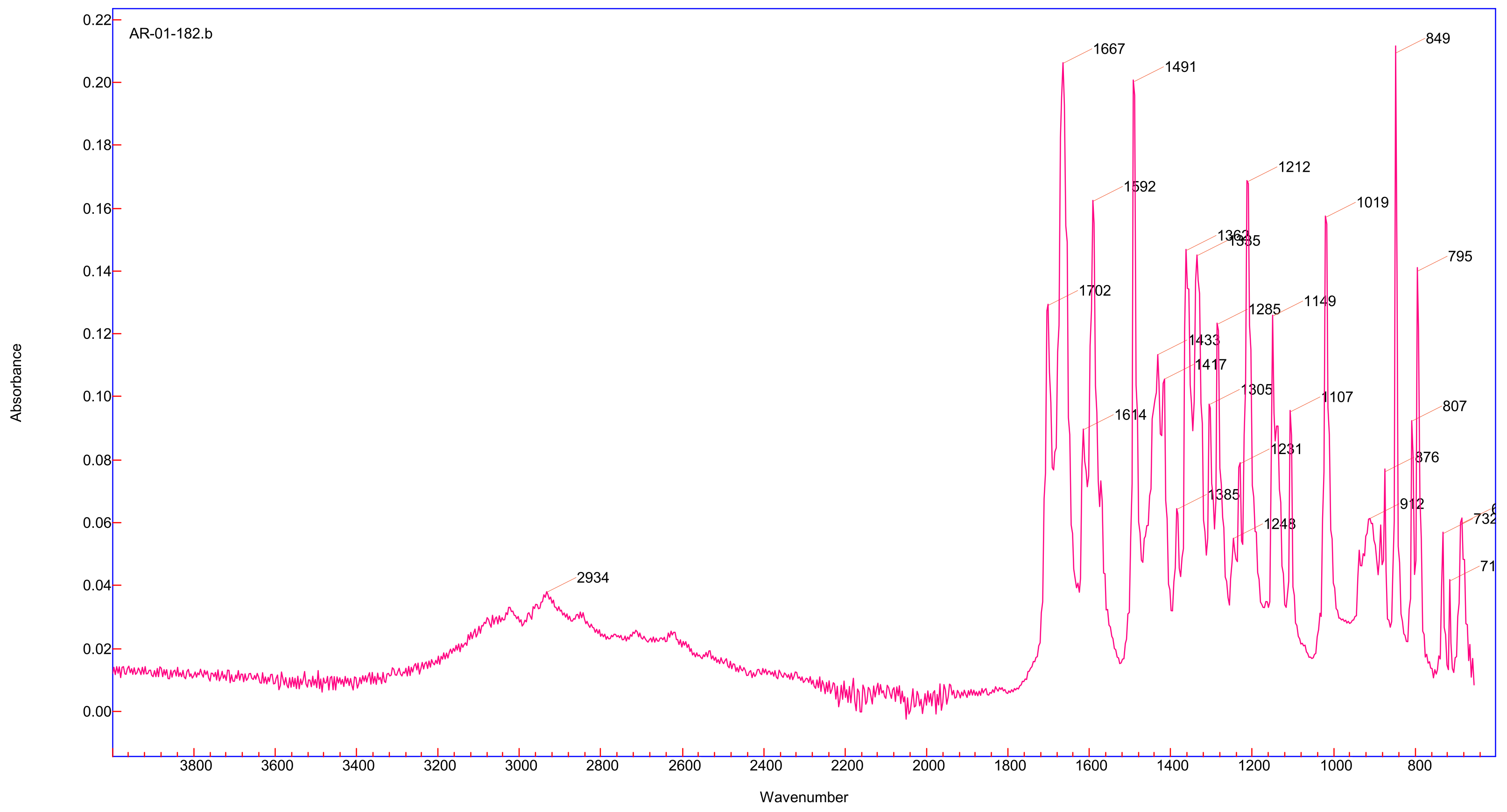


**Fig. S18**. IR spectrum of compound **1c**


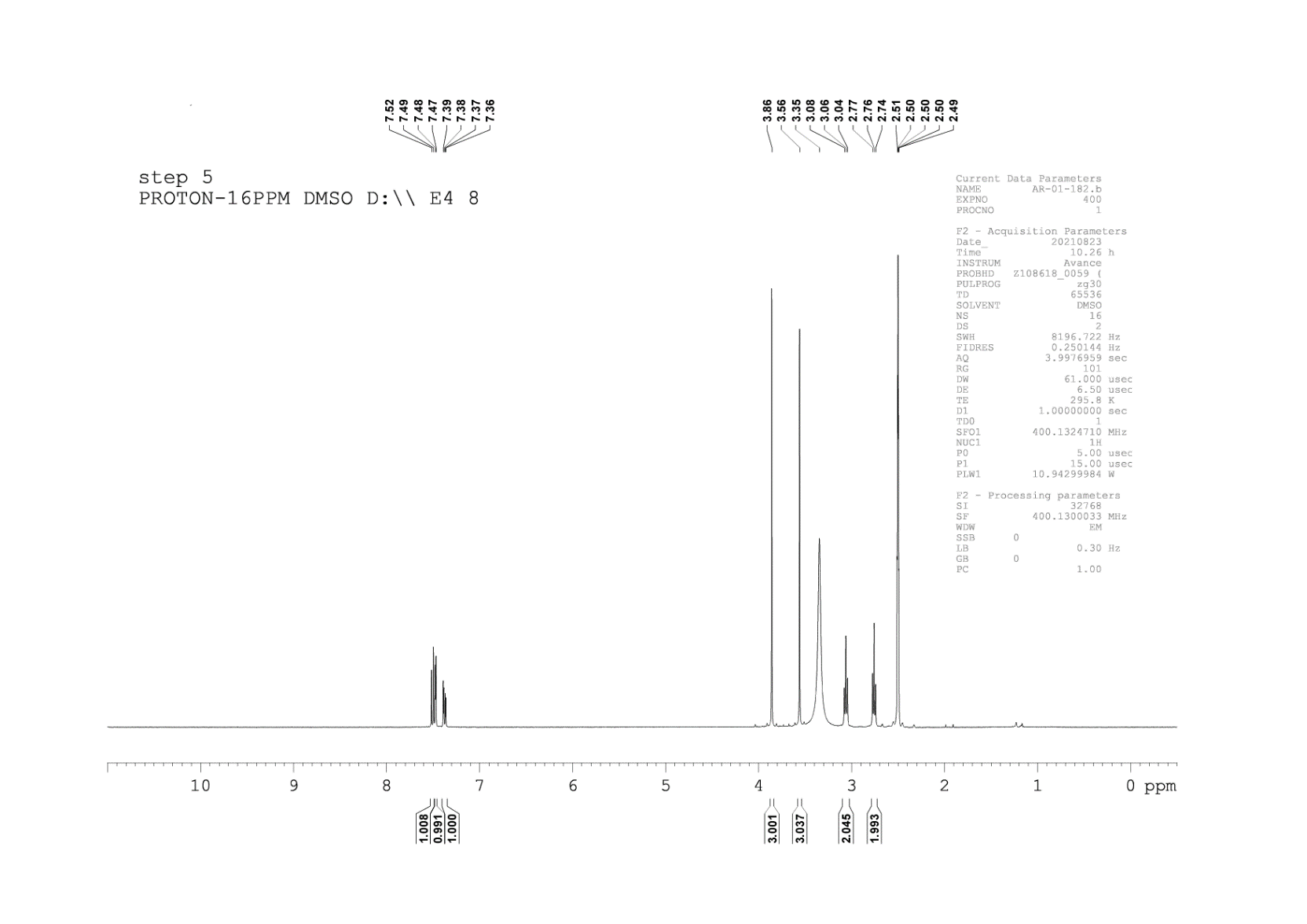


**Fig. S19**. ^1^H NMR (400 MHz, DMSO-*d_6_*) spectrum of compound **1c**


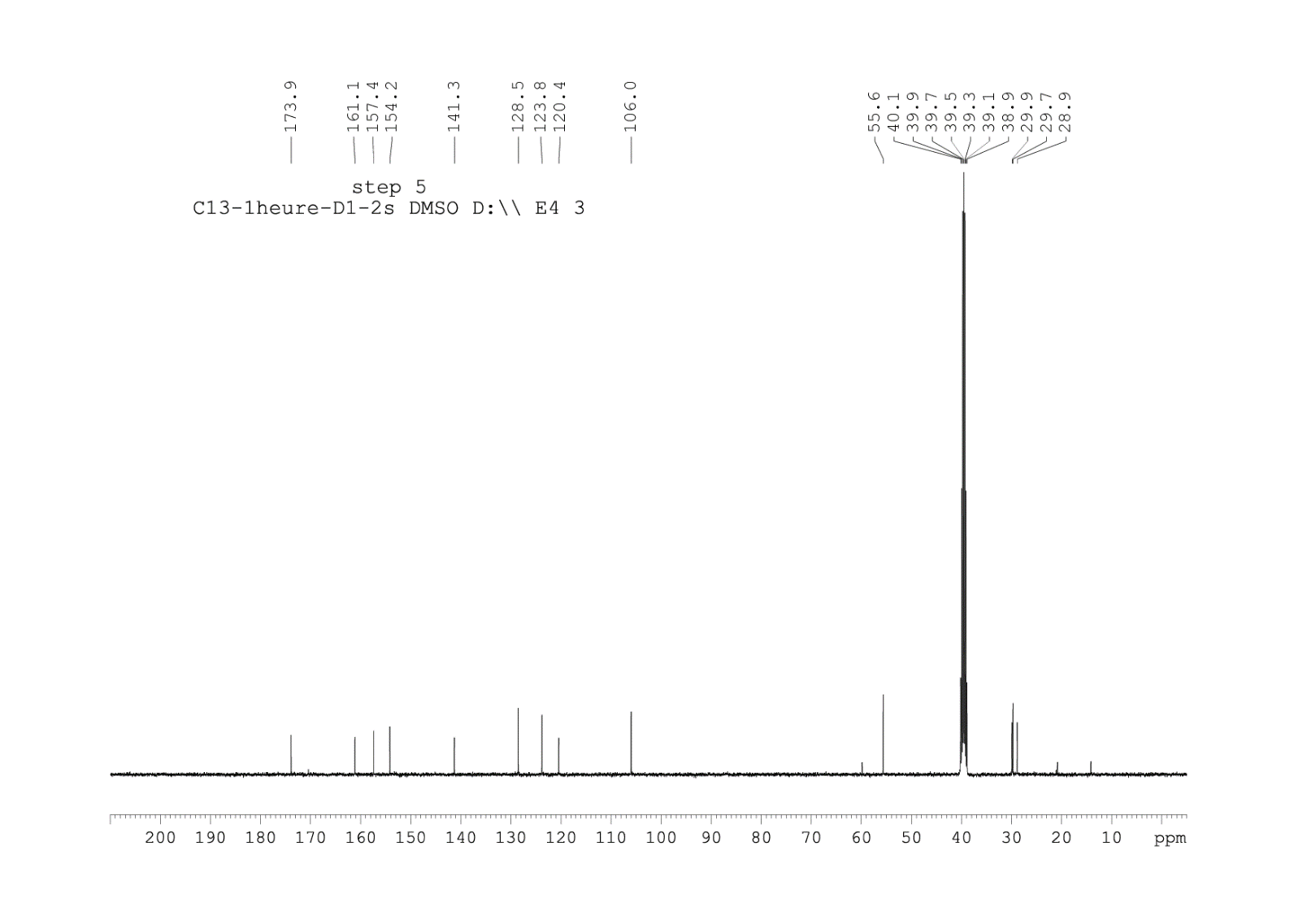


**Fig. S20**. ^13^C NMR (100 MHz, DMSO-*d_6_*) spectrum of compound **1c**


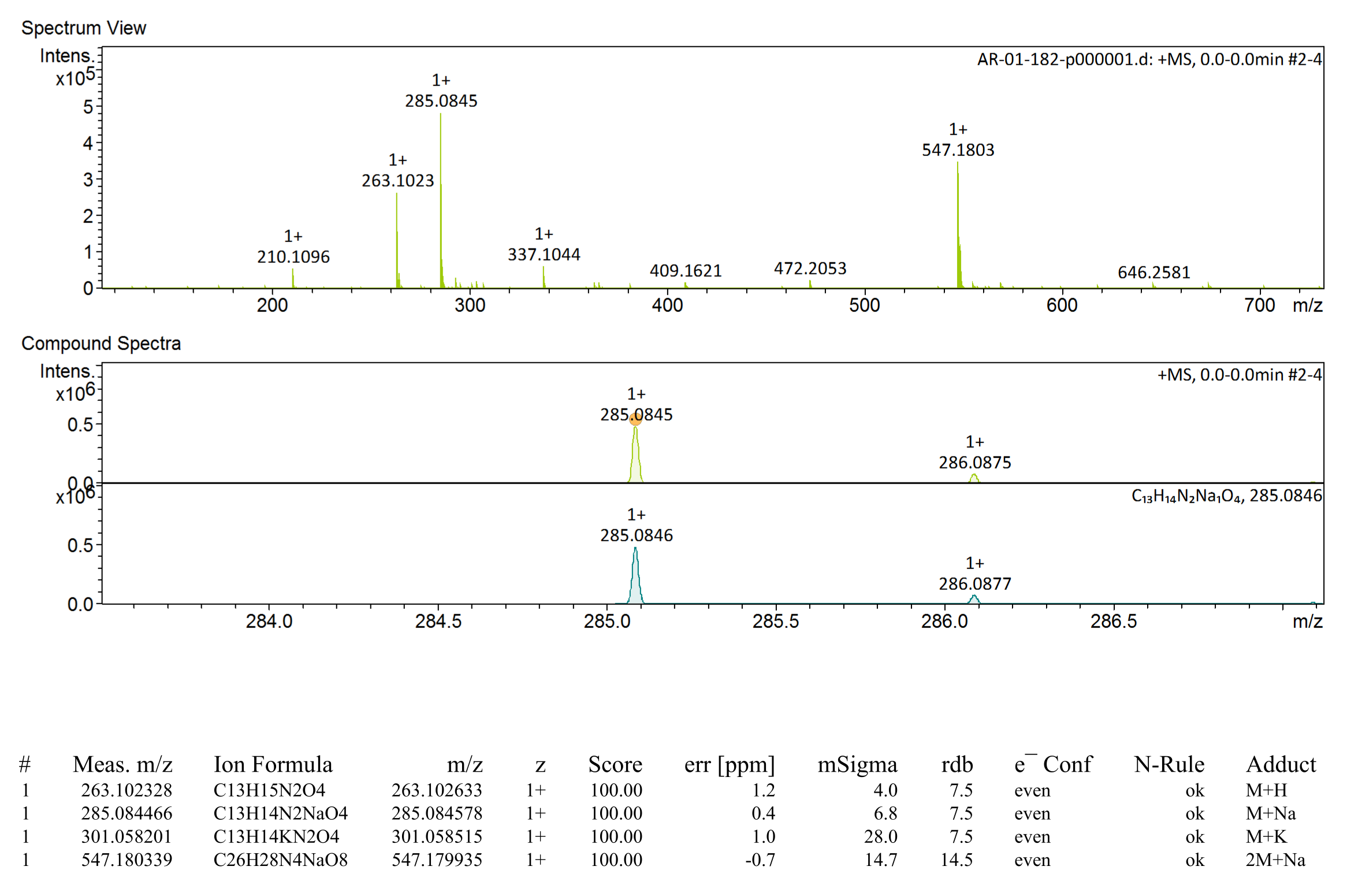


**Fig. S21**. HRMS spectrum of compound **1c**


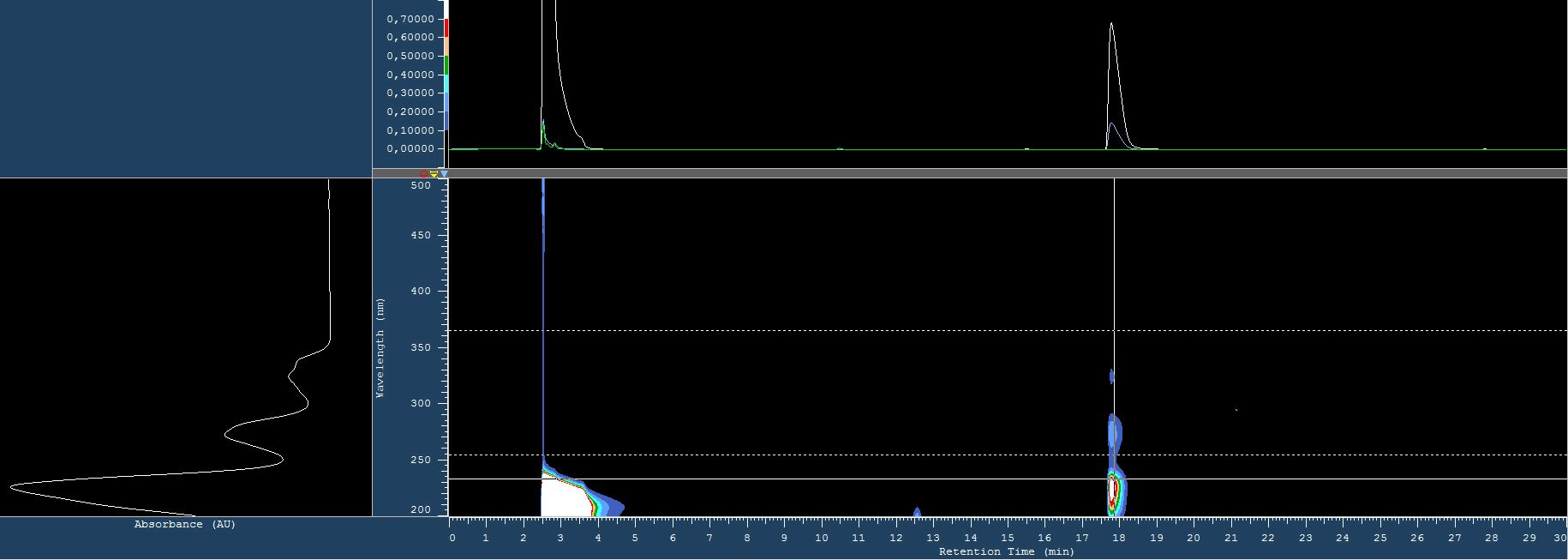


**Fig. S22**. HPLC-UV spectrum of compound **1c**


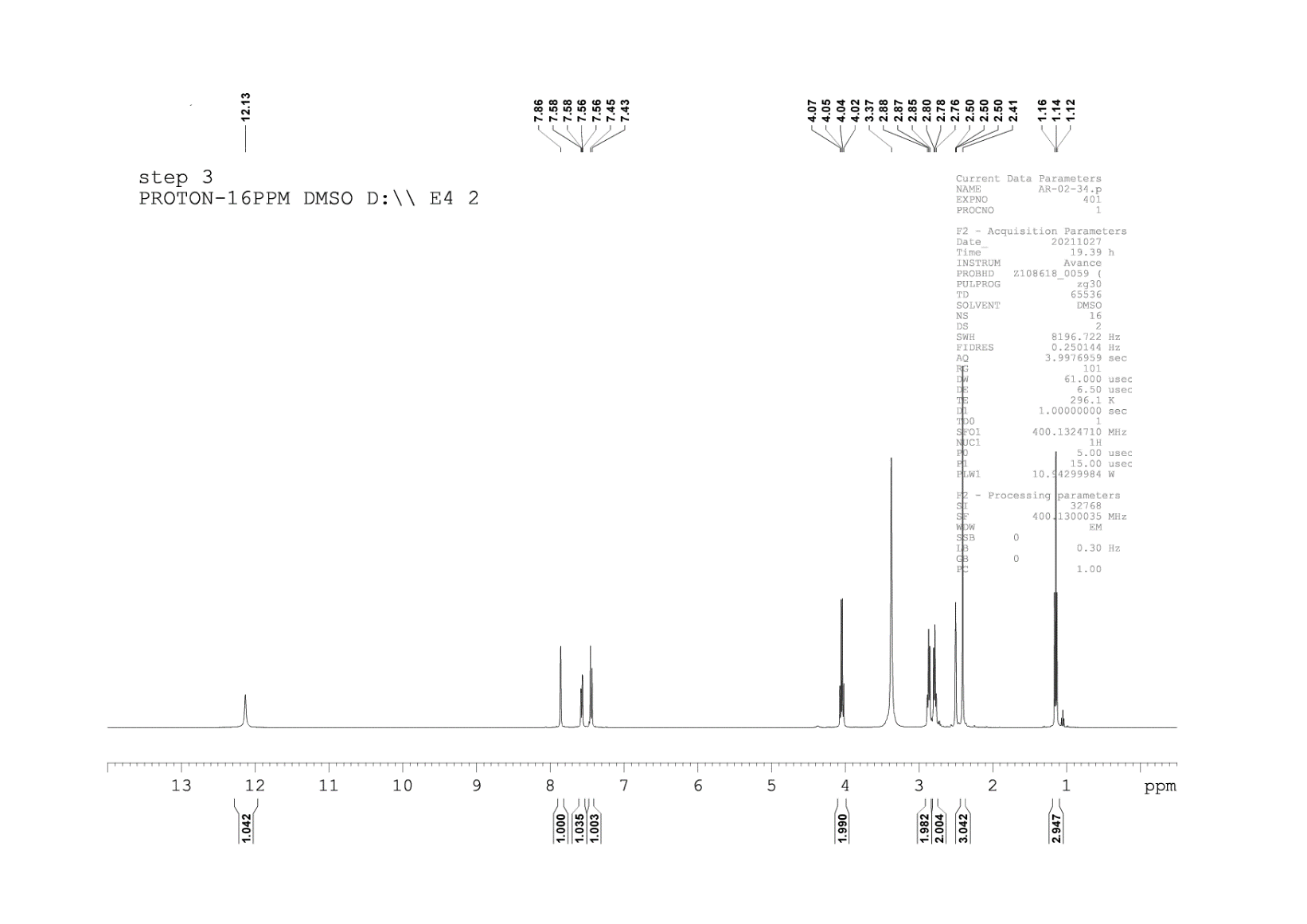


**Fig. S23**. ^1^H NMR (400 MHz, DMSO-*d_6_*) spectrum of compound **5d**


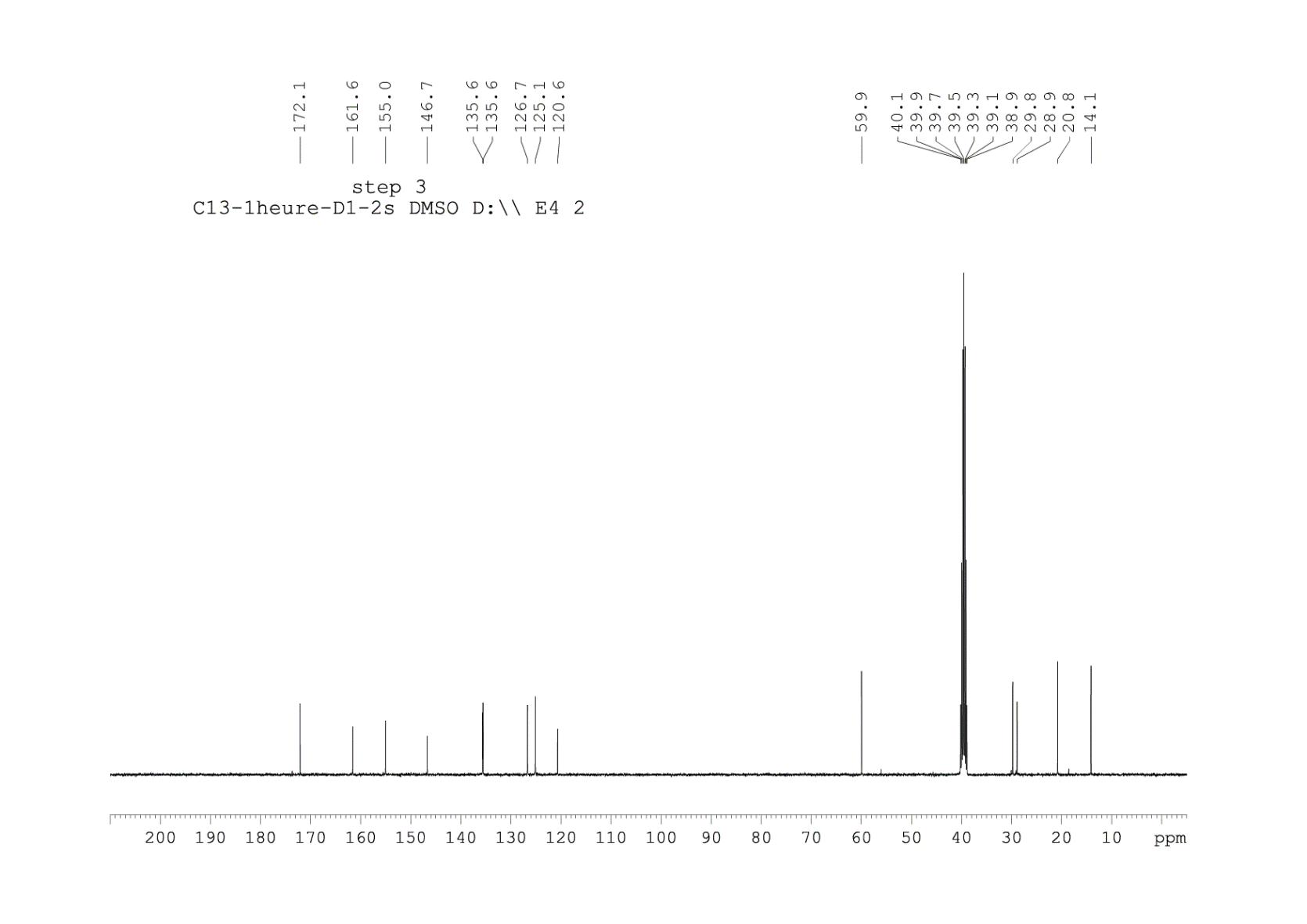


**Fig. S24**. ^13^C NMR (100 MHz, DMSO-*d_6_*) spectrum of compound **5d**


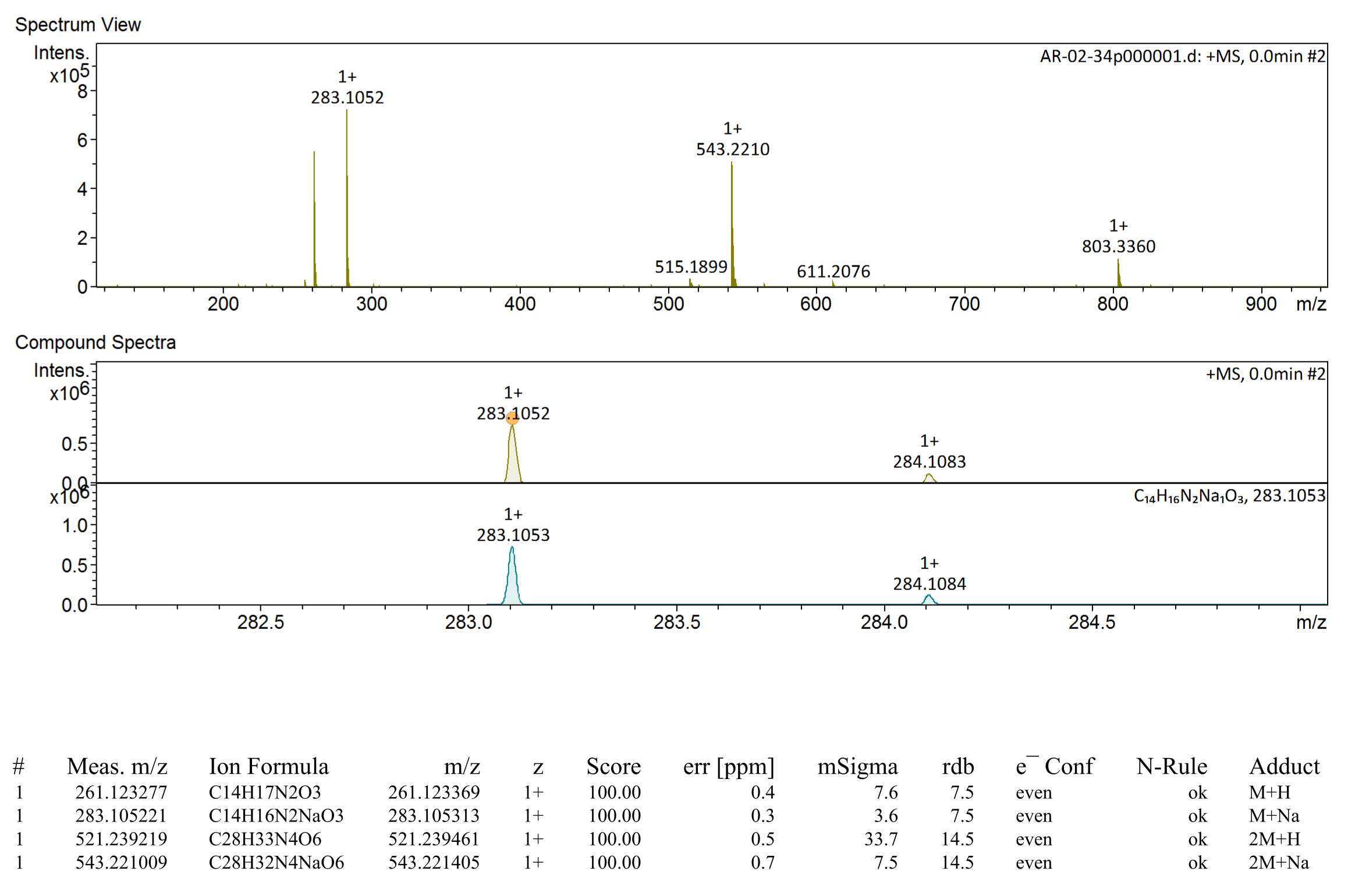


**Fig. S25**. HRMS spectrum of compound **5d**


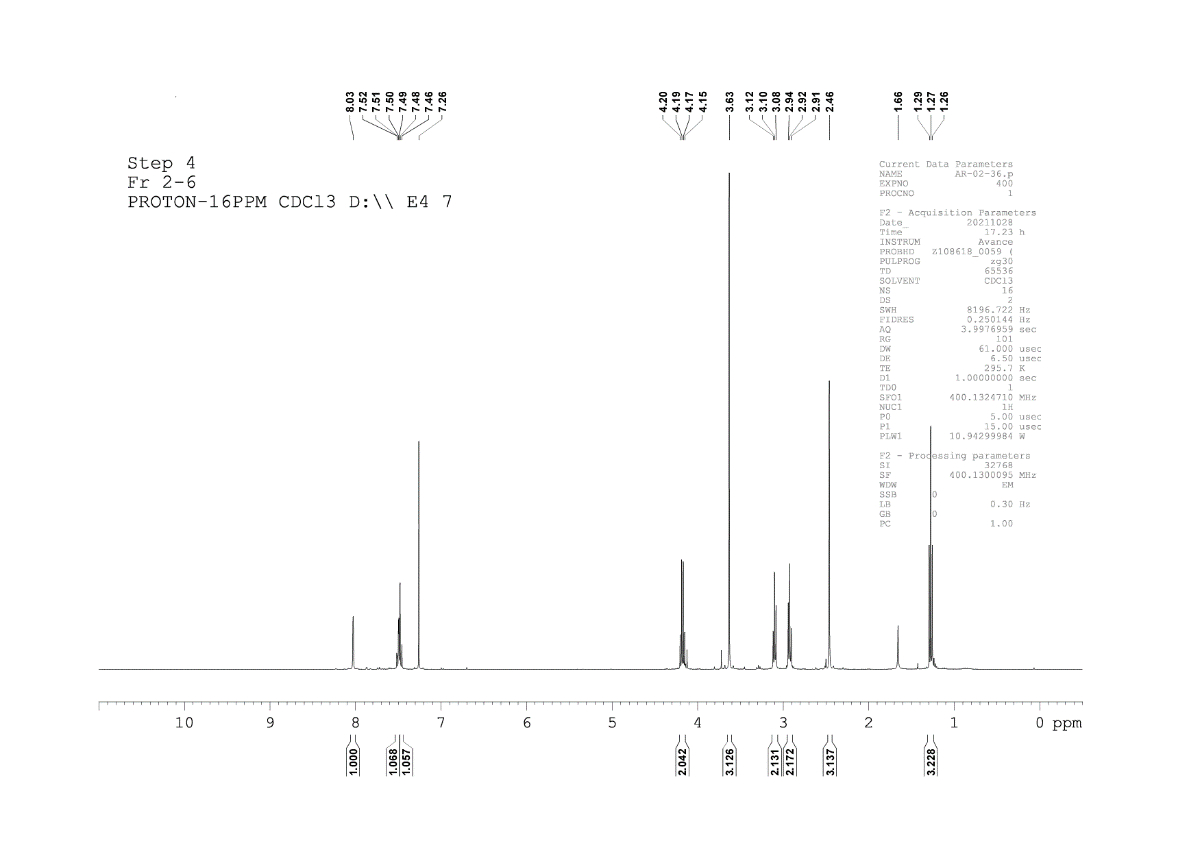


**Fig. S26**. ^1^H NMR (400 MHz, CDCl_3_) spectrum of compound **6d**


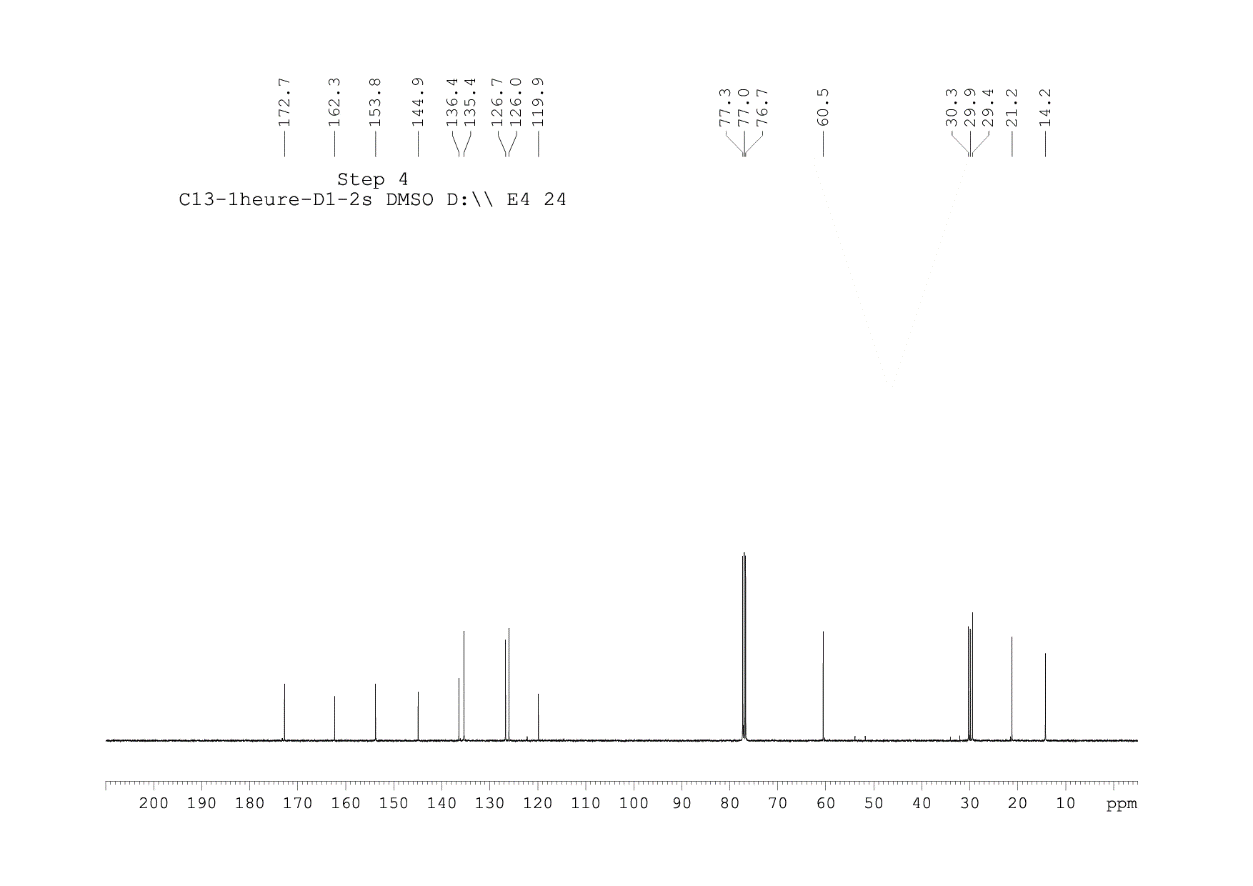


**Fig. S27**. ^13^C NMR (100 MHz, CDCl_3_) spectrum of compound **6d**


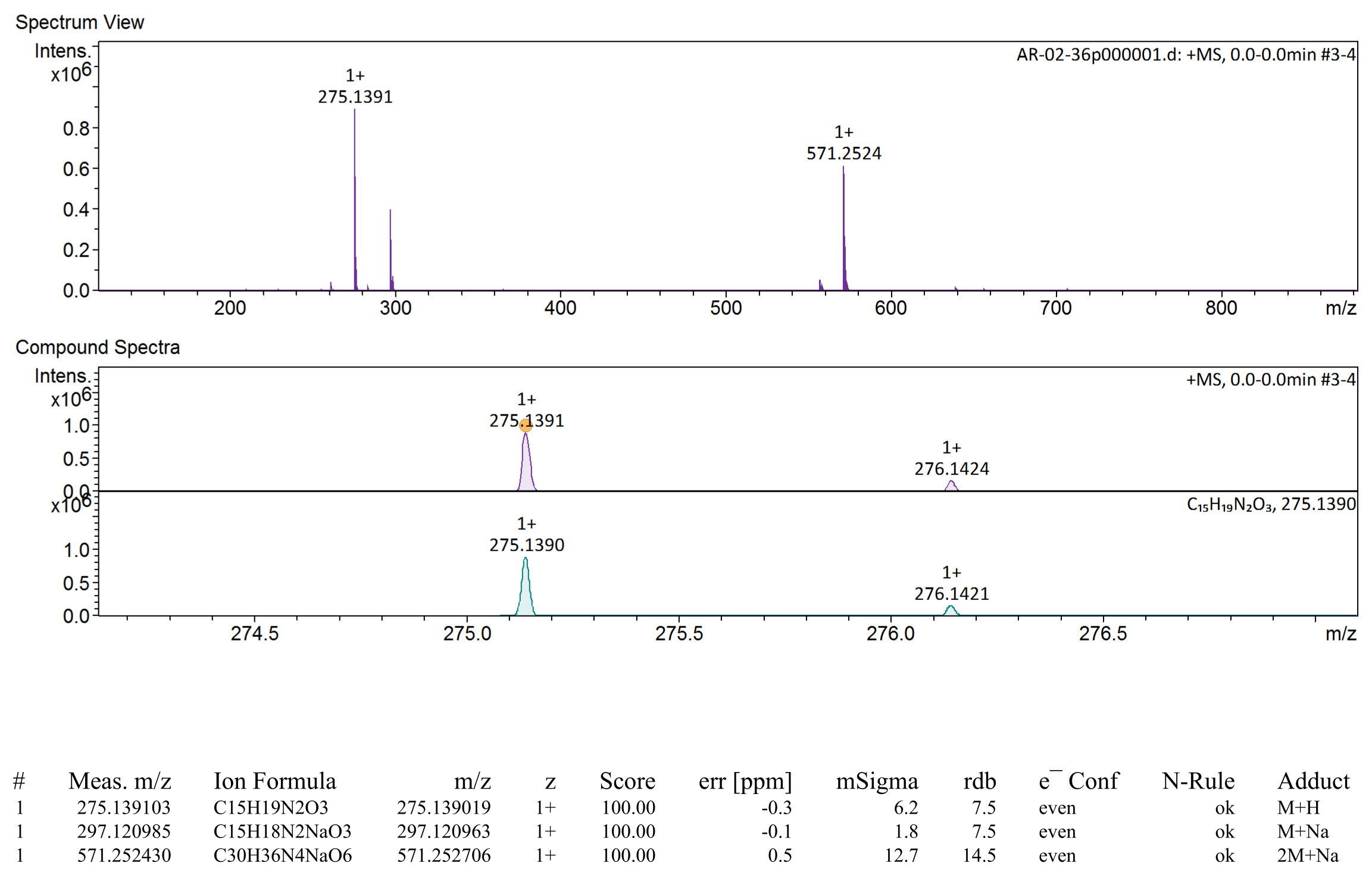


**Fig. S28**. HRMS spectrum of compound **6d**


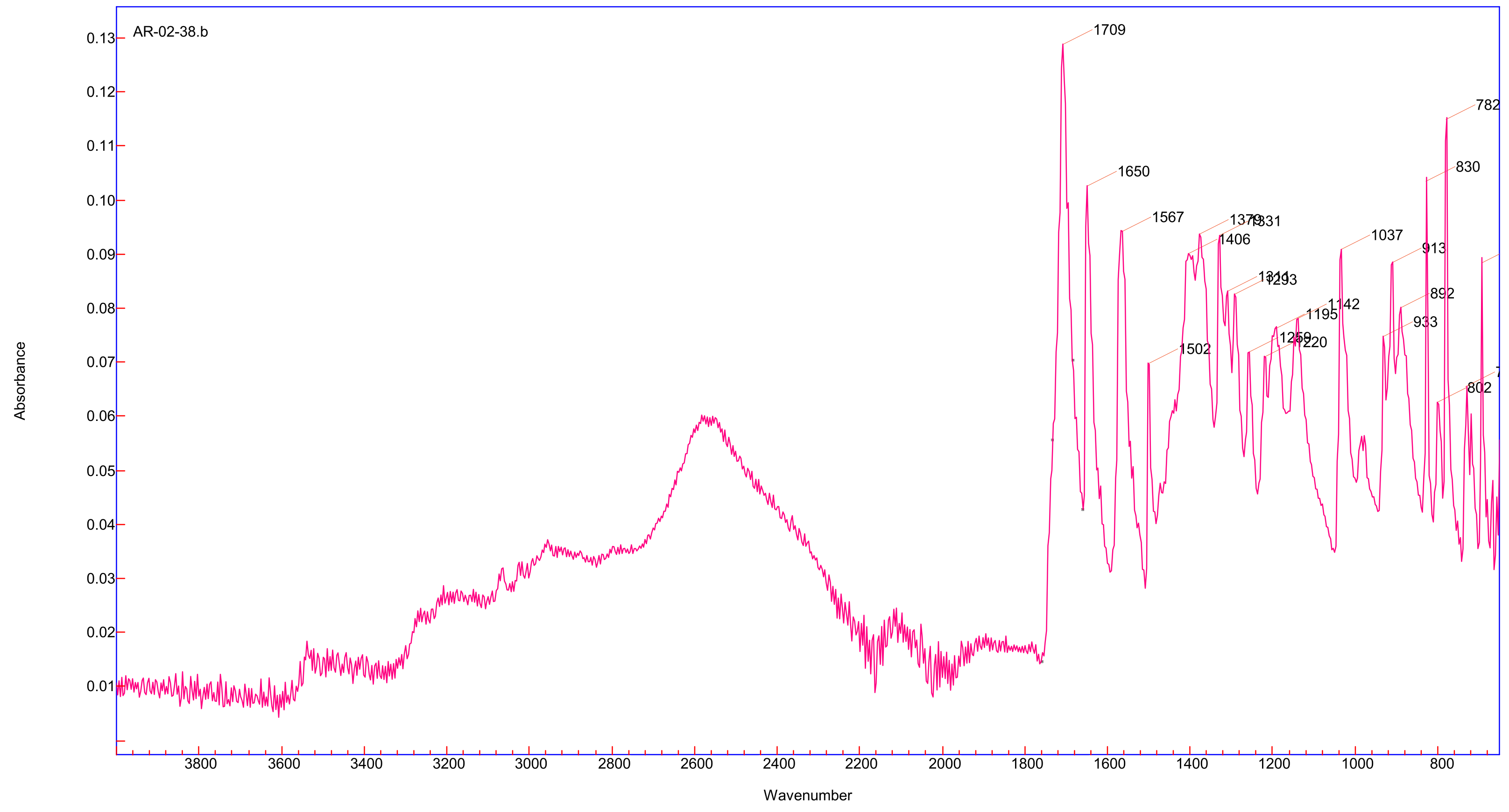


**Fig. S29**. IR spectrum of compound **1d**


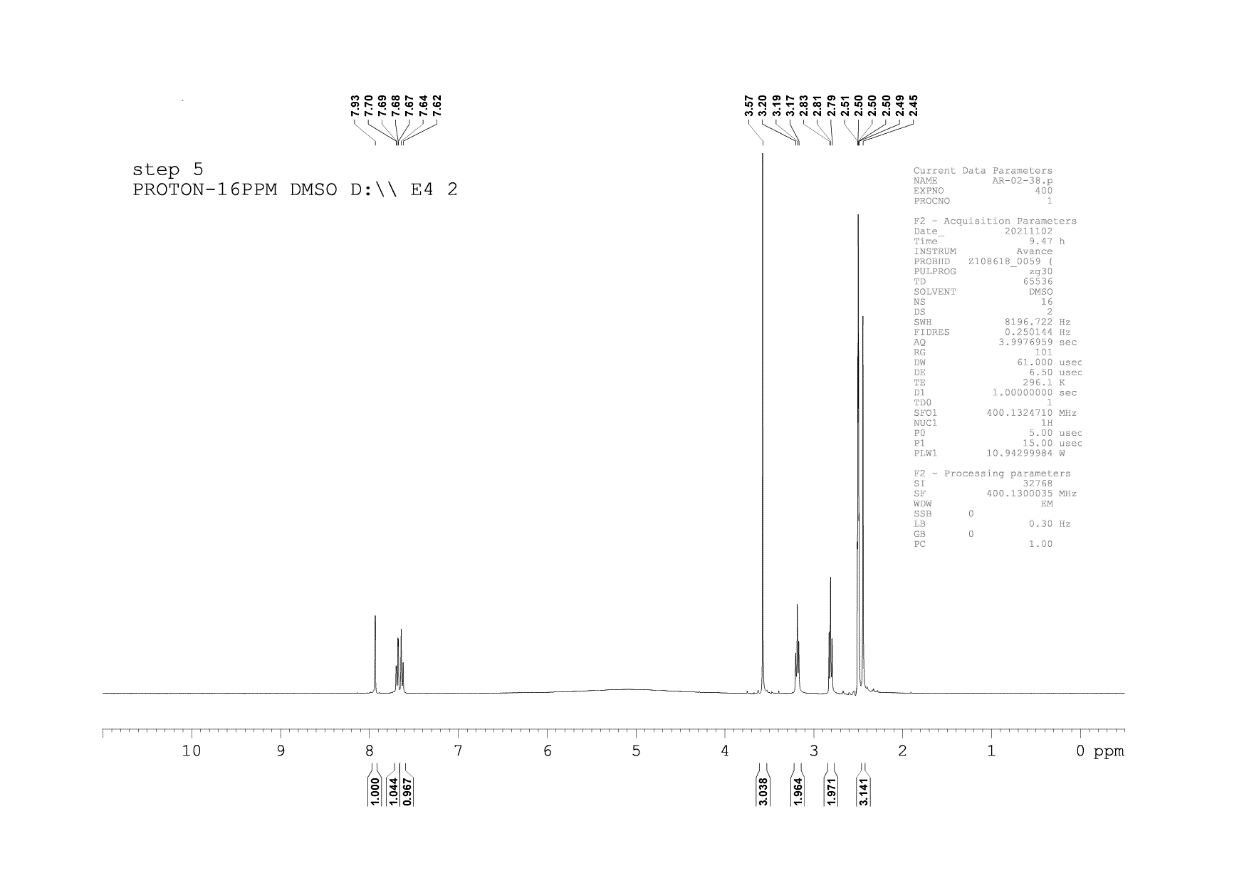


**Fig. S30**. ^1^H NMR (400 MHz, DMSO-*d_6_*) spectrum of compound **1d**


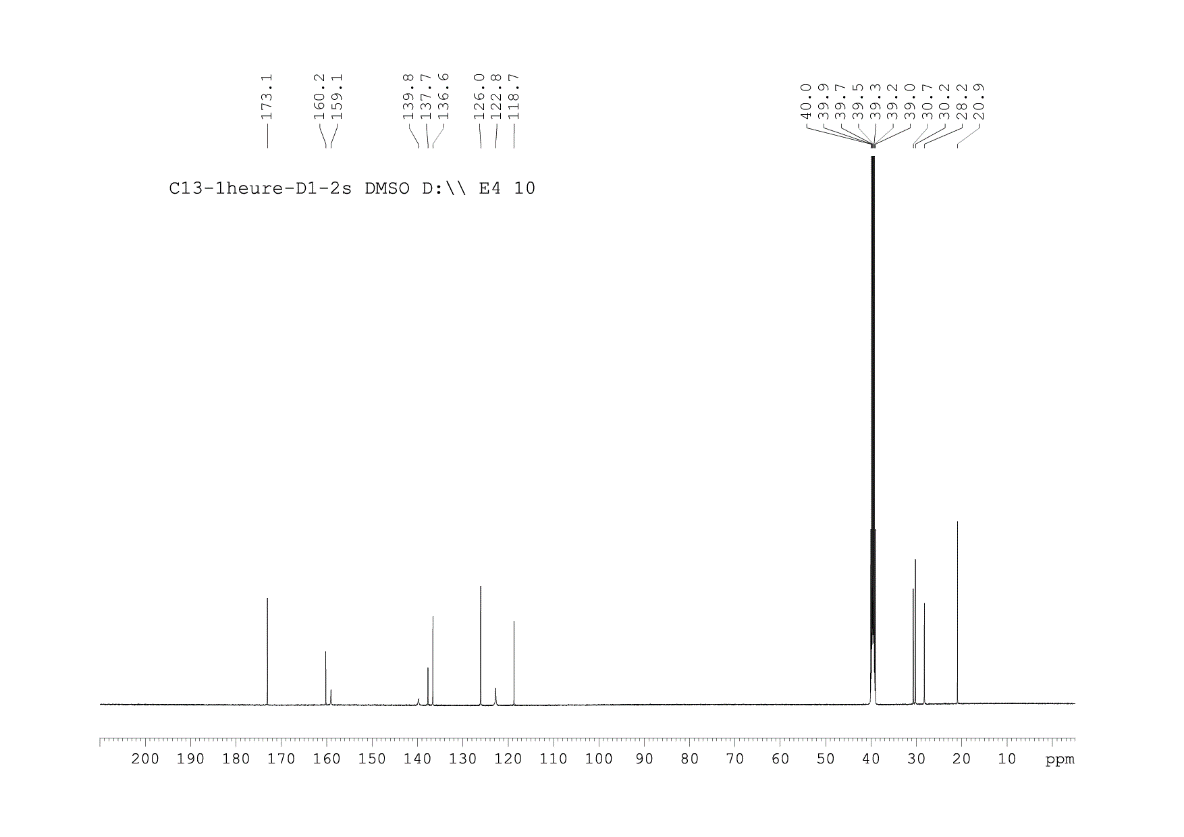


**Fig. S31**. ^13^C NMR (125 MHz, DMSO-*d_6_*) spectrum of compound **1d**


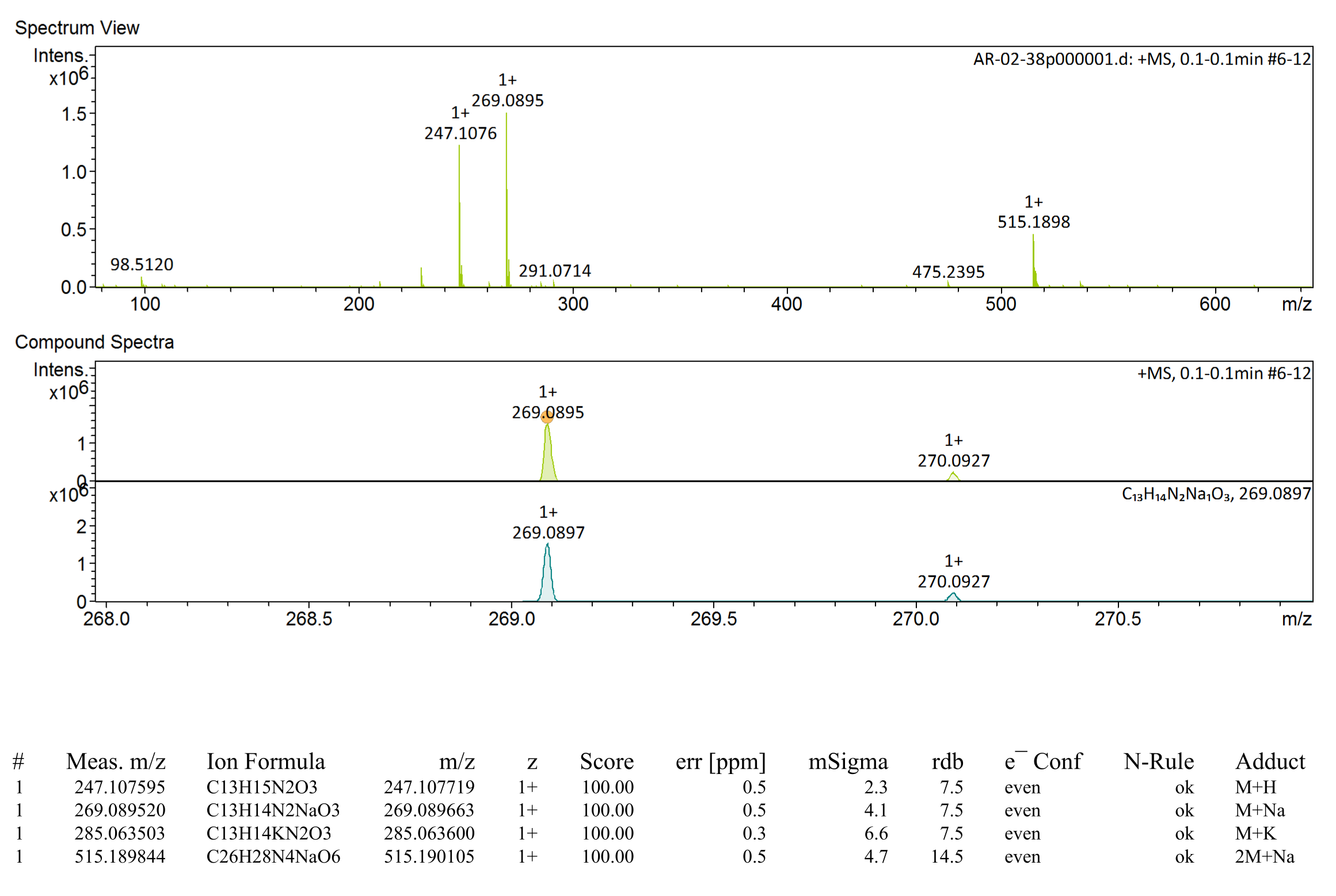


**Fig. S32**. HRMS spectrum of compound **1d**


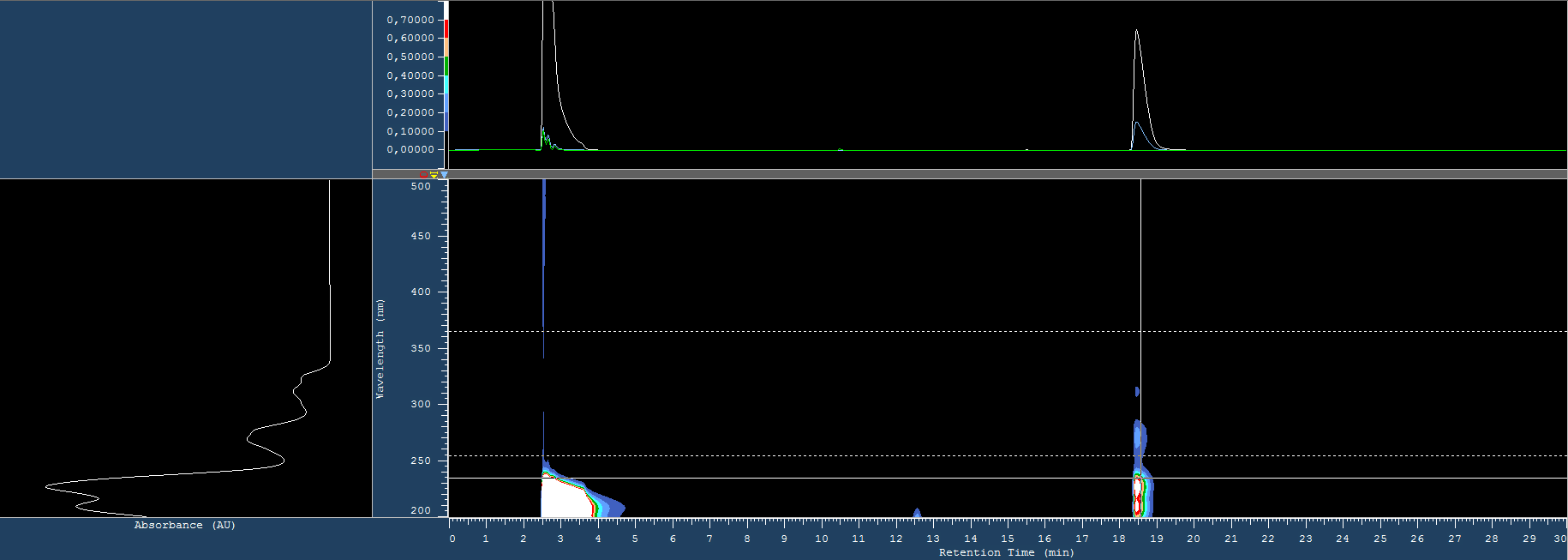


**Fig. S33**. HPLC-UV spectrum of compound **1d**


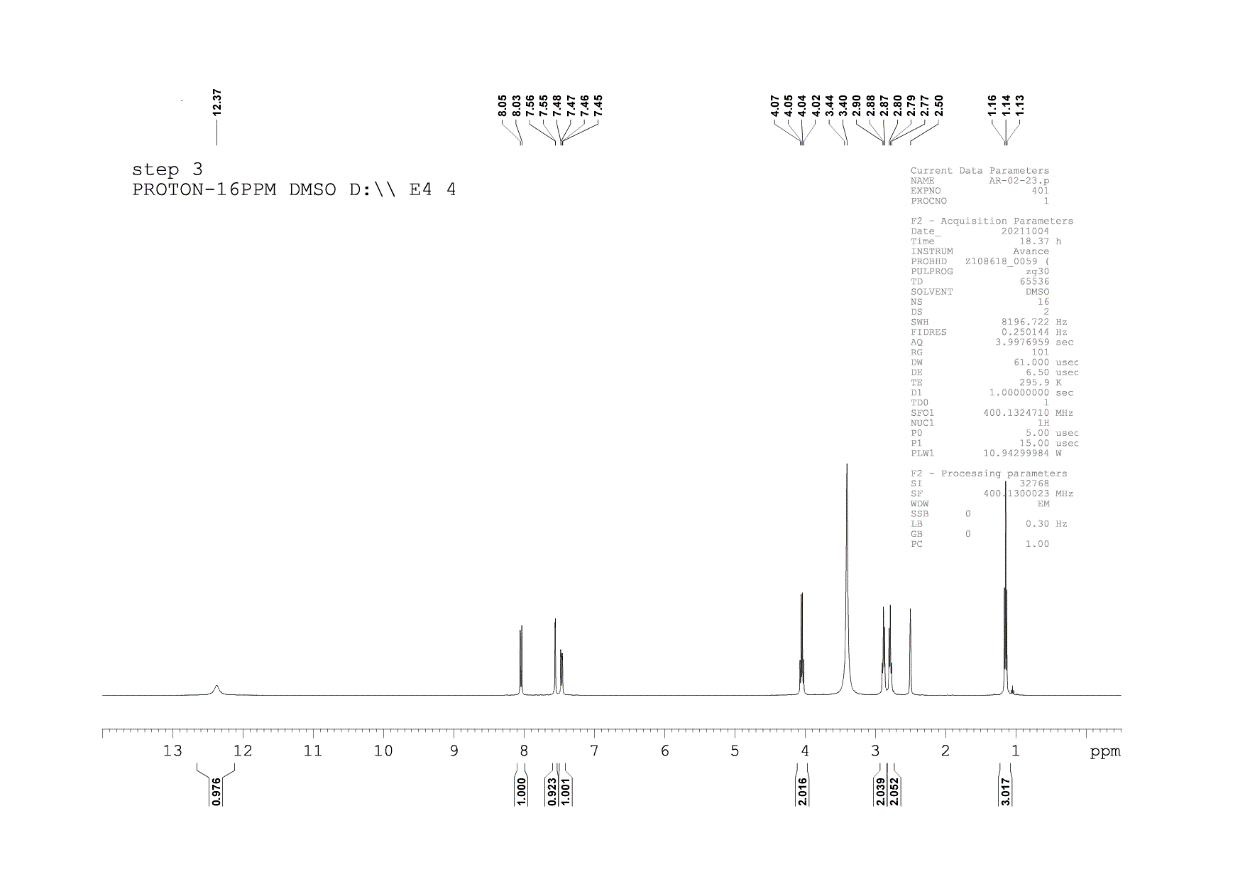


**Fig. S34**. ^1^H NMR (400 MHz, DMSO-*d_6_*) spectrum of compound **5e**


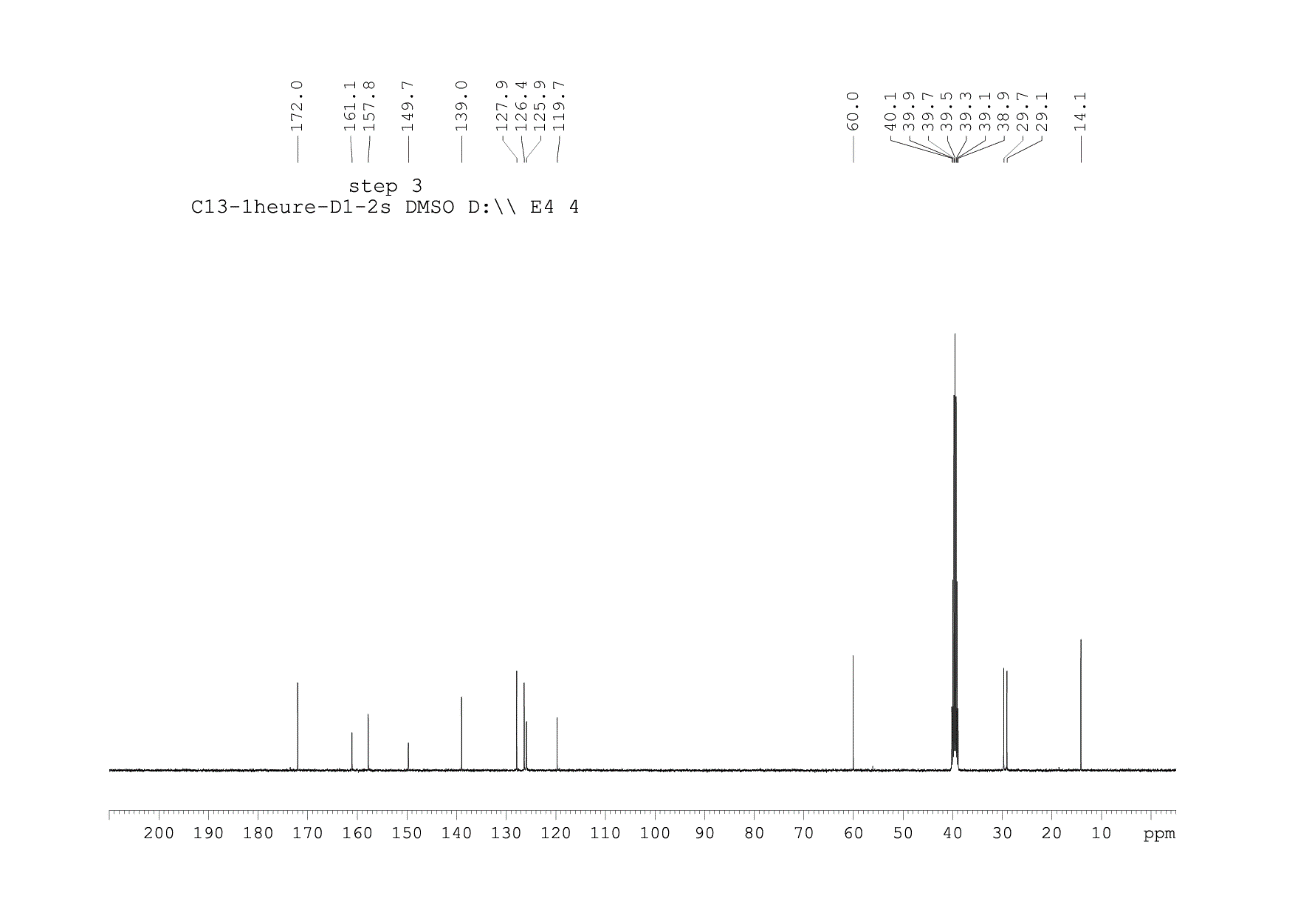


**Fig. S35**. ^13^C NMR (100 MHz, DMSO-*d_6_*) spectrum of compound **5e**


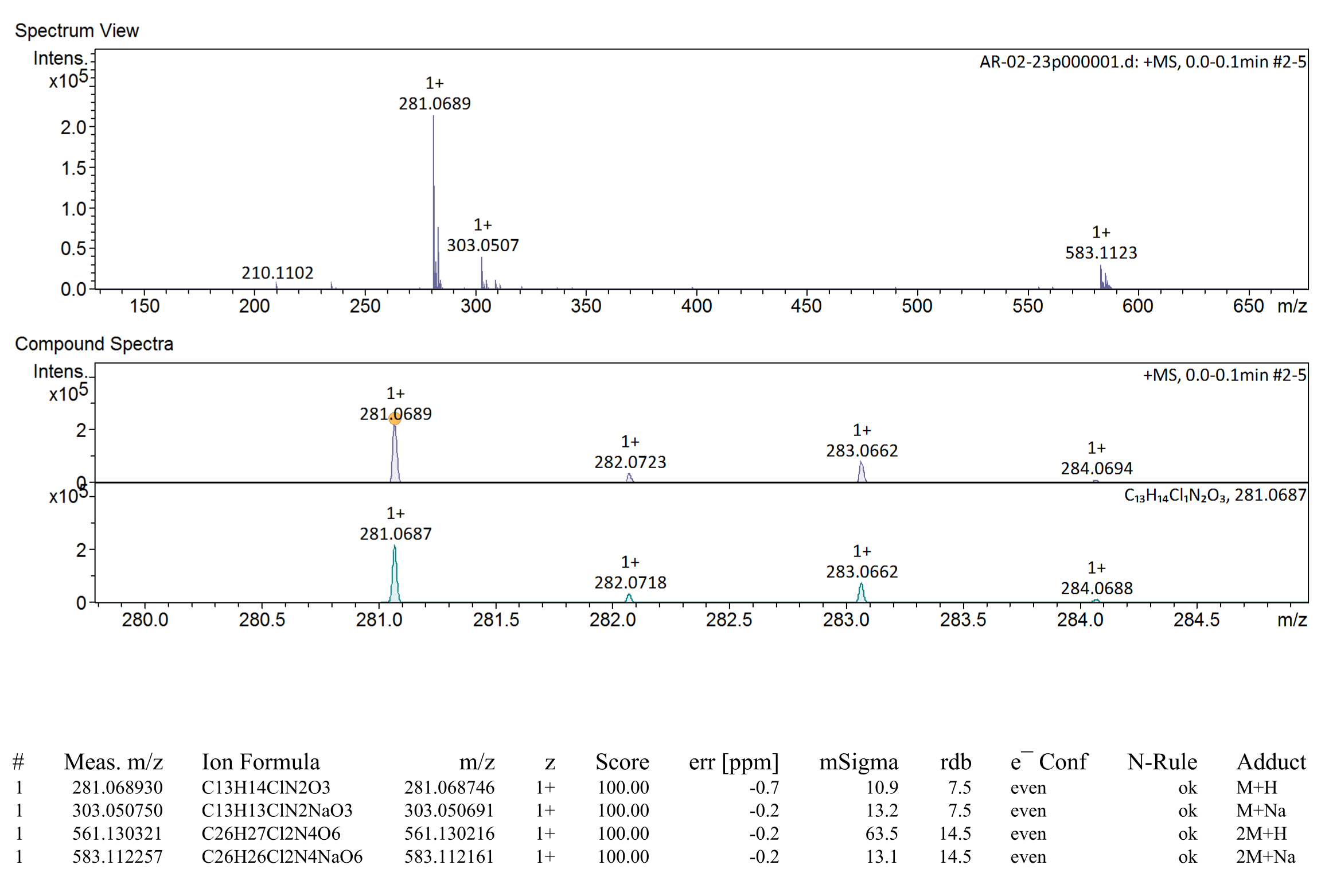


**Fig. S36**. HRMS spectrum of compound **5e**


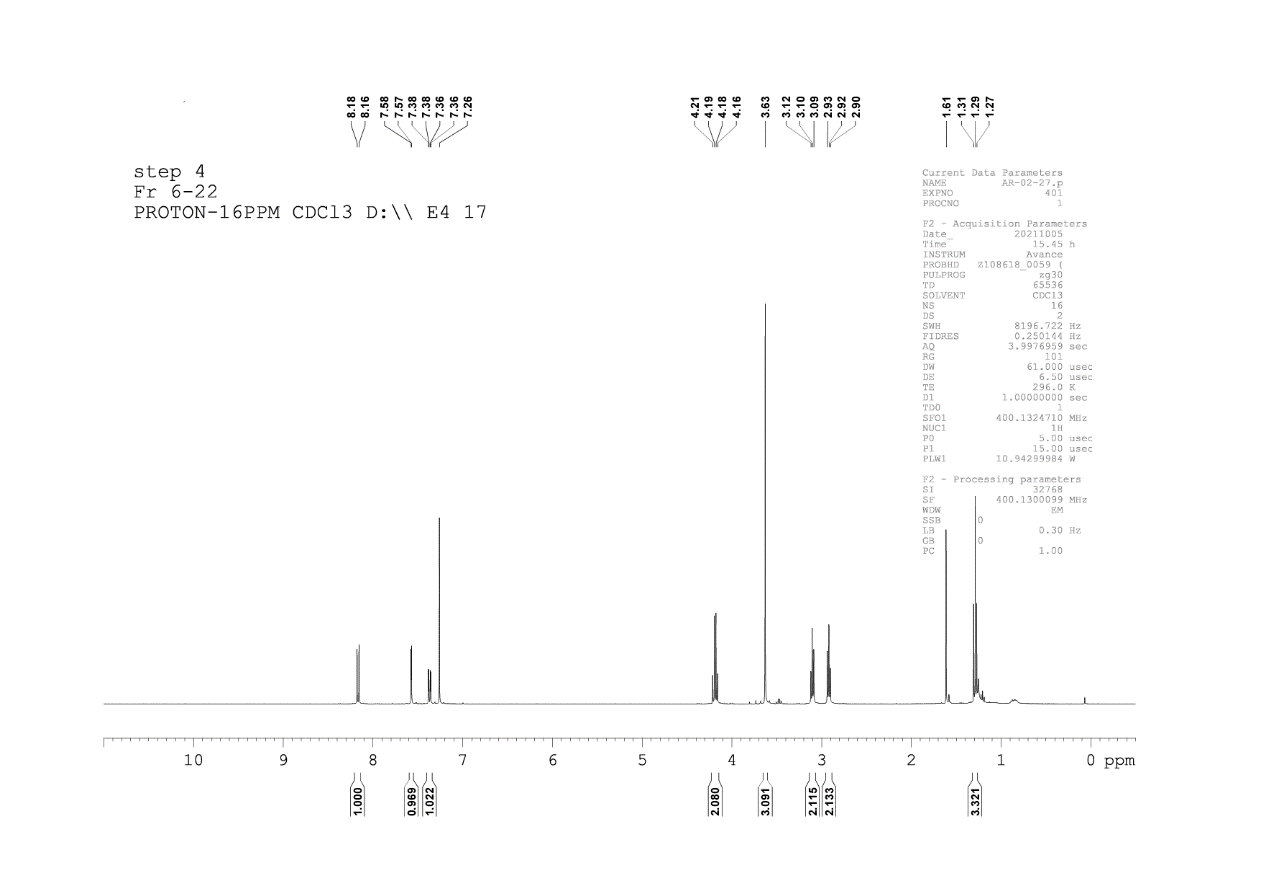


**Fig. S37**. ^1^H NMR (400 MHz, CDCl_3_) spectrum of compound **6e**


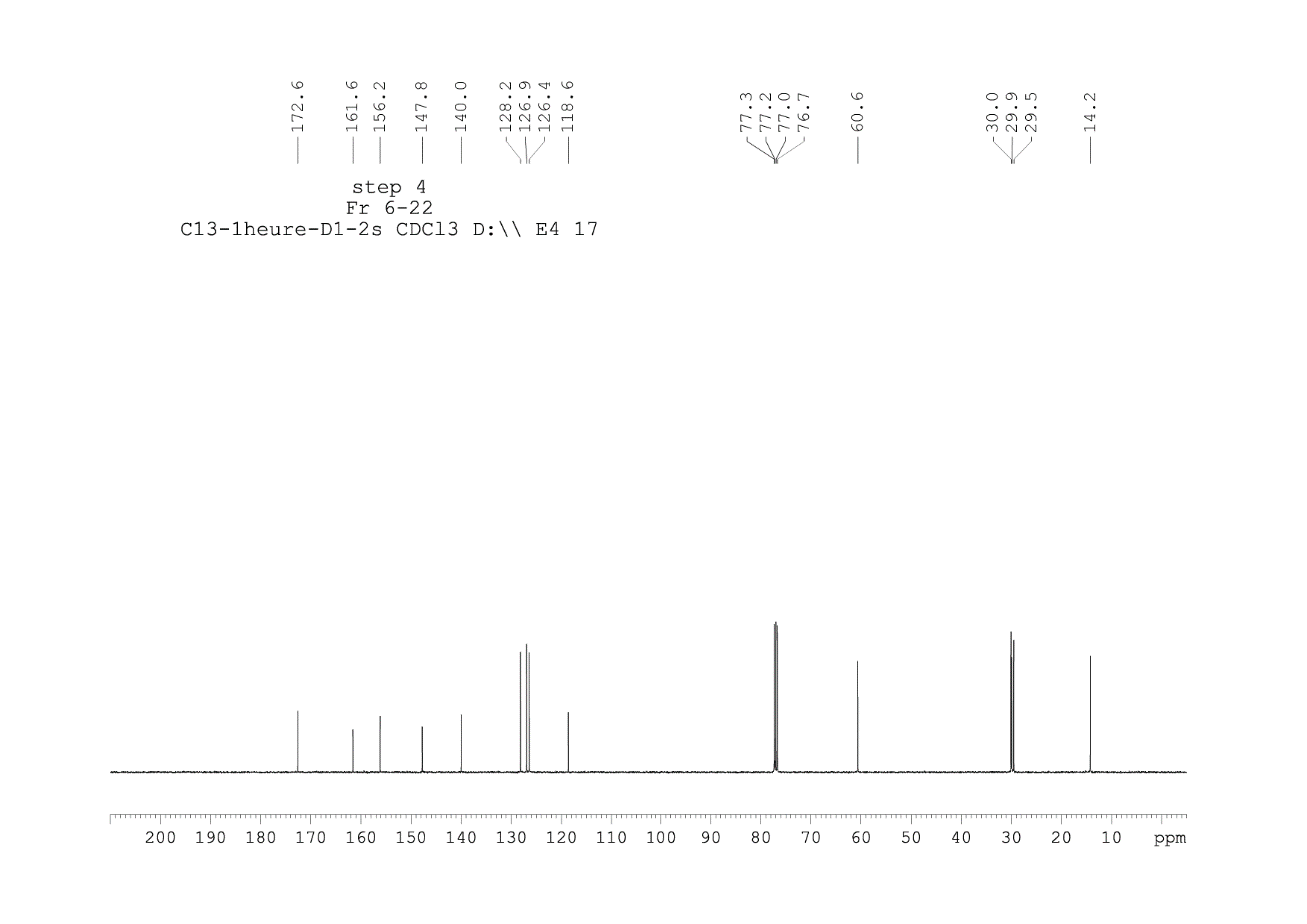


**Fig. S38**. ^13^C NMR (100 MHz, CDCl_3_) spectrum of compound **6e**


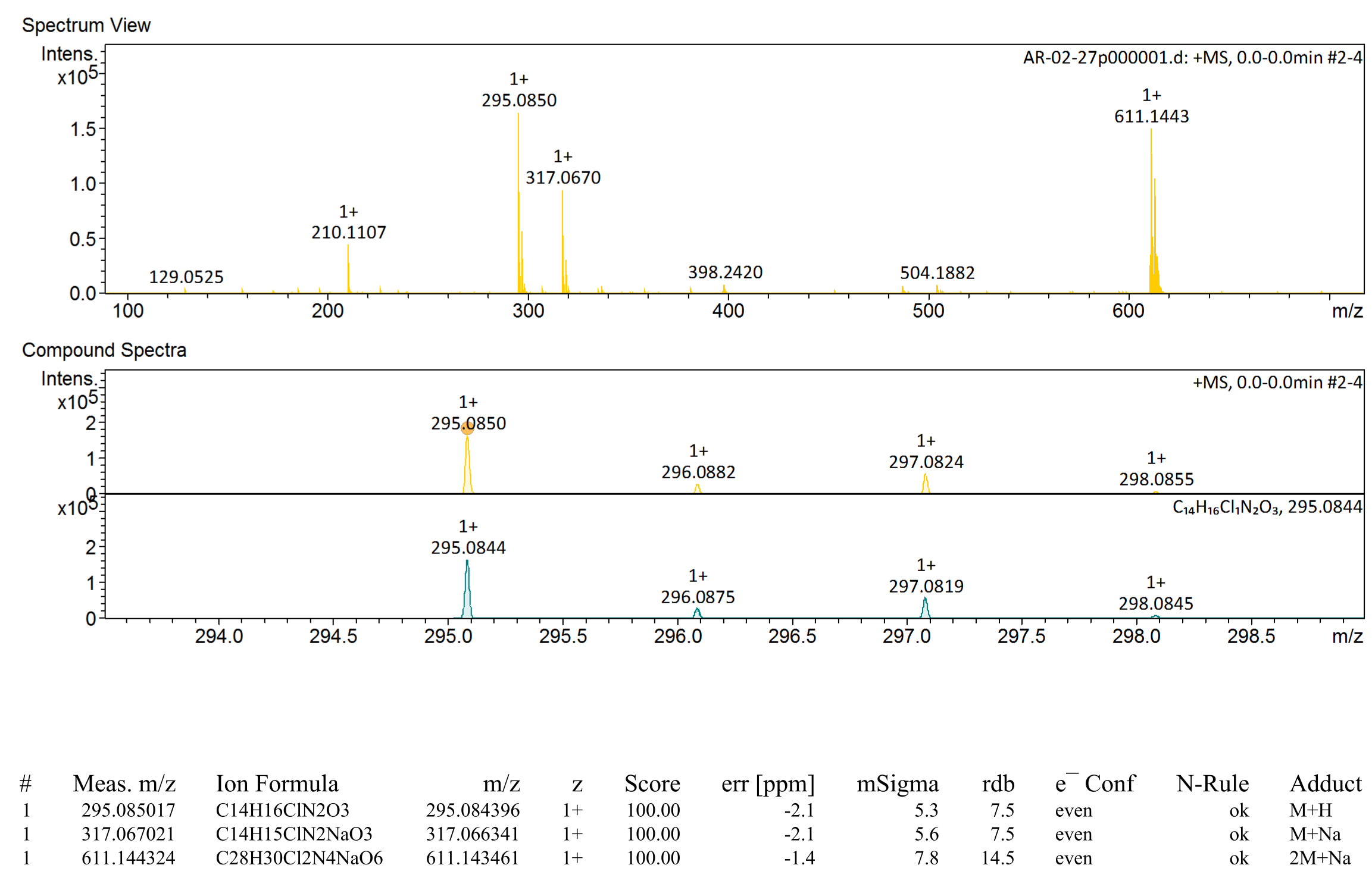


**Fig. S39**. HRMS spectrum of compound **6e**


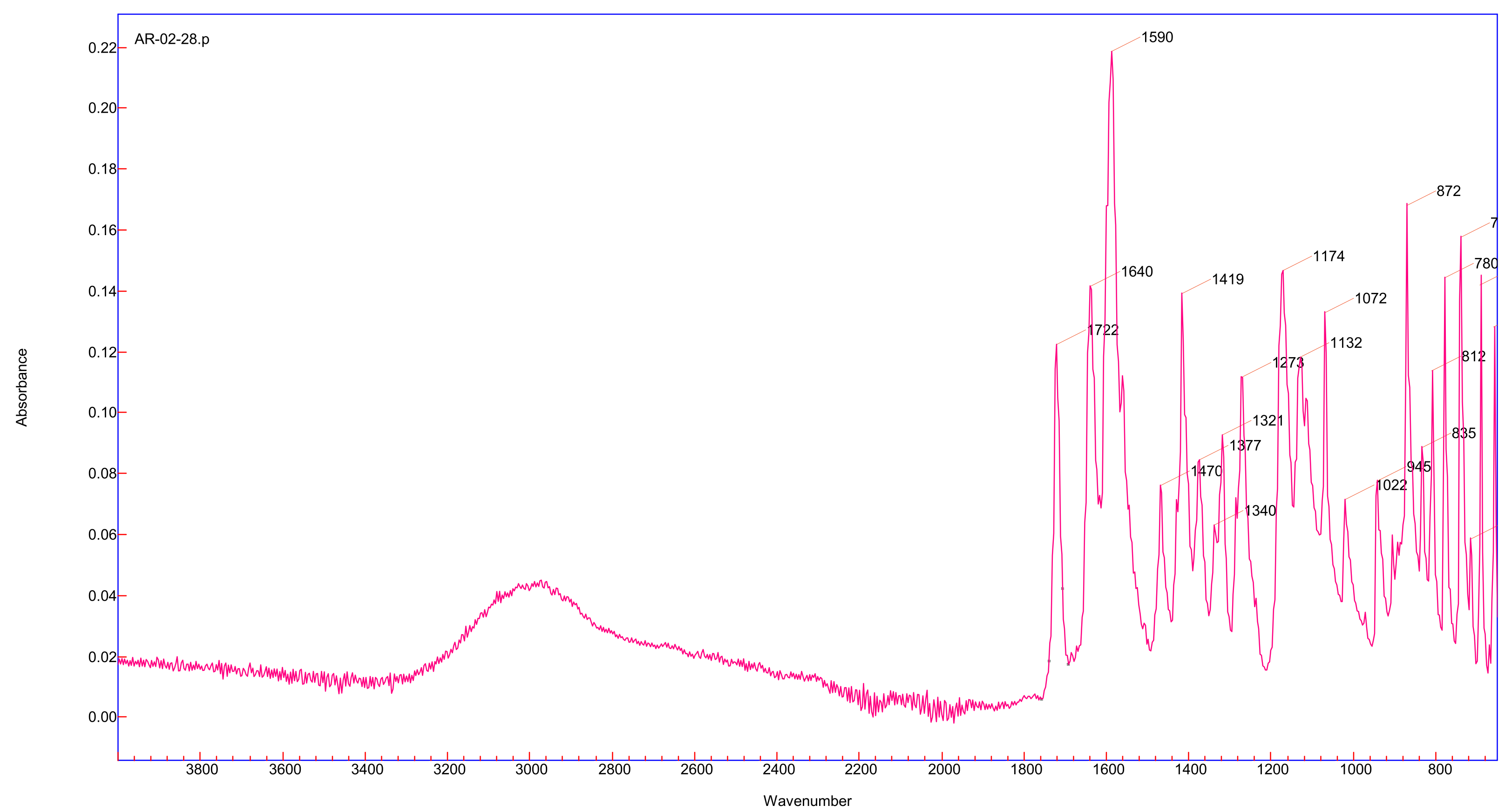


**Fig. S40**. IR spectrum of compound **1e**


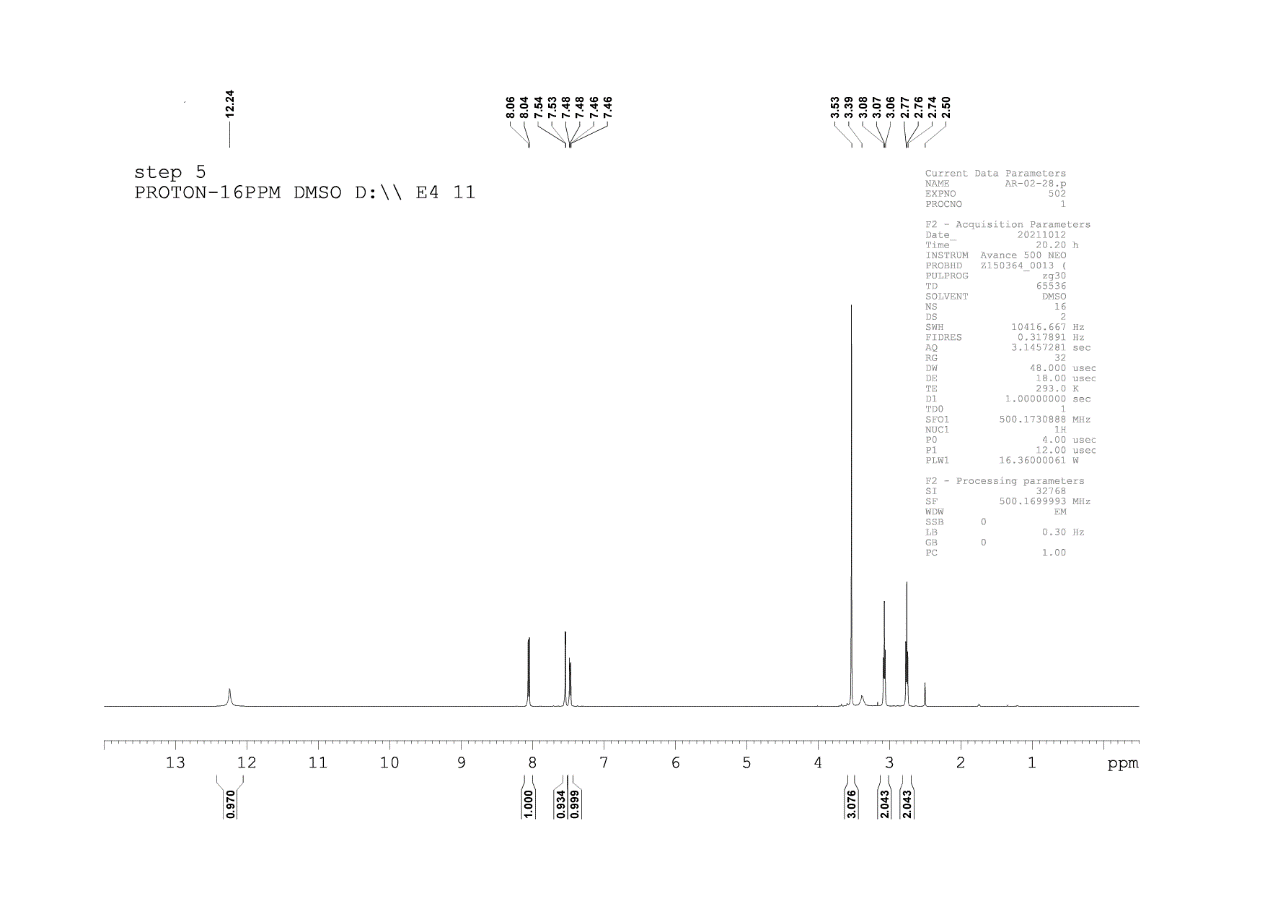


**Fig. S41**. ^1^H NMR (500 MHz, DMSO-*d_6_*) spectrum of compound **1e**


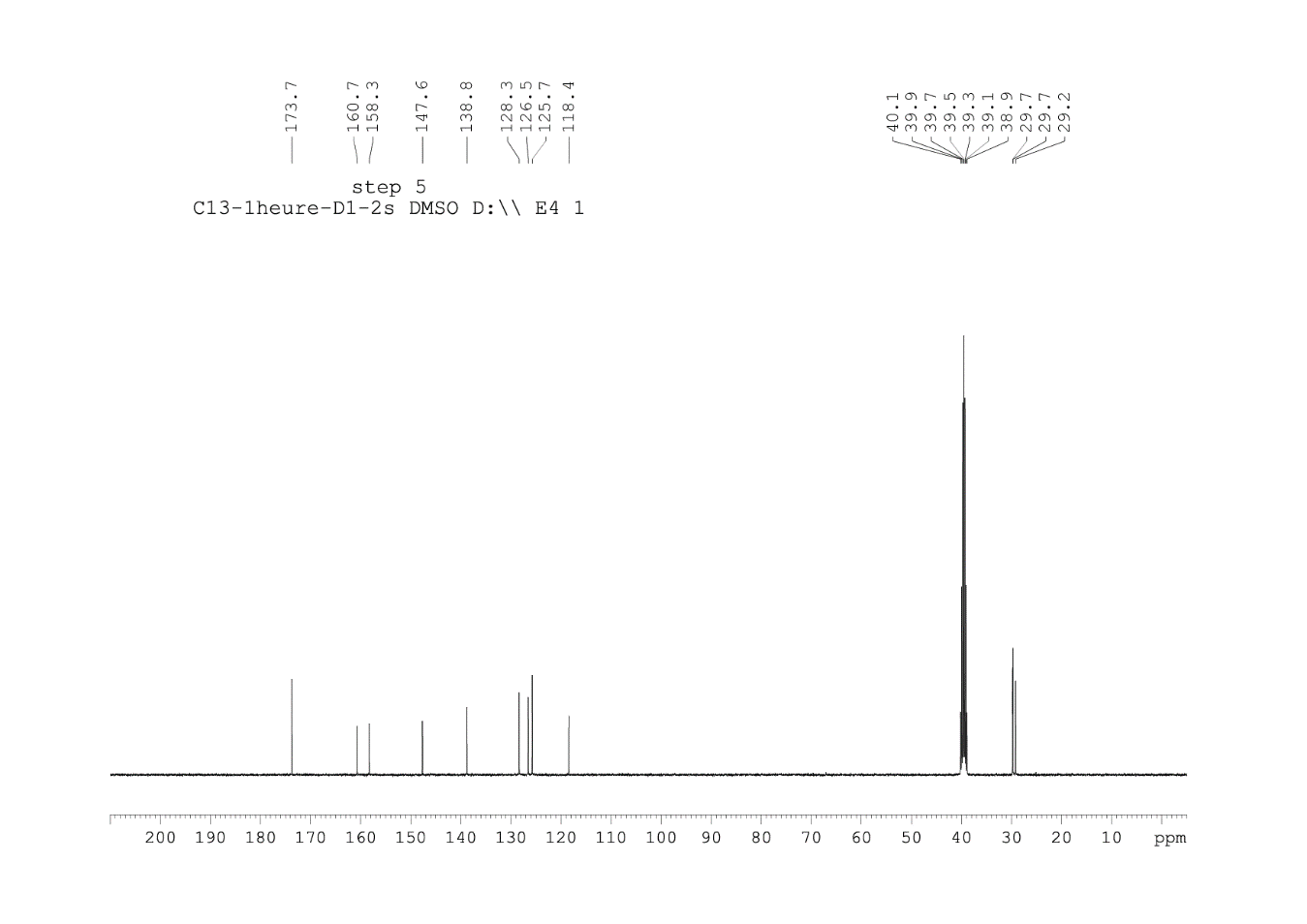


**Fig. S42**. ^13^C NMR (100 MHz, DMSO-*d_6_*) spectrum of compound **1e**


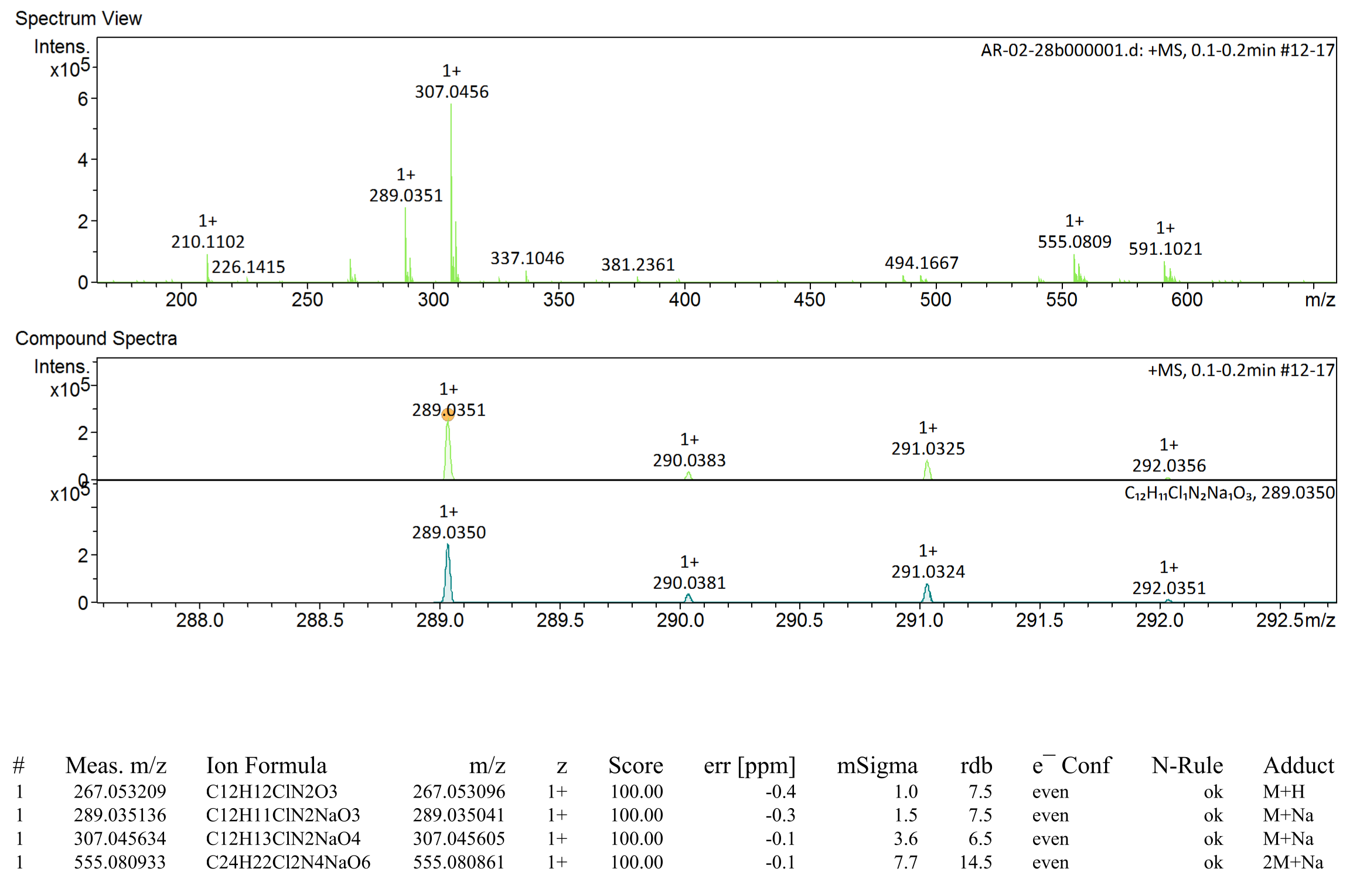


**Fig. S43**. HRMS spectrum of compound **1e**


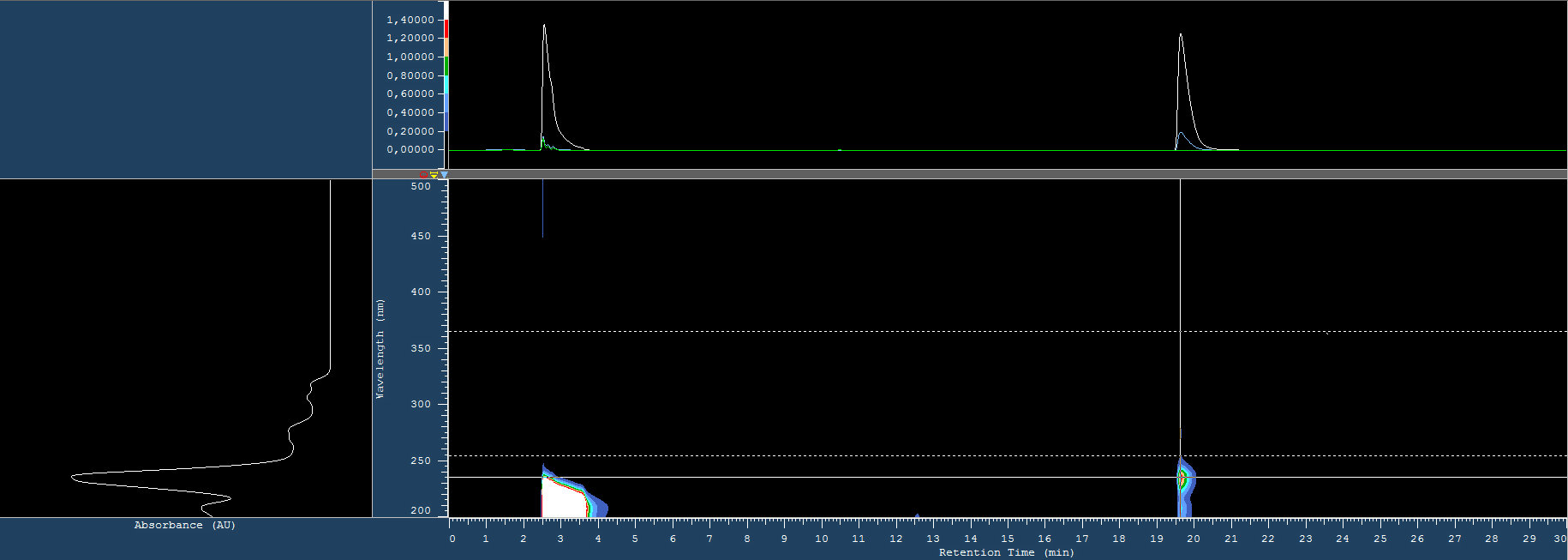


**Fig. S44**. HPLC-UV spectrum of compound **1e**


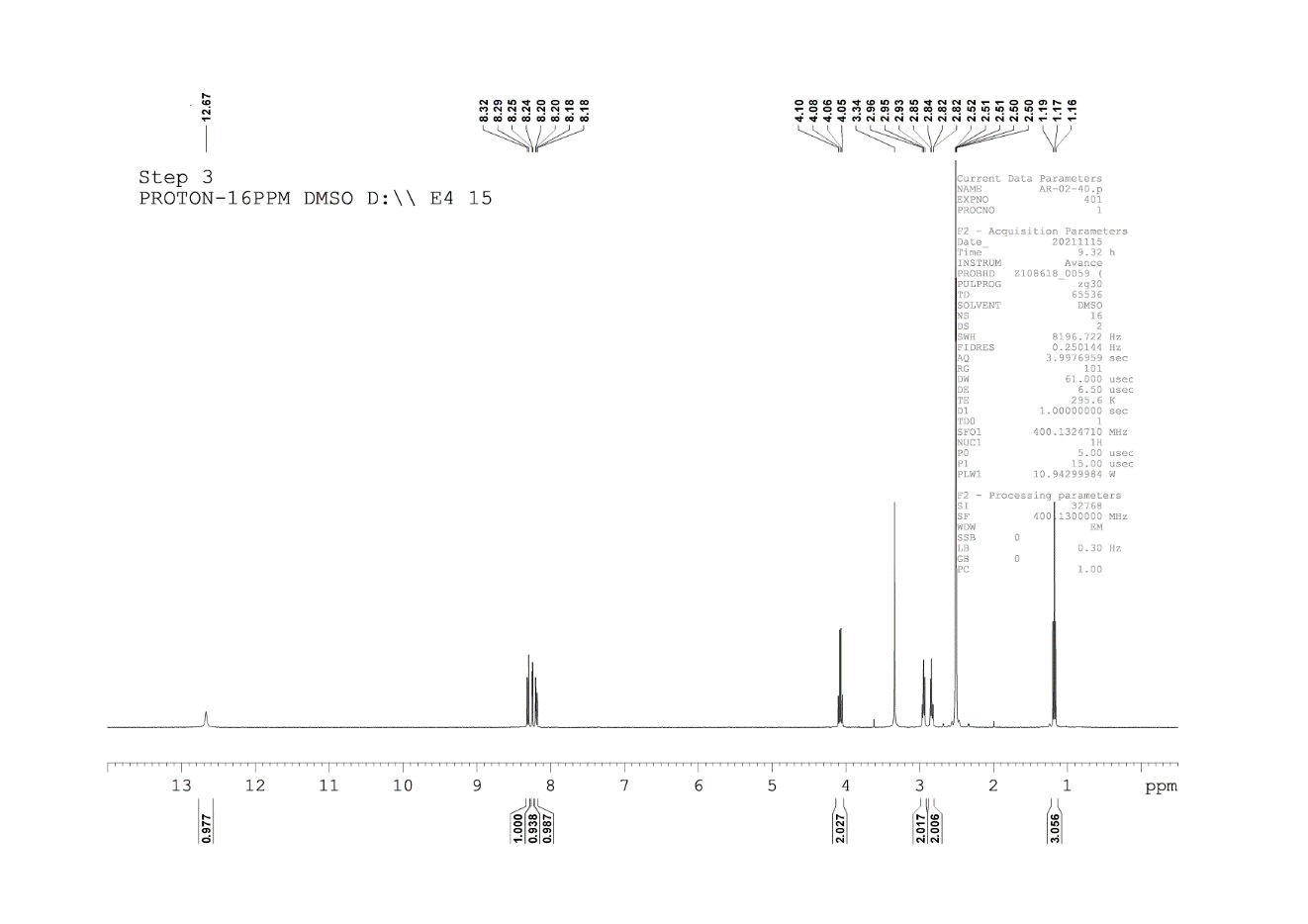


**Fig. S45**. ^1^H NMR (400 MHz, DMSO-*d_6_*) spectrum of compound **5f**


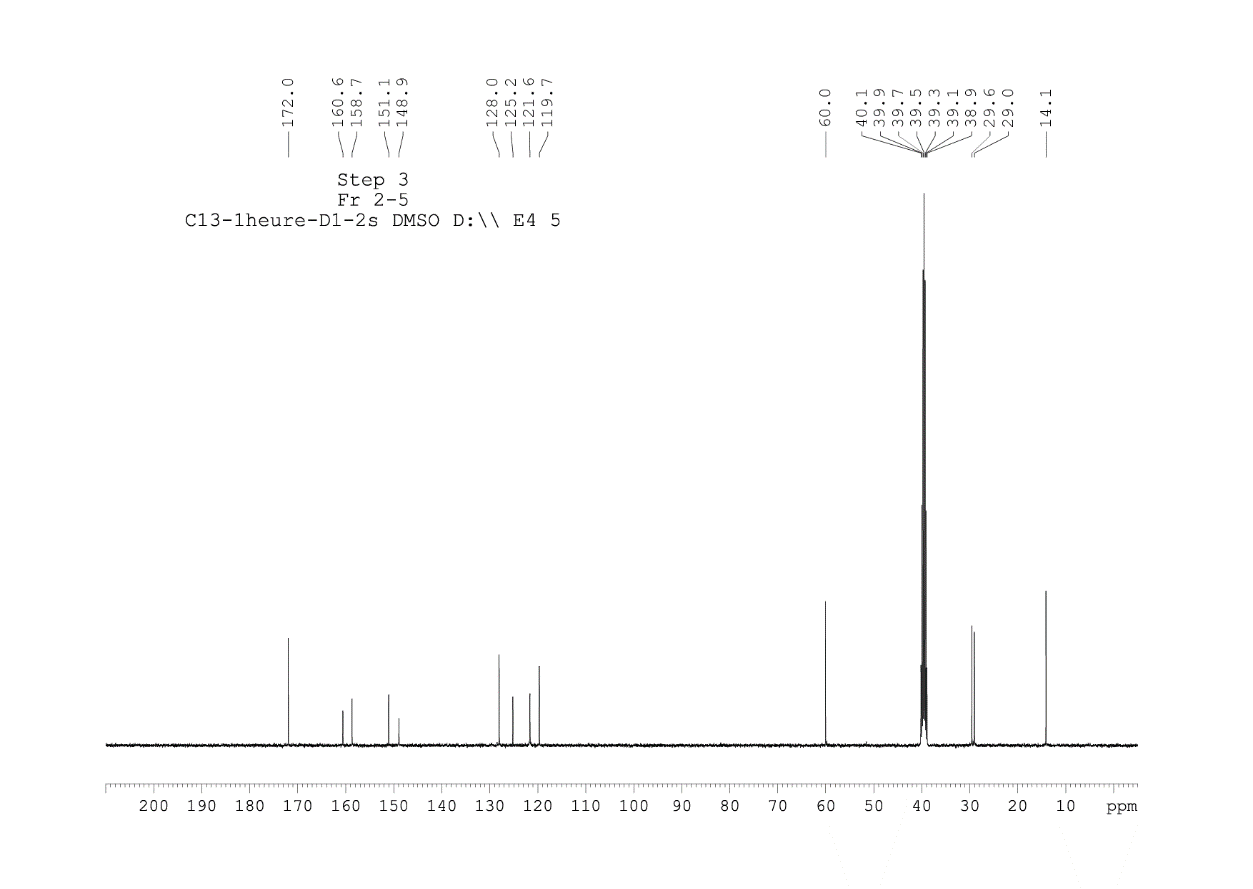


**Fig. S46**. ^13^C NMR (100 MHz, DMSO-*d_6_*) spectrum of compound **5f**


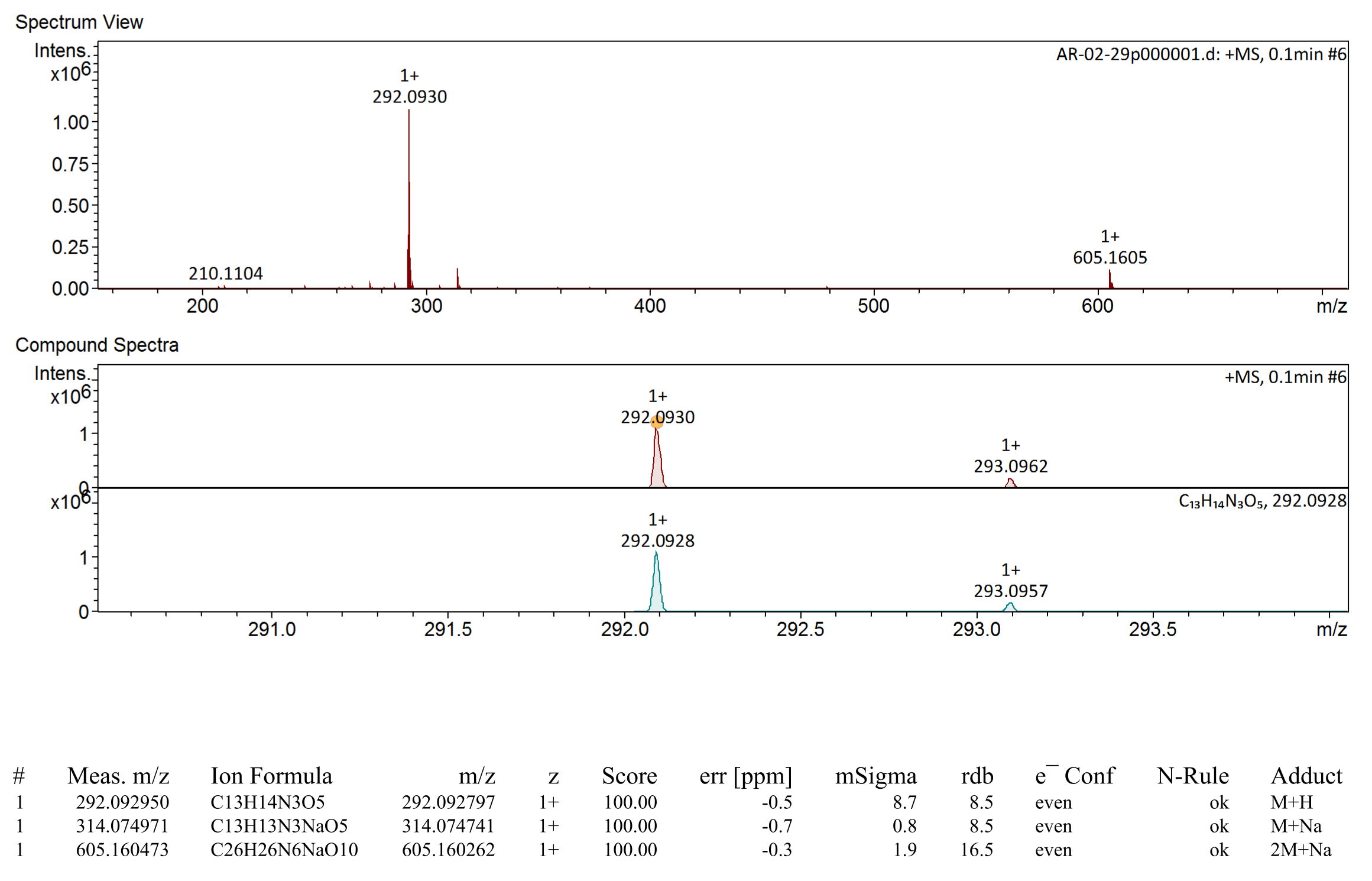


**Fig. S47**. HRMS spectrum of compound **5f**


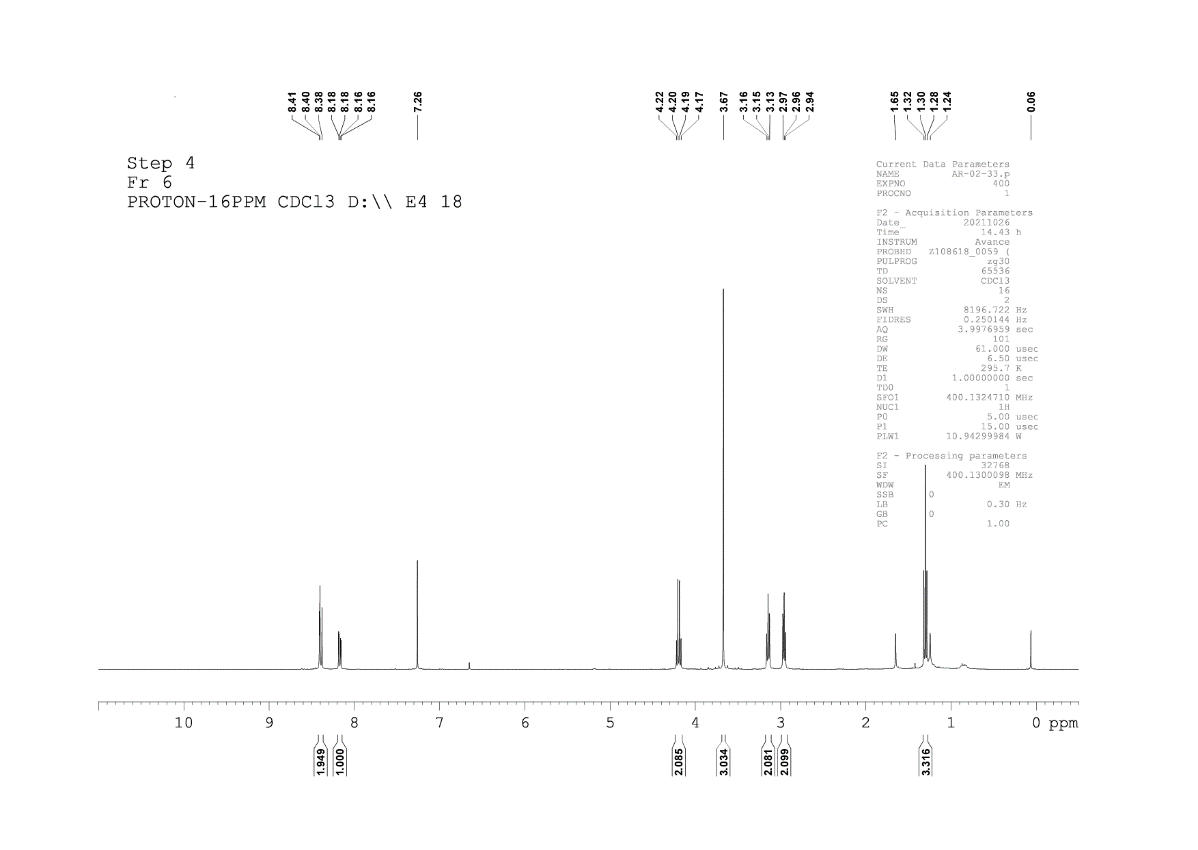


**Fig. S48**. ^1^H NMR (400 MHz, CDCl_3_) spectrum of compound **6f**


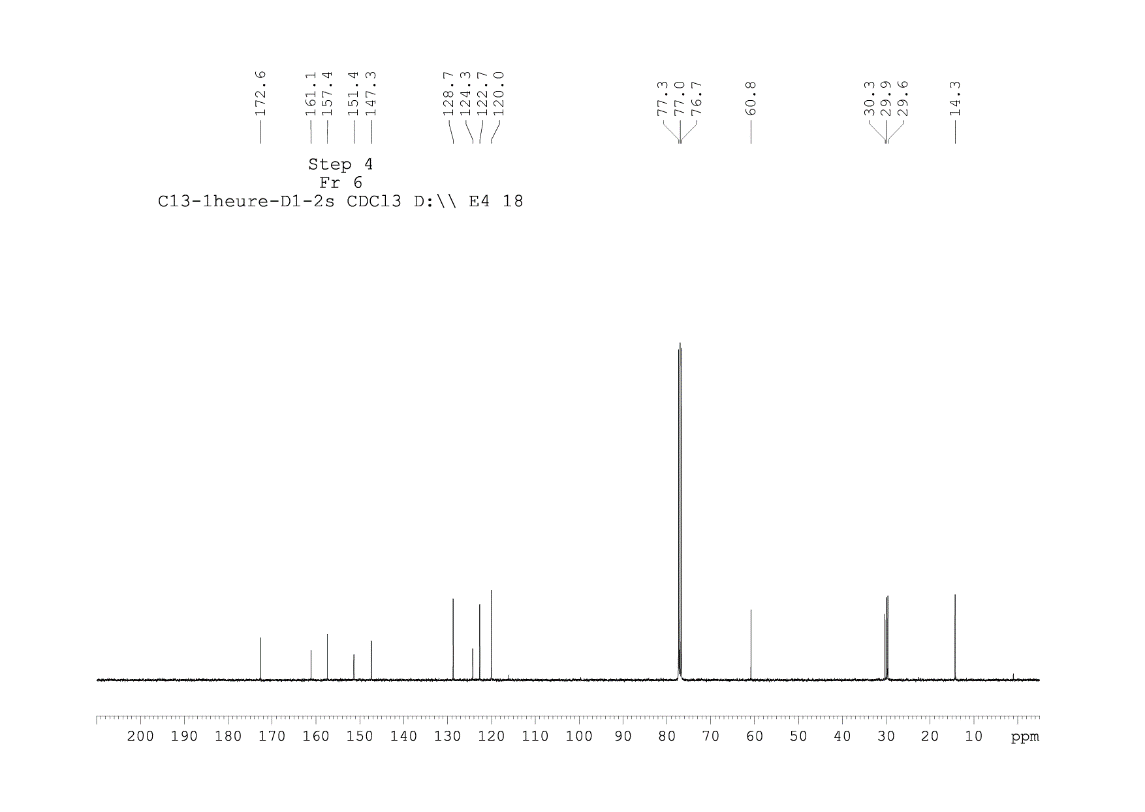


**Fig. S49**. ^13^C NMR (100 MHz, CDCl_3_) spectrum of compound **6f**


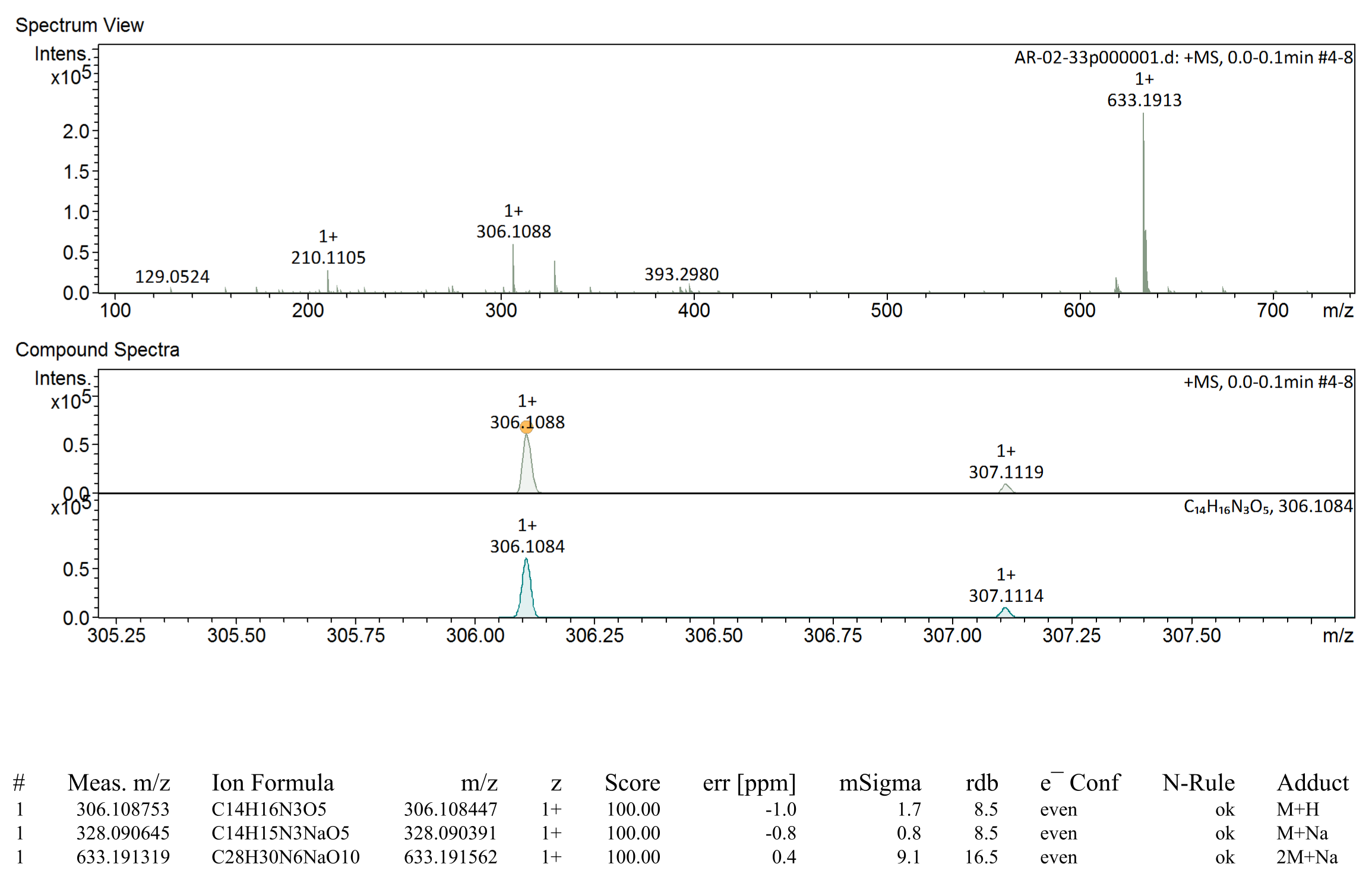


**Fig. S50**. HRMS spectrum of compound **6f**


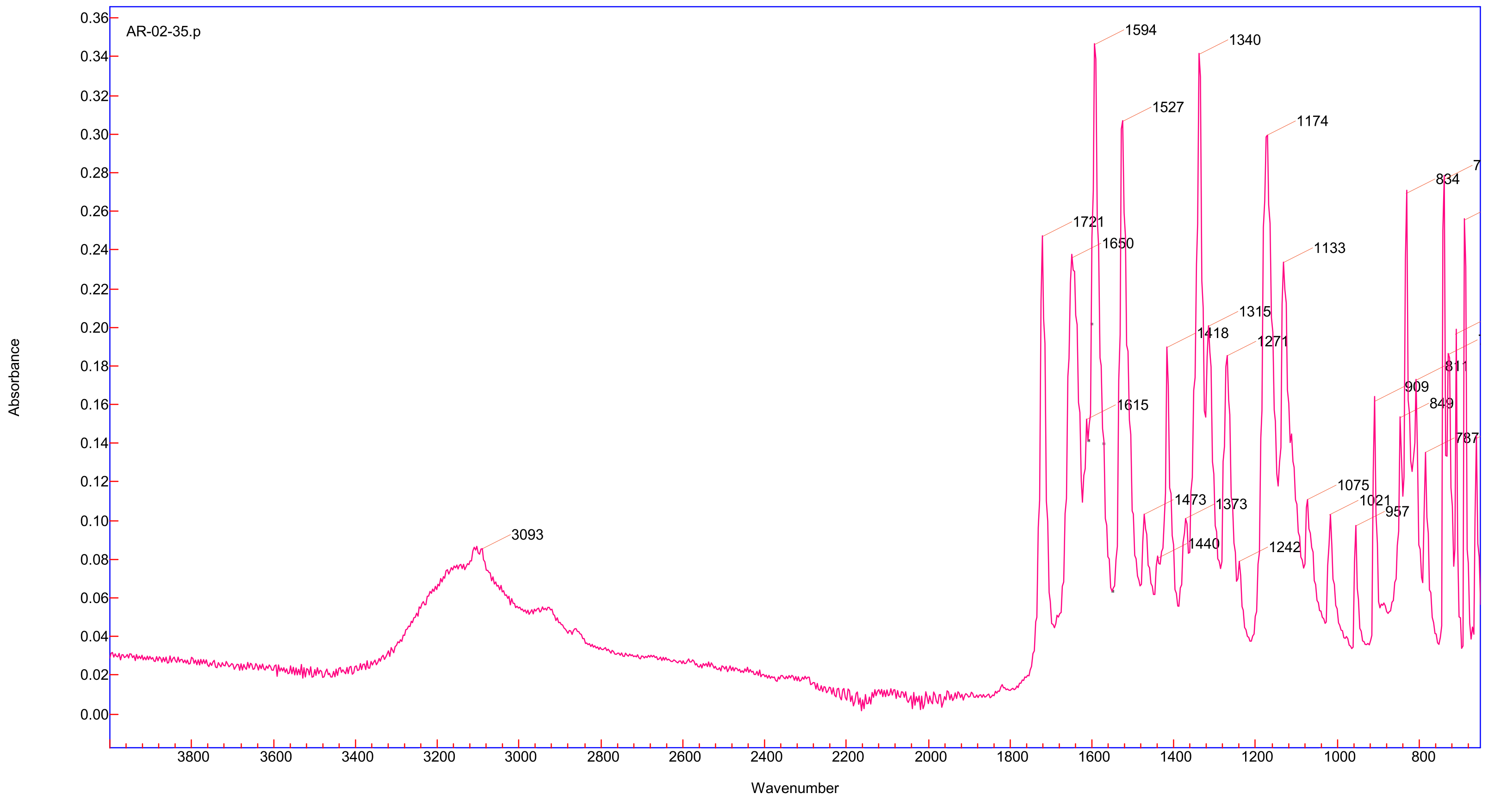


**Fig. S51**. IR spectrum of compound **1f**


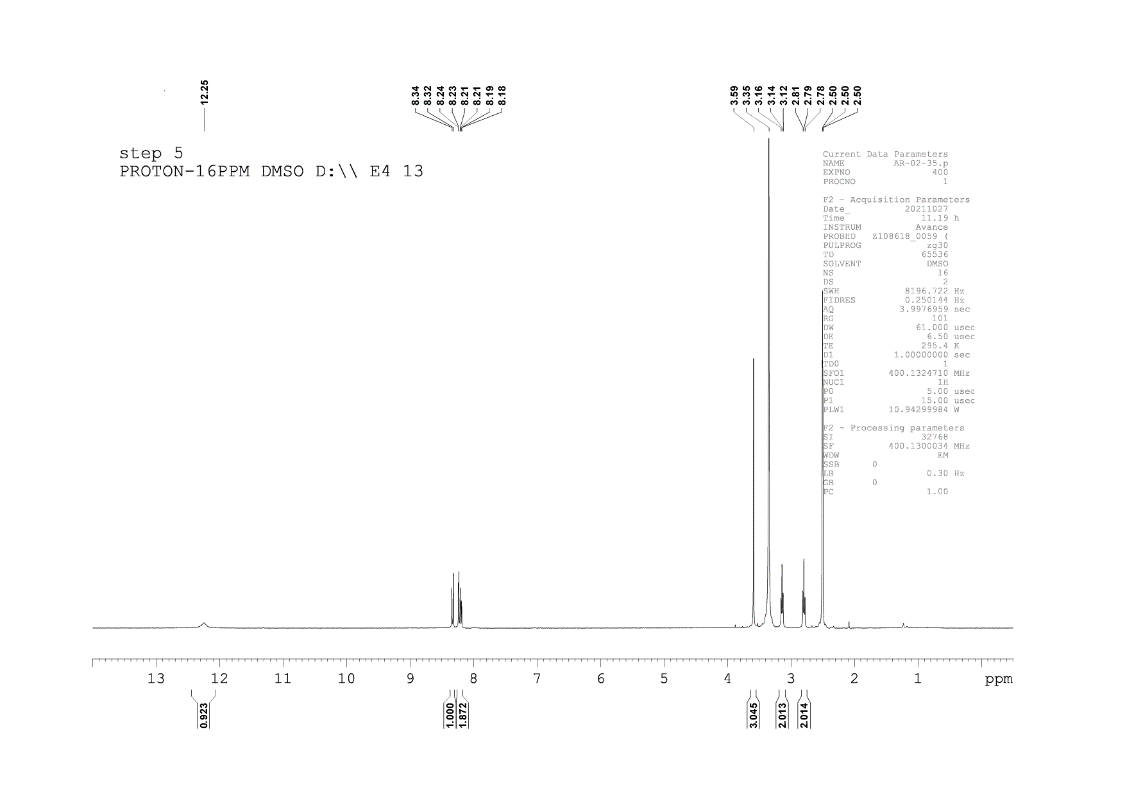


**Fig. S52**. ^1^H NMR (400 MHz, DMSO-*d_6_*) spectrum of compound **1f**


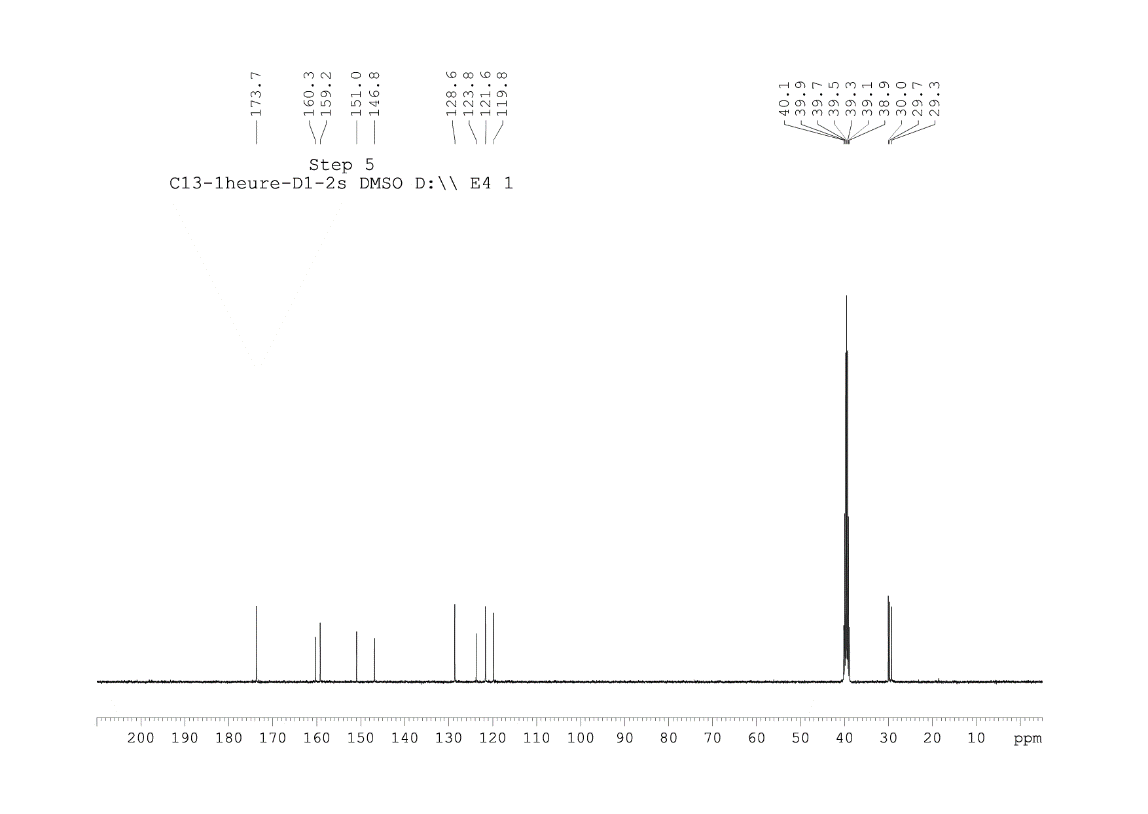


**Fig. S53**. ^13^C NMR (100 MHz, DMSO-*d_6_*) spectrum of compound **1f**


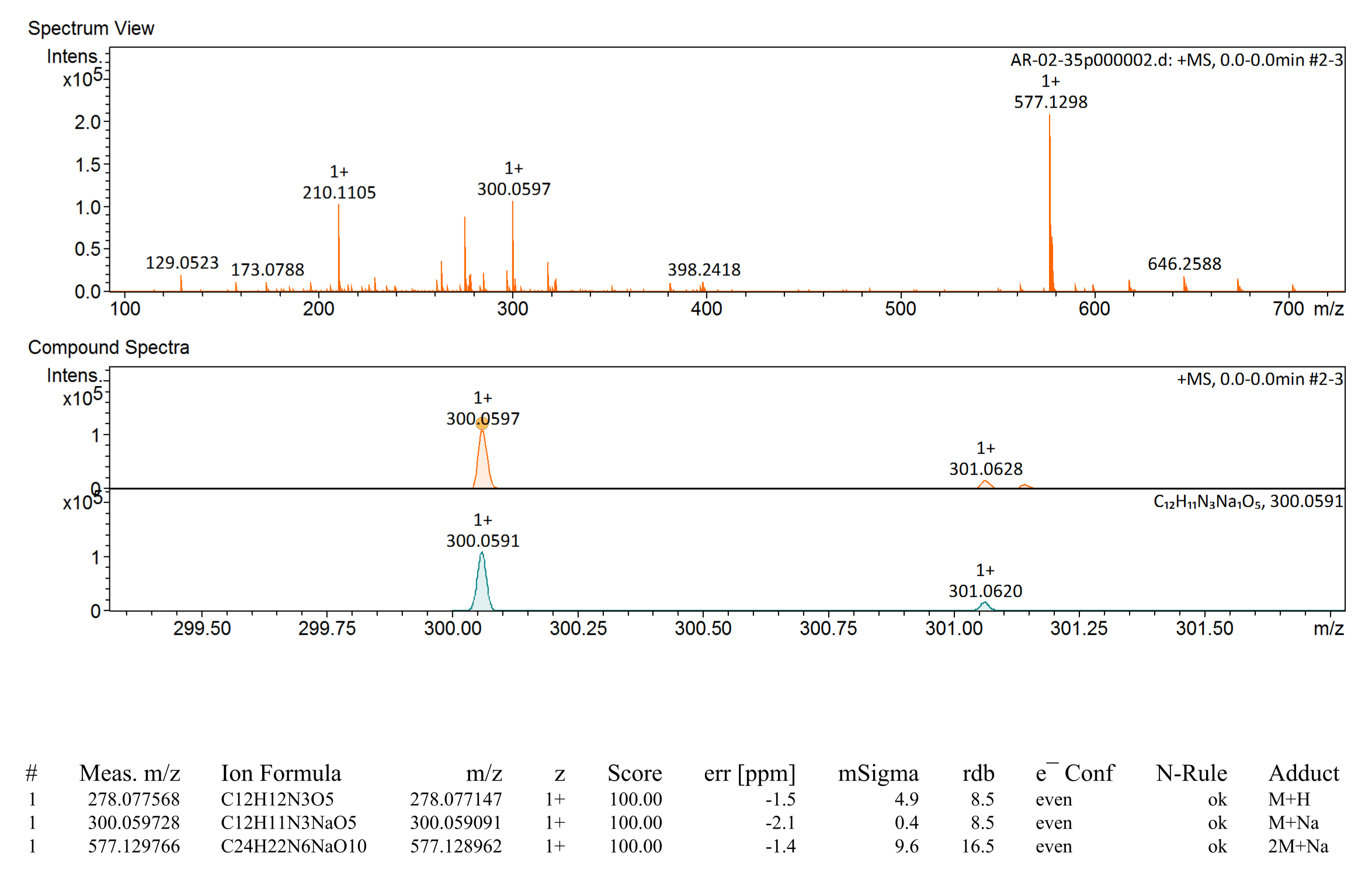


**Fig. S54**. HRMS spectrum of compound **1f**


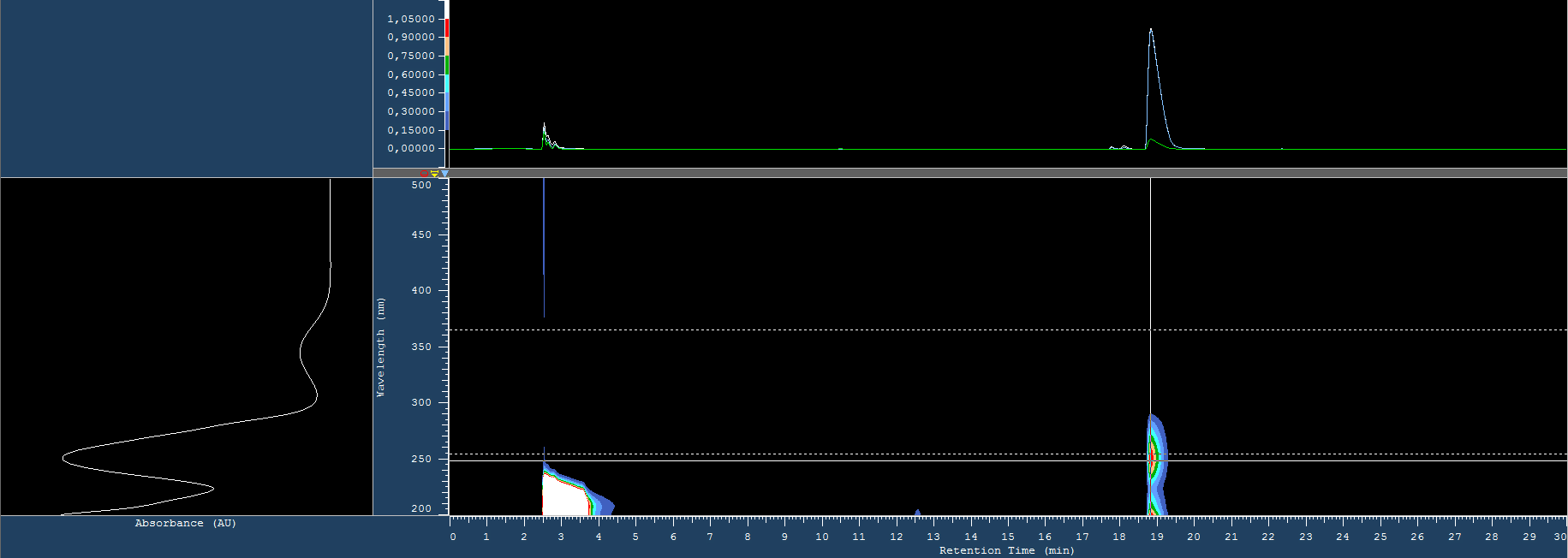


**Fig. S55**. HPLC-UV spectrum of compound **1f**


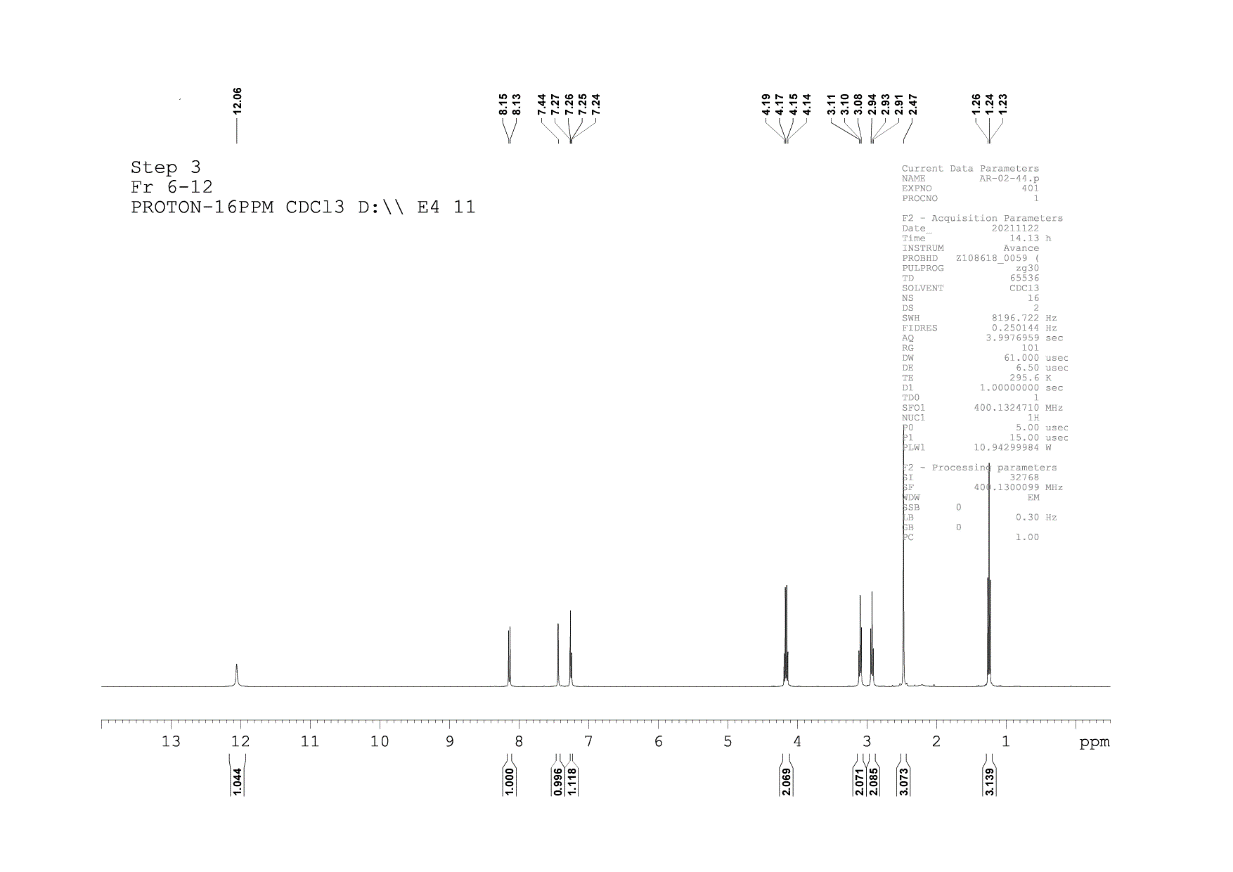


**Fig. S56**. ^1^H NMR (400 MHz, CDCl_3_) spectrum of compound **5g**


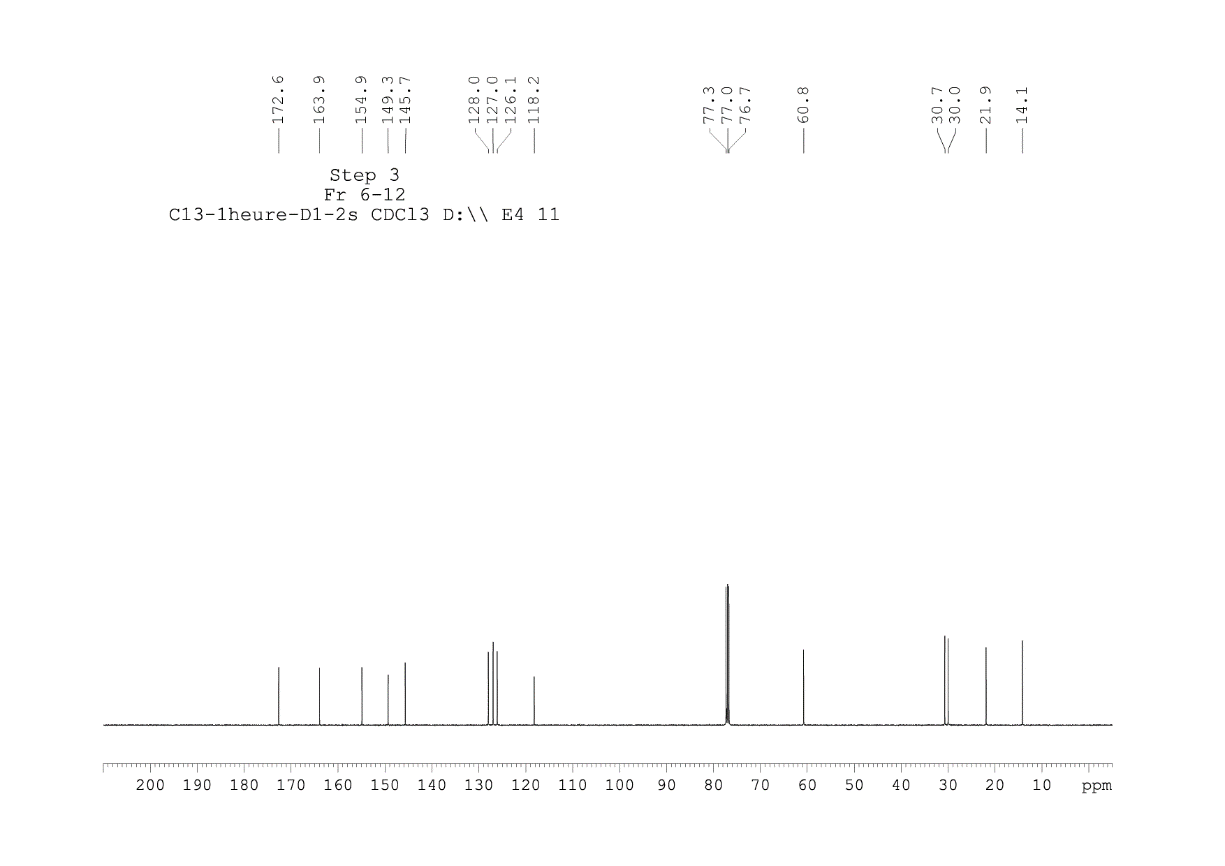


**Fig. S57**. ^13^C NMR (100 MHz, CDCl_3_) spectrum of compound **5g**


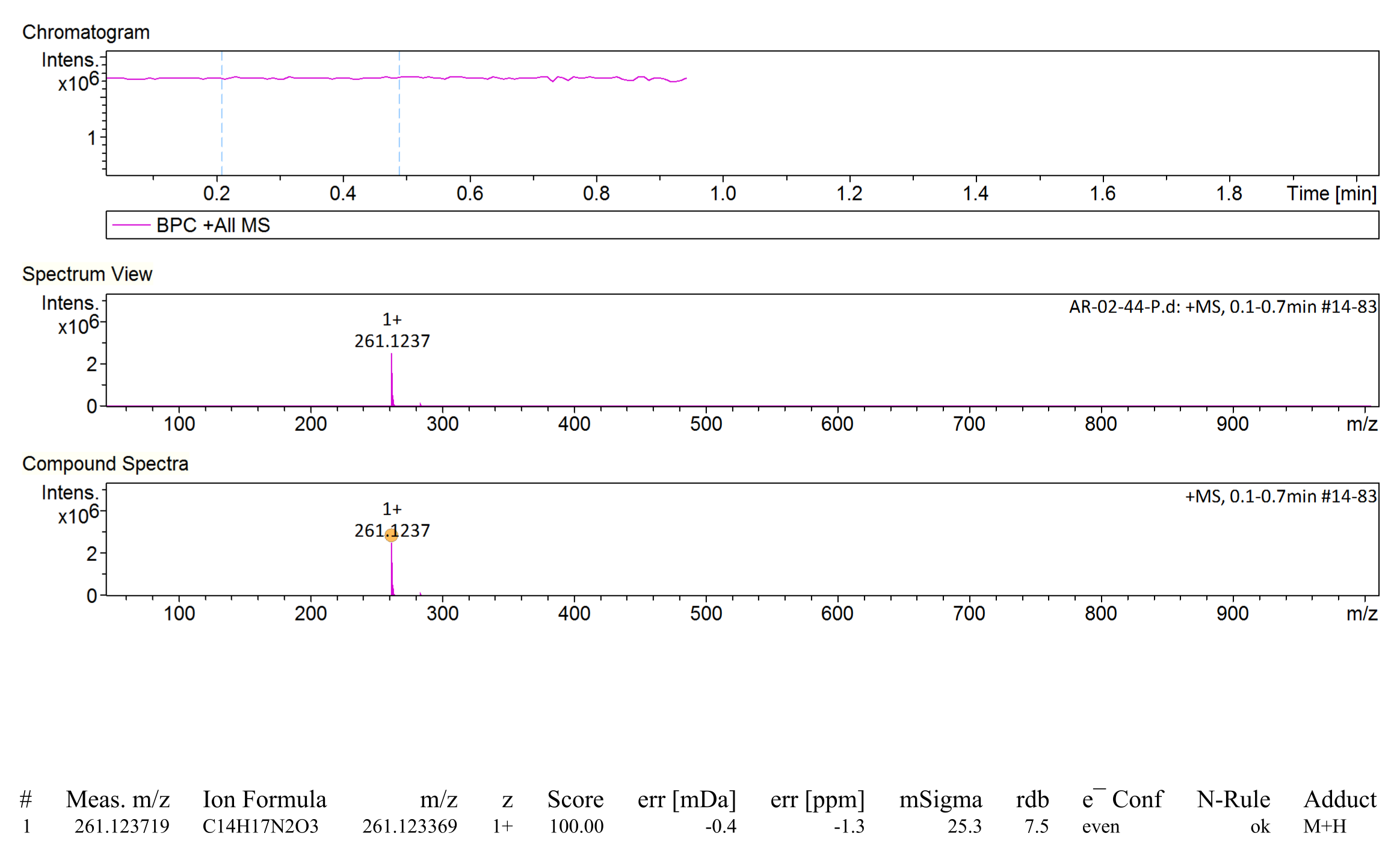


**Fig. S58**. HRMS spectrum of compound **5g**


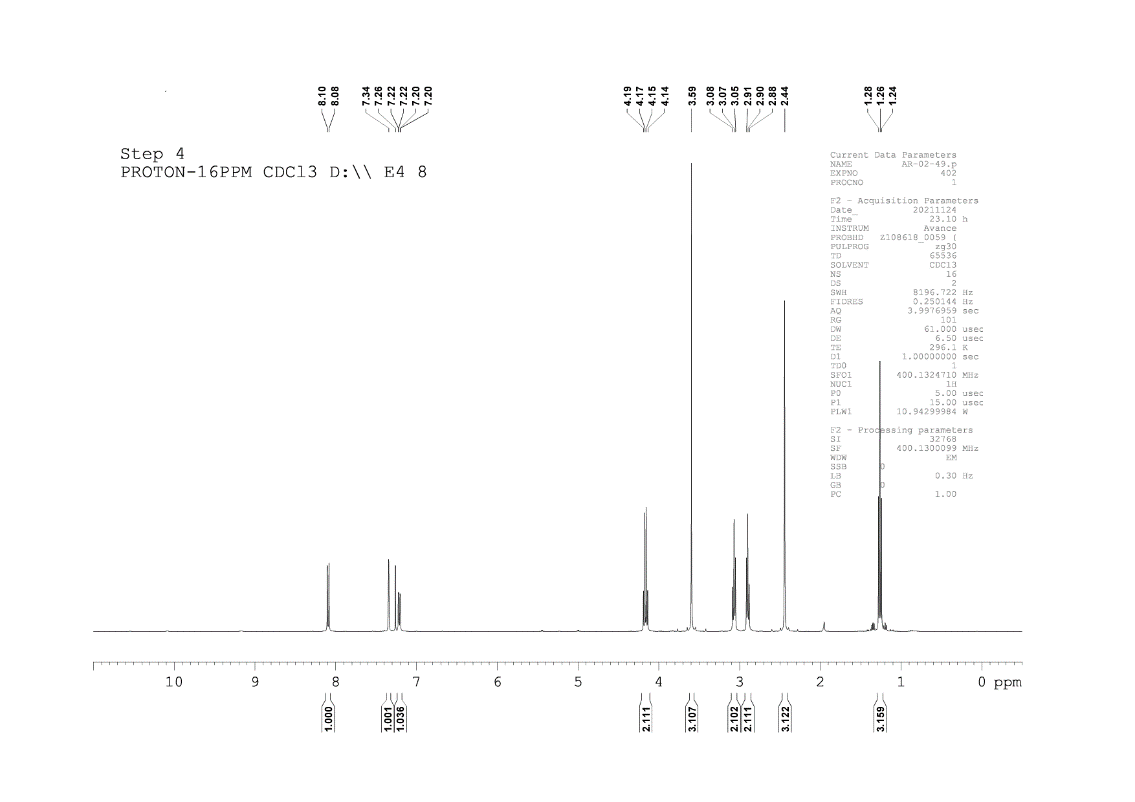


**Fig. S59**. ^1^H NMR (400 MHz, CDCl_3_) spectrum of compound **6g**


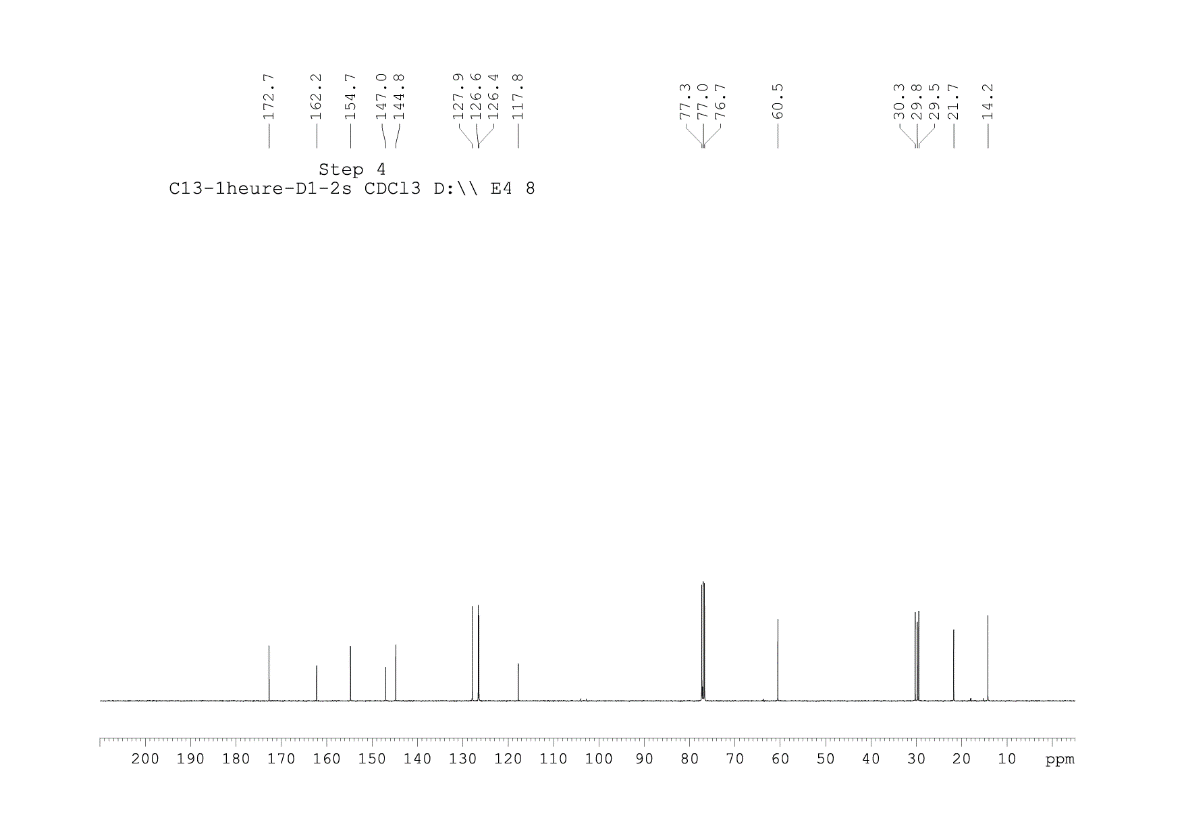


**Fig. S60**. ^13^C NMR (100 MHz, CDCl_3_) spectrum of compound **6g**


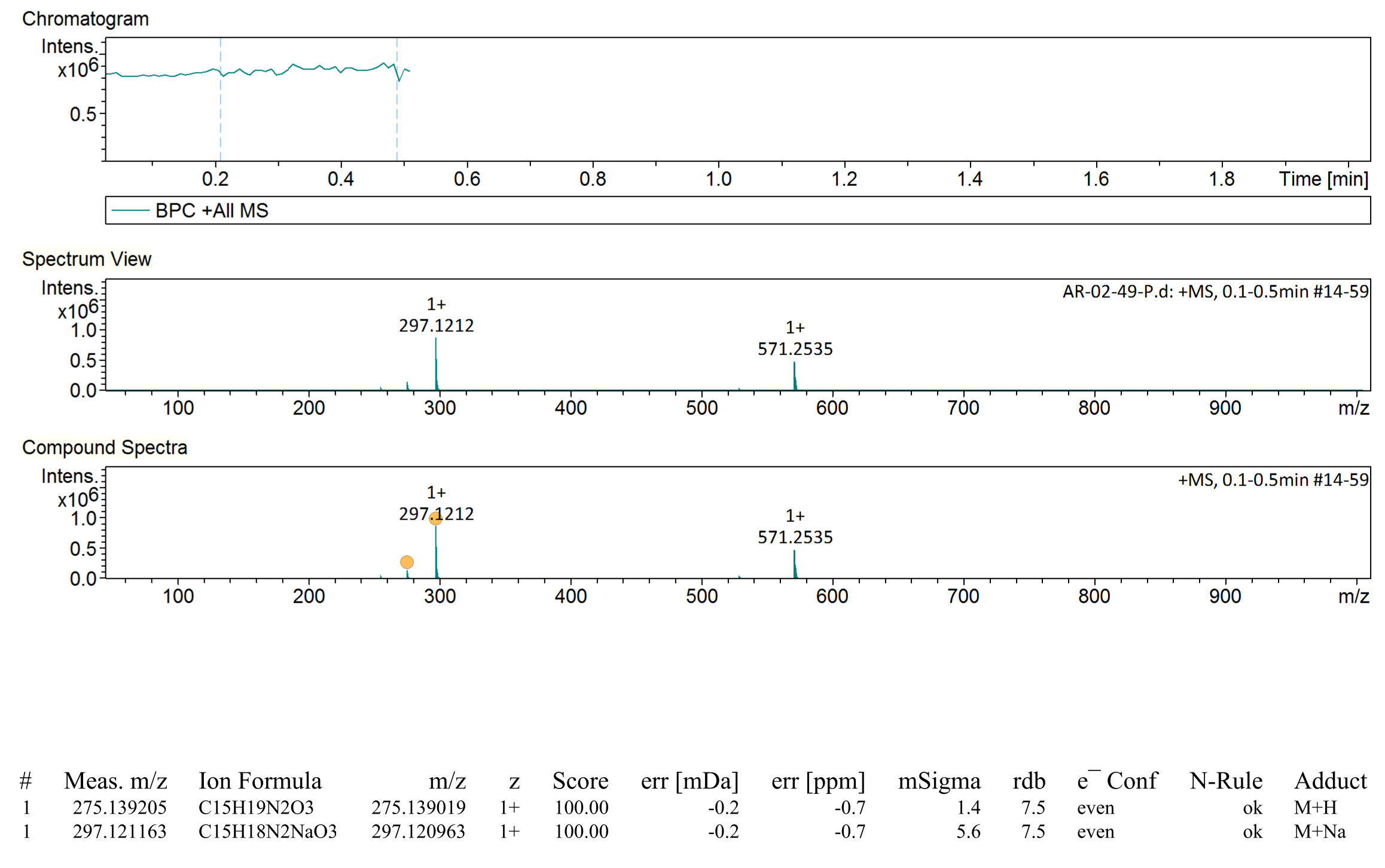


**Fig. S61**. HRMS spectrum of compound **6g**


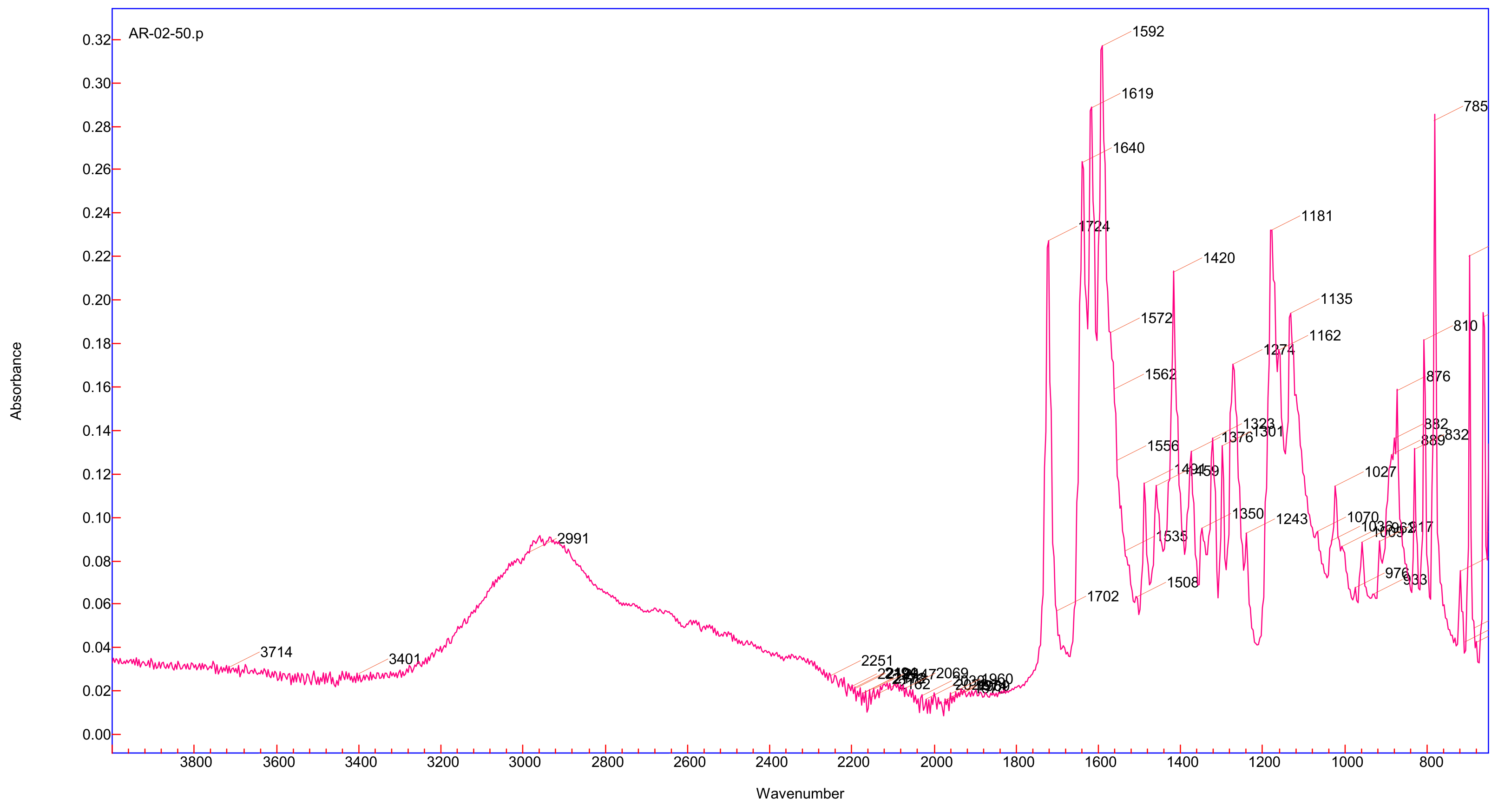


**Fig. S62**. IR spectrum of compound **1g**


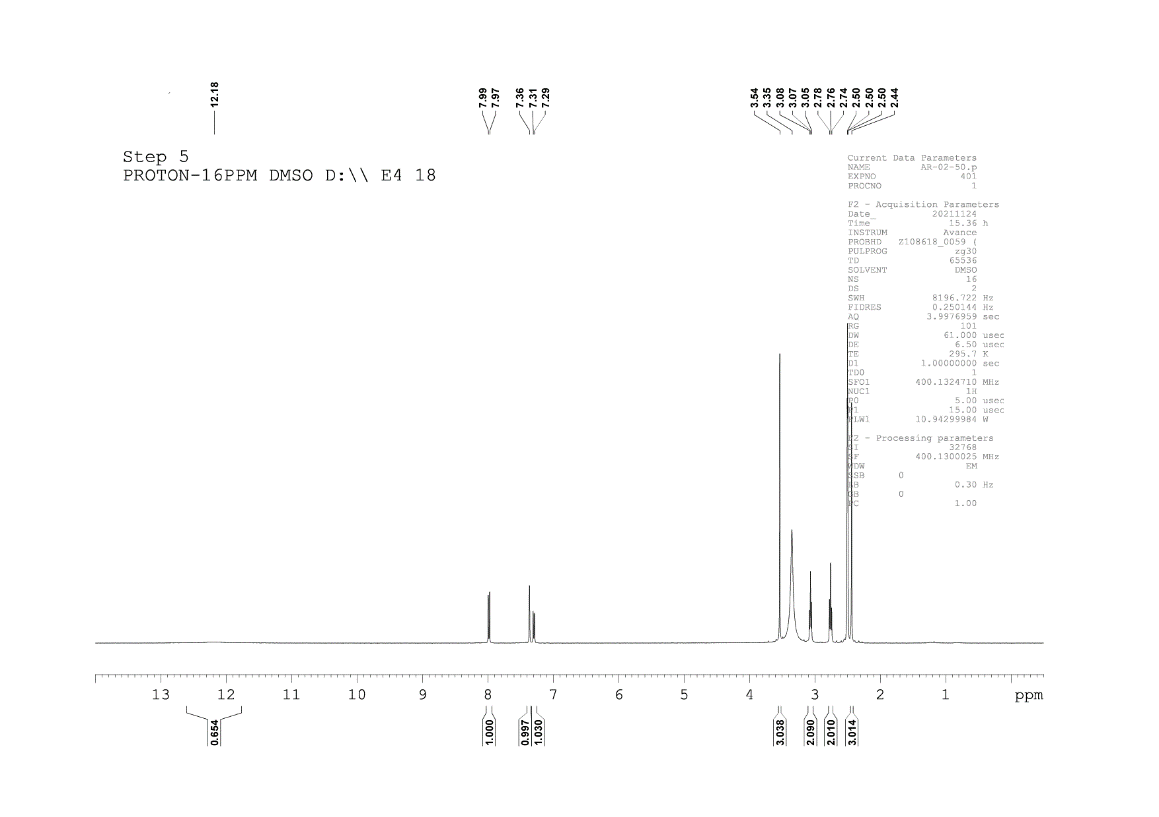


**Fig. S63**. ^1^H NMR (400 MHz, DMSO-*d_6_*) spectrum of compound **1g**


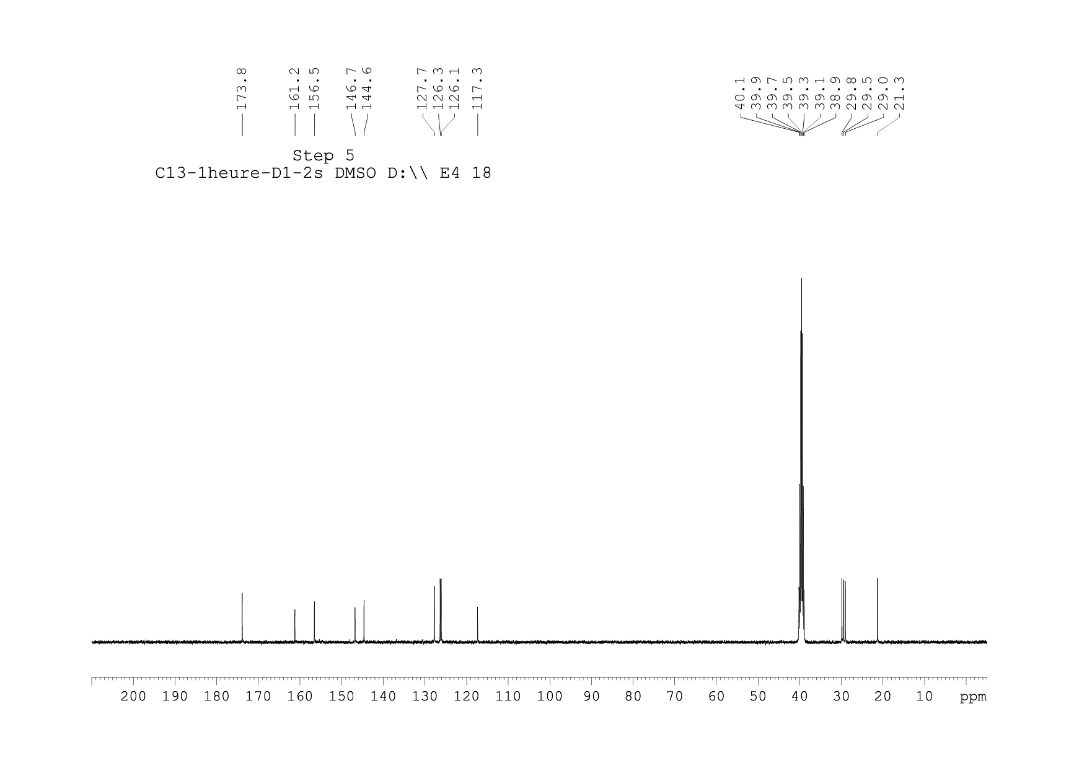


**Fig. S64**. ^13^C NMR (125 MHz, DMSO-*d_6_*) spectrum of compound **1g**


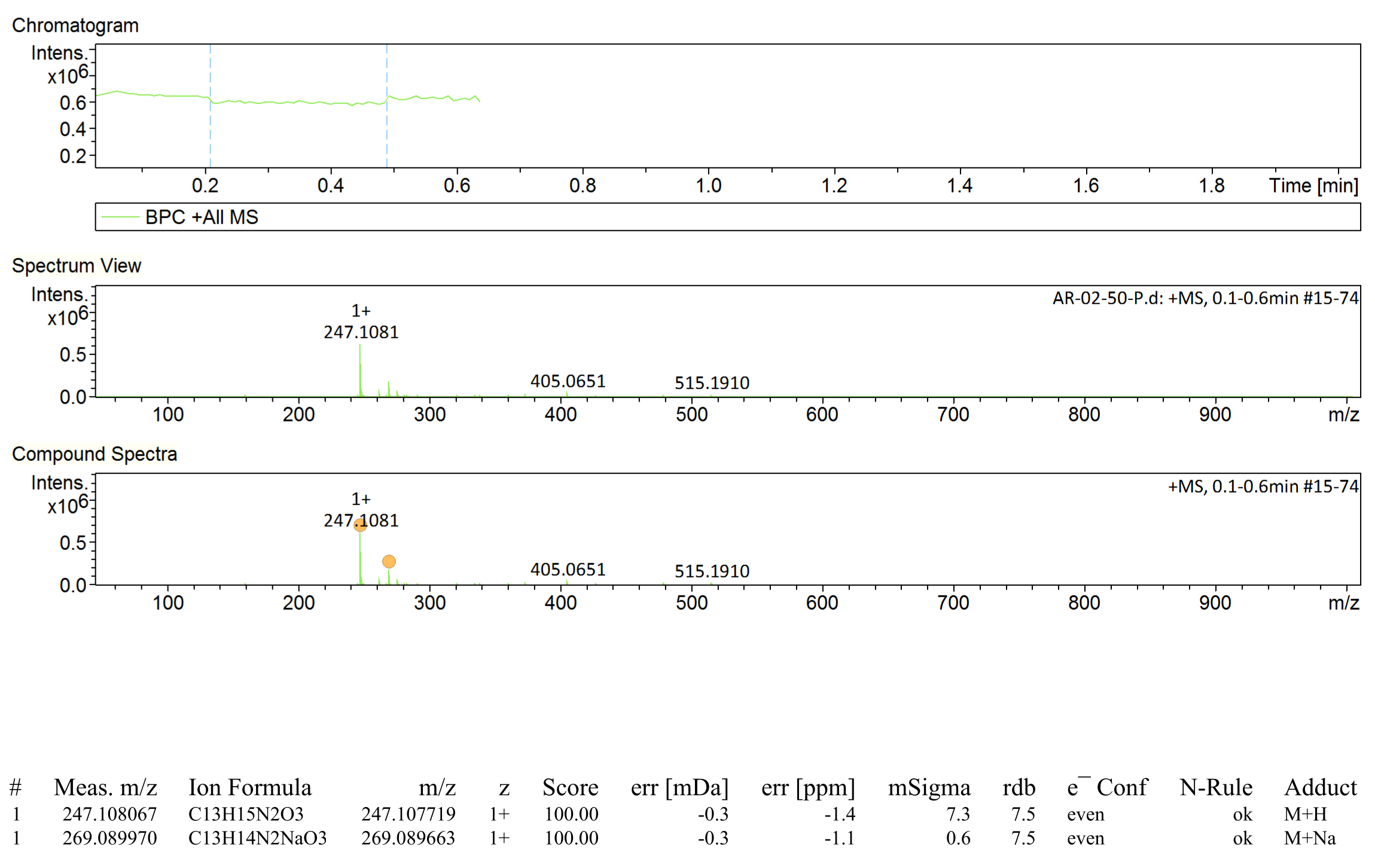


**Fig. S65**. HRMS spectrum of compound **1g**


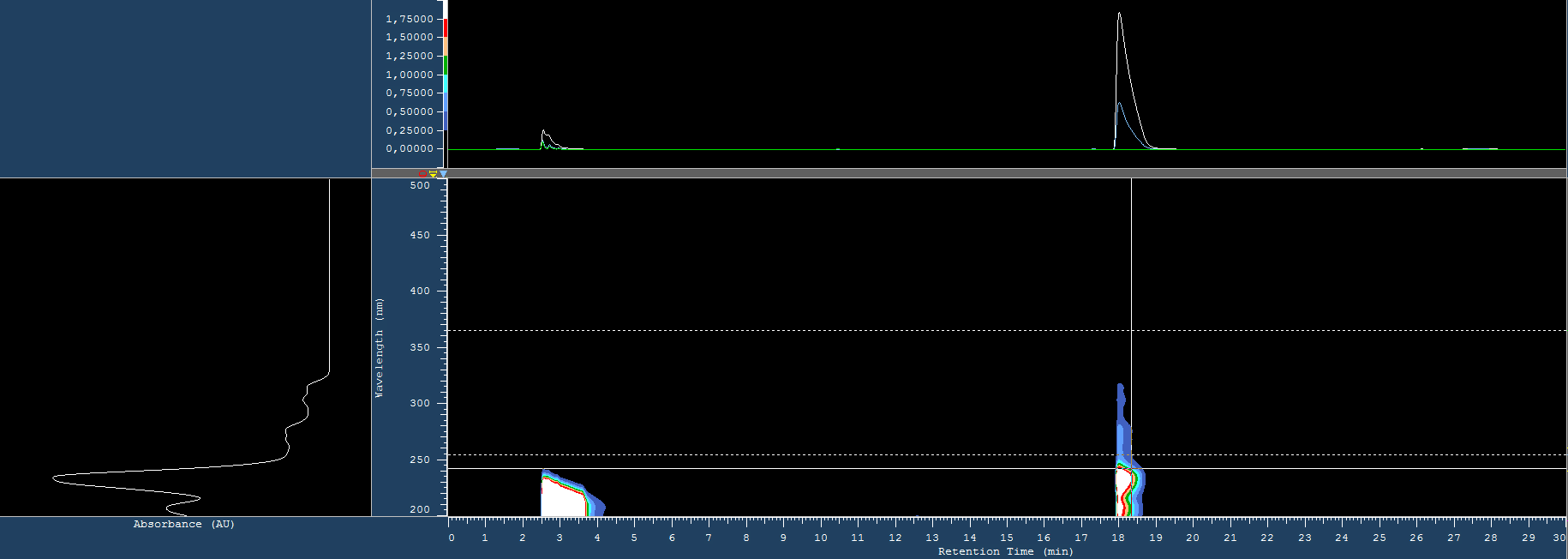


**Fig. S66**. HPLC-UV spectrum of compound **1g**
