# Supplementary material for: Selective molecular inhibition of the HDAC6 ZnF-UBP binding domain impairs multiple myeloma cell function
Source: Cell Death Discov. 2025 Apr 15;11:176. doi: 10.1038/s41420-025-02465-1 (PMC12000391; doi:10.1038/s41420-025-02465-1)
Supplement: Supplementary file 2 — Original data [file 41420_2025_2465_MOESM2_ESM.docx]

**Western blots**

**Experiment #1 – 1 gel/membrane to look at Ac-tubulin, HDAC6 and GAPDH, 2 different exposure time**

**GAPDH (36 kDa)**

**Ac-tubulin (Lys40, 52 kDa)**


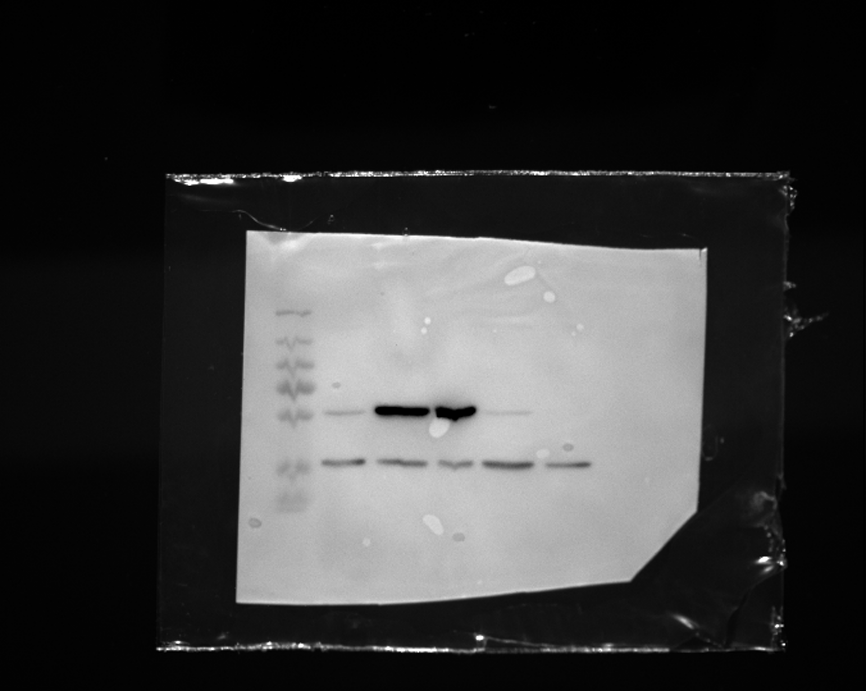


**180 kDa**

**130 kDa**

**100 kDa**

**70 kDa**

**55 kDa**

**40 kDa**

**35 kDa**

**WT**

**Ric**

**KO**

**RY**

**U**

**HDAC6 (130 kDa)**


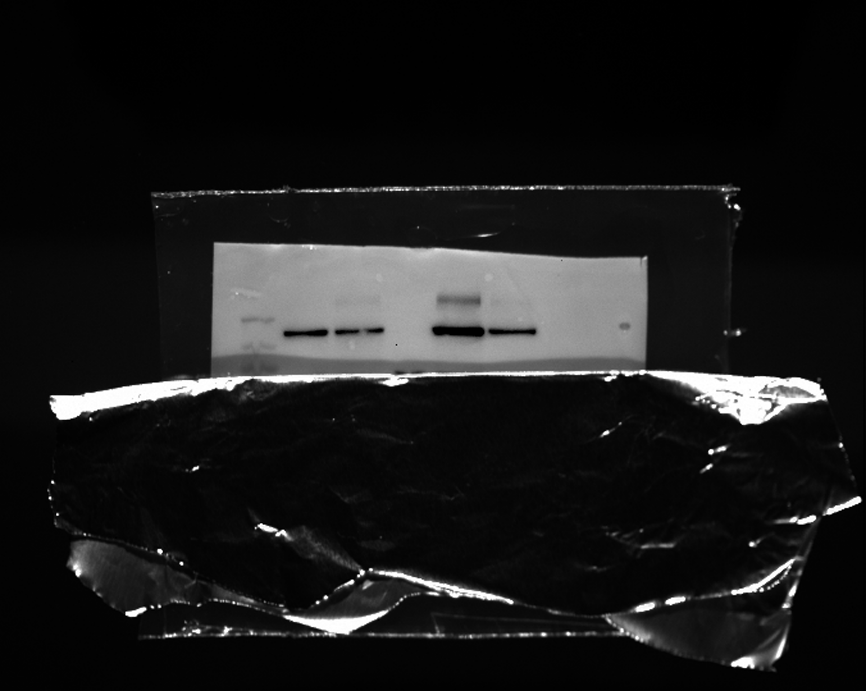


**180 kDa**

**130 kDa**

**WT**

**Ric**

**KO**

**RY**

**U**

**100 kDa**

Samples: RPMI 8226 cells (WT), RPMI 8226 cells treated with ricolinostat (2 µM, 24 h; Ric), RPMI 8226 HDAC6^KO^ cells (KO), RPMI HDAC6^RY^ cells (RY), unrelated sample (U).

**Experiment #2 – 1 gel/membrane to look at Ac-tubulin, HDAC6 and GAPDH, 2 different exposure time**

**RY**

**KO**

**Ric**

**WT**

**100 kDa**

**130 kDa**

**180 kDa**

**15 kDa**

**25 kDa**

**35 kDa**

**40 kDa**

**55 kDa**

**70 kDa**

**GAPDH (36 kDa)**


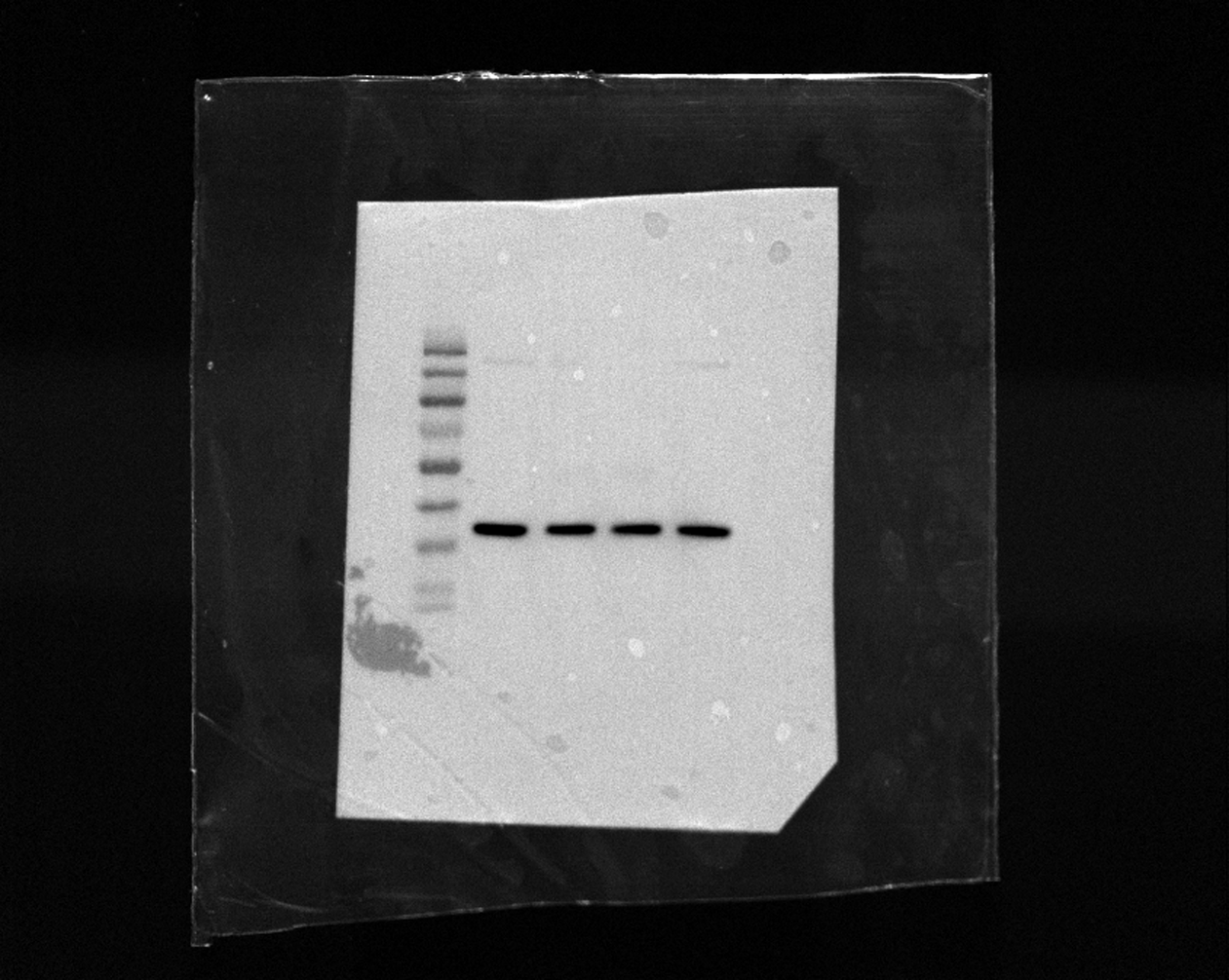


**WT Ric KO RY**

**180 kDa**

**130 kDa**

**100 kDa**

**70 kDa**

**55 kDa**

**40 kDa**

**Ac-tubulin (Lys40, 52 kDa)**

**HDAC6 (130 kDa)**


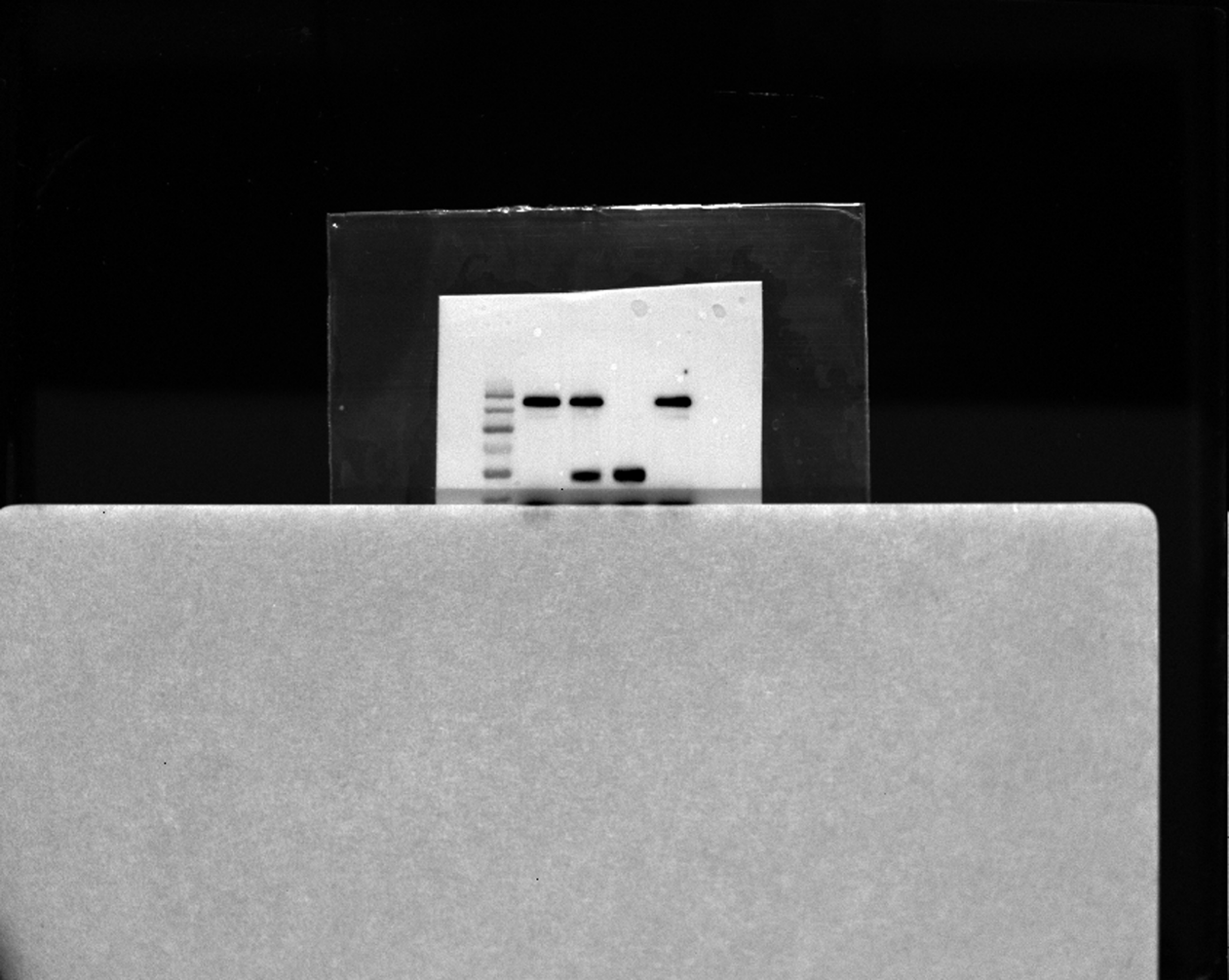


Samples: RPMI 8226 cells (WT), RPMI 8226 cells treated with ricolinostat (2 µM, 24 h; Ric), RPMI 8226 HDAC6^KO^ cells (KO), RPMI HDAC6^RY^ cells (RY).

**Experiment #3 – 1 gel/membrane to look at Ac-tubulin, HDAC6 and GAPDH, 2 different exposure time**

**WT Ric KO RY**

**180 kDa**

**130 kDa**

**100 kDa**

**70 kDa**

**55 kDa**

**40 kDa**

**35 kDa**

**GAPDH (36 kDa)**


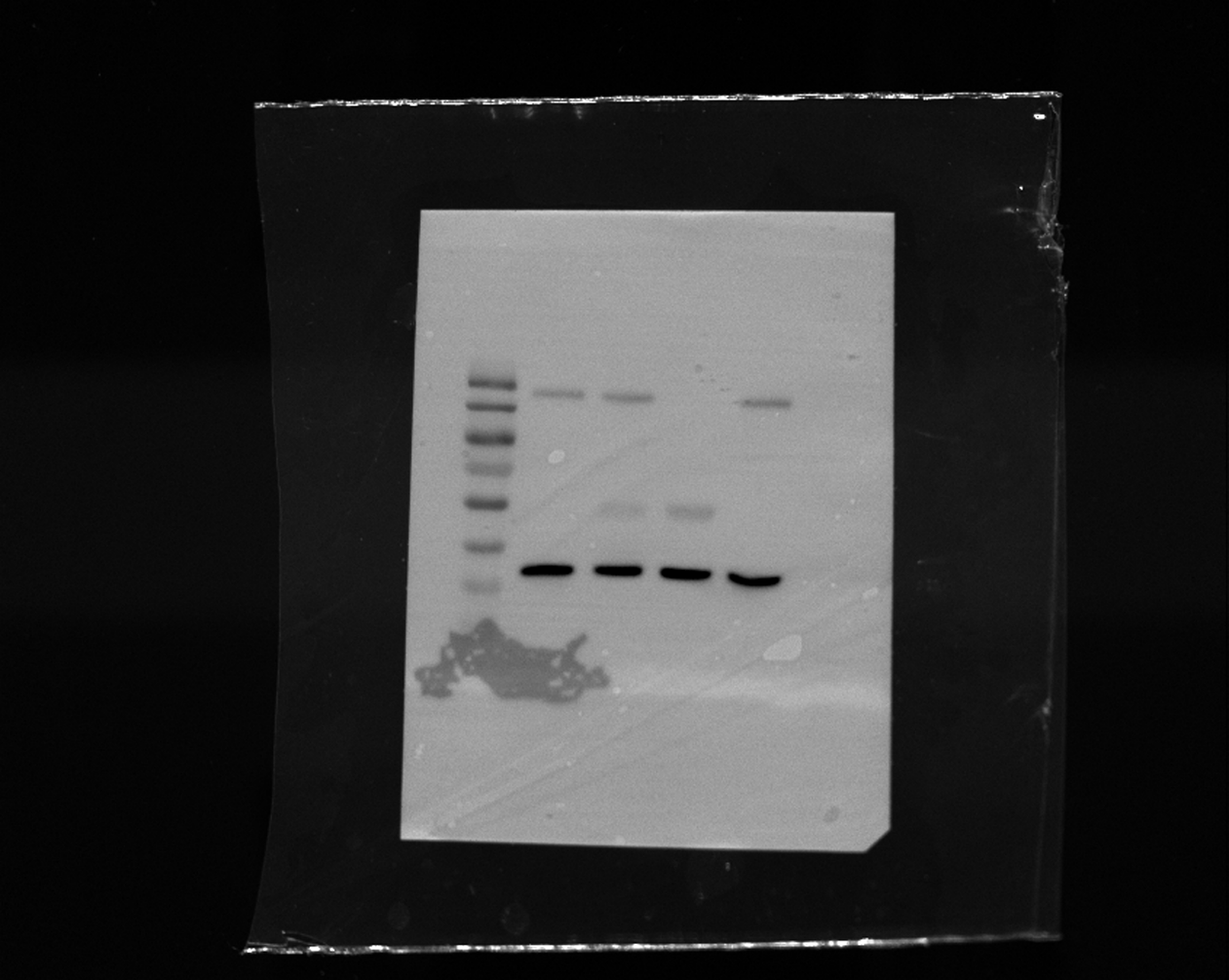


**180 kDa**

**130 kDa**

**100 kDa**

**70 kDa**

**55 kDa**

**40 kDa**

**WT Ric KO RY**

**Ac-tubulin (Lys40, 52 kDa)**

**HDAC6 (130 kDa)**


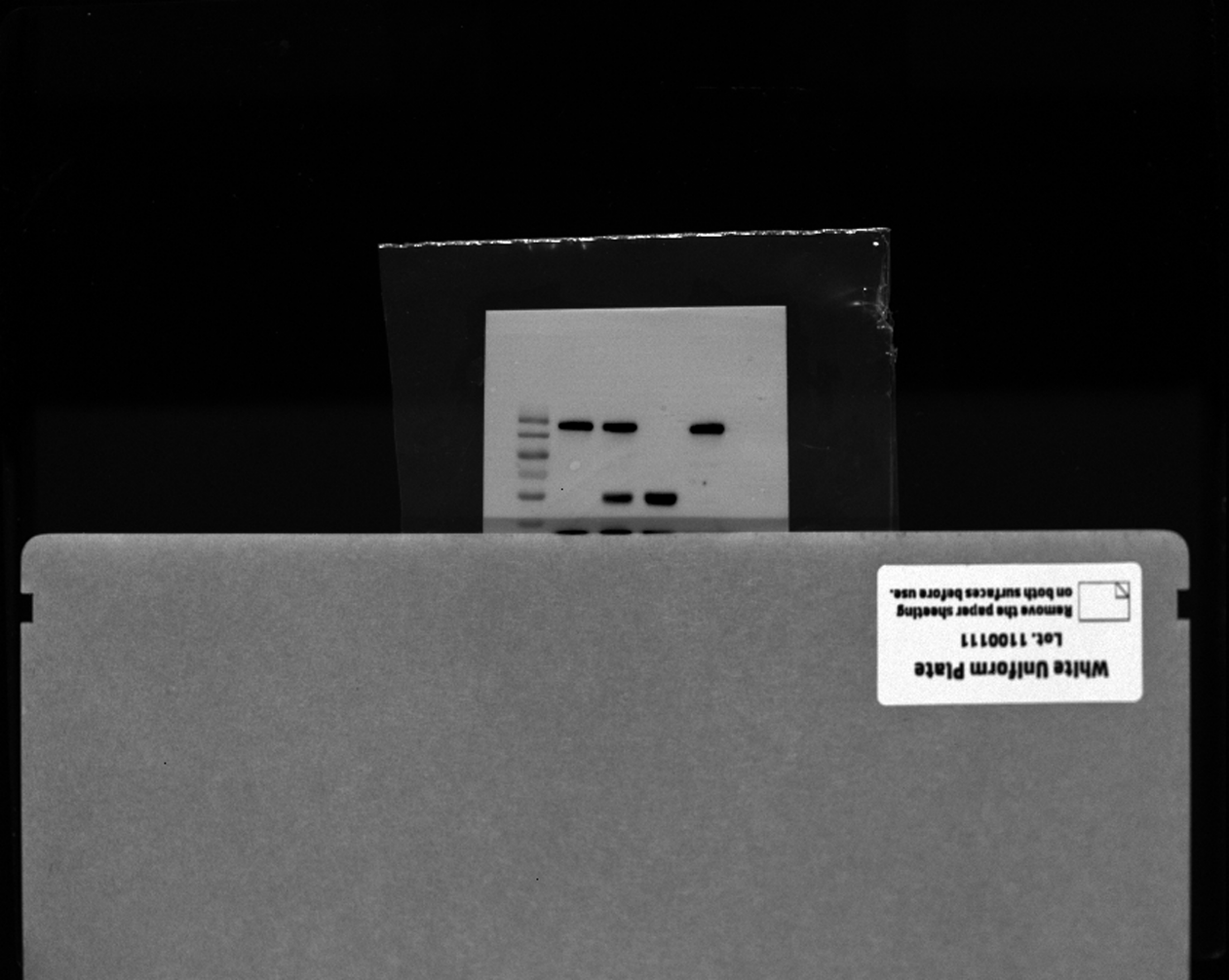


Samples: RPMI 8226 cells (WT), RPMI 8226 cells treated with ricolinostat (2 µM, 24 h; Ric), RPMI 8226 HDAC6^KO^ cells (KO), RPMI HDAC6^RY^ cells (RY).
